# Contact Electrification via Redox‐Active Molecules

**DOI:** 10.1002/anie.202510031

**Published:** 2025-11-19

**Authors:** Nisha Ranjan, Zohreh Izadi, Philipp Gaiser, María B. Camarada, Rekha Sharma, Andrej Weber, Michael Daub, Qiwei Hu, Michael Fiederle, Leonard Mayrhofer, Michael Moseler, Anna Fischer, Michael Walter, Birgit Esser, Bizan N. Balzer

**Affiliations:** ^1^ Institute of Physical Chemistry University of Freiburg Albertstr. 21 79104 Freiburg Germany; ^2^ Cluster of Excellence *liv*MatS @ FIT – Freiburg Center for Interactive Materials and Bioinspired Technologies University of Freiburg Georges‐Köhler‐Allee 105 79110 Freiburg Germany; ^3^ Institute of Organic Chemistry University of Freiburg Albertstr. 21 79104 Freiburg Germany; ^4^ Inorganic Functional Materials and Nanomaterials, Institute of Inorganic and Analytical Chemistry University of Freiburg Albertstr. 21 79104 Freiburg Germany; ^5^ Freiburg Center for Interactive Materials and Bioinspired Technologies University of Freiburg Georges‐Köhler‐Allee 105 79110 Freiburg Germany; ^6^ Institute of Organic Chemistry II and Advanced Materials Ulm University Albert‐Einstein‐Allee 11 89081 Ulm Germany; ^7^ Freiburg Materials Research Center (FMF) University of Freiburg Stefan‐Meier‐Str. 21 79104 Freiburg Germany; ^8^ Fraunhofer IWM Wöhlerstr. 11 79108 Freiburg Germany; ^9^ Institute of Physics University of Freiburg Hermann‐Herder‐Str. 3 79104 Freiburg Germany; ^10^ Max‐Planck‐Institut für Festkörperforschung Heisenbergstraße 1 70569 Stuttgart Germany; ^11^ Present address: Department of Materials Science and Engineering Division of Nanotechnology and Functional Materials, Uppsala University Box 35, SE‐751 03 Uppsala Sweden

**Keywords:** Atomic force microscopy, Contact electrification, Kelvin probe force microscopy, Redox activity, Surface functionalization, Triboelectricity, X‐ray photoelectron spectroscopy

## Abstract

Contact electrification, as the transfer of charge upon the contact of two (dis)similar materials, is strongly influenced by surface chemistry, which governs the efficiency of charge separation. For harvesting electrical energy from mechanical energy, material pairs with high electron‐transfer efficiency are essential. Here, we introduce a strategy to use electronic charge transfer in contact electrification via surface functionalization with redox‐active organic molecules. Specifically, we functionalize Au(111) surfaces with mercaptomethyl‐terminated redox‐active molecules, namely triphenylamine and tetrathiafulvalene as donors and 11,11,12,12‐tetracyano‐9,10‐anthraquinodimethane as an acceptor, achieving stable and covalent immobilization, as confirmed by X‐ray photoelectron spectroscopy, electrochemical characterization, and density functional theory calculations, and enabling molecular‐level electron‐transfer. To quantify charge transfer at the micrometer scale, we introduce a contact electrification assay combining atomic force microscopy‐based force spectroscopy and Kelvin probe force microscopy. This approach allows for a precise measurement of charge transfer between Au(111) surfaces functionalized with redox‐active molecules, revealing an electron‐driven mechanism capable of achieving surface charge densities of (120 ± 17) µC m^−2^. Our findings deepen the fundamental understanding of contact electrification by demonstrating that electron transfer—depending on the choice of materials—can indeed be its origin, and pave the way for the development of more efficient triboelectric devices.

## Graphical abstract

We present a novel molecular design strategy to perform electronic charge transfer during contact electrification by functionalizing surfaces with redox‐active organic molecules. To elucidate and quantify the underlying molecular‐level electron transfer processes, we combine X‐ray photoelectron spectroscopy, electrochemical analysis, density functional theory calculations, and an atomic force microscopy‐based contact electrification assay.

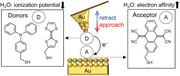

## Introduction

**Figure 1:**
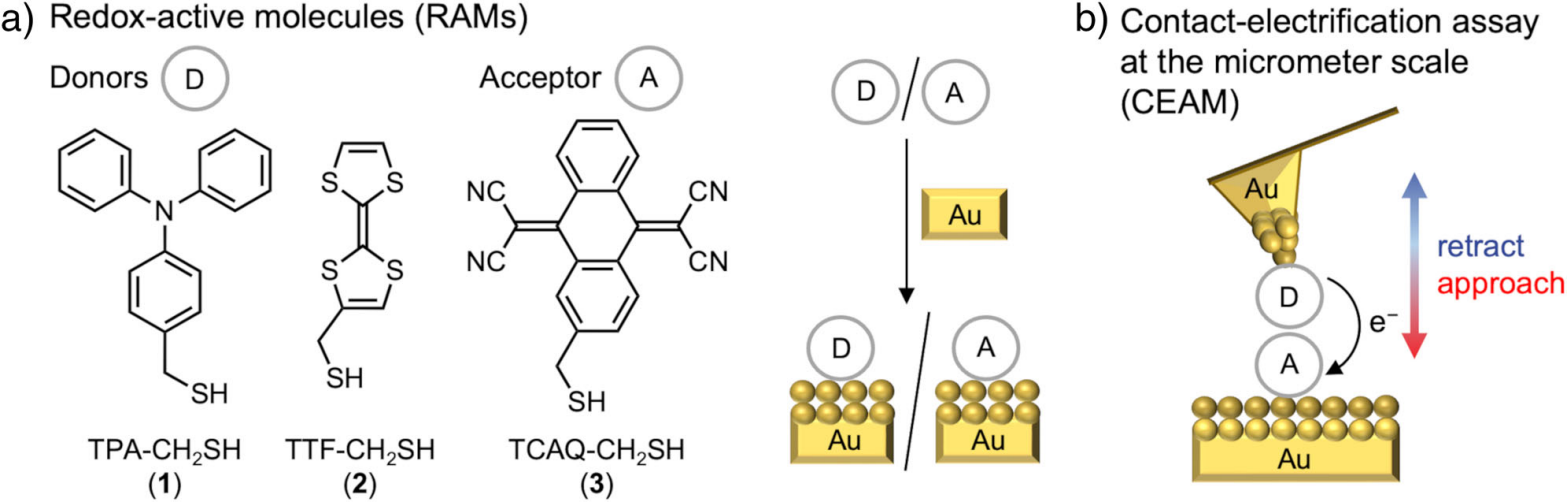
Concept of the redox‐active molecule (RAM)‐based contact electrification. a) RAMs (donors (D) or acceptor (A)) and their immobilization on Au surfaces; b) scheme of the AFM‐based contact electrification assay at the micrometer scale (CEAM) using RAMs as donor (D) and acceptor (A) molecules for functionalized Au cantilever tips and Au surfaces as contact partners.

The triboelectric effect, also called contact electrification, describes the transfer of charges upon contacting or mutual rubbing of two (dis)similar materials.^[^ ^1^, ^2^, ^3^, ^4^, ^5^ ^]^ It is a well‐known effect and has been investigated in a phenomenological way, as represented by the triboelectric series.^[^ ^1^, ^2^, ^6^, ^7^ ^]^ However, the mechanism of charge transfer, i.e., whether electrons, ions or materials are transferred, is not fully understood, in particular for insulators^[^ ^4^, ^6^, ^8^, ^9^ ^]^ and for chemically identical materials.^[^ ^10^, ^11^, ^12^, ^13^ ^]^ The breaking of surface contacts involves highly non‐adiabatic electronic and mechanical processes that inhibit the relaxation of a charge‐separated system to its equilibrium.^[^ ^11^, ^14^, ^15^ ^]^ Thus, charge transfer depends both on the specific surface chemistry of a material^[^ ^6^ ^]^ as well as on mechanical instabilities.^[^ ^11^ ^]^ Many physical or chemical modifications have been proposed for triboelectric charging, including chemical functionalization in the form of self‐assembled monolayers (SAMs).^[^ ^16^ ^]^ Such approaches include: functionalization with zwitterionic molecules,^[^ ^17^ ^]^ thiol‐SAMs (not redox‐active),^[^ ^18^, ^19^ ^]^ surface‐modified Al substrates,^[^ ^20^ ^]^ and surface patterning as physical modification.^[^ ^21^, ^22^ ^]^ We herein present a novel approach, in which we chemically modify surfaces with organic redox‐active molecules (RAMs) in order to make triboelectric charge separation more efficient. RAMs are able to reversibly donate or accept electrons, forming relatively stable doped states. We select well‐matched couples of organic RAMs of opposite polarity to perform contact electrification (Figure 1a).

In order to quantify the effect of the RAM functionalization on contact electrification, we introduce an atomic force microscopy (AFM)‐based contact electrification assay at the micrometer scale (CEAM, Figure 1b) as a model system for a proof‐of‐principle of our concept. AFM serves as a surface‐sensitive method at the micro‐ and nanometer scale to deliver surface‐potential information via Kelvin probe force microscopy (KPFM)^[^ ^23^, ^24^, ^25^, ^26^, ^27^ ^]^ and has been used to study triboelectricity.^[^ ^28^, ^29^ ^]^ Additionally, an AFM can be used for contacting via force‐extension curves.^[^ ^30^ ^]^ Our novel assay allows us to contact two (functionalized) surfaces with controlled contact parameters under controlled environmental conditions at the micrometer scale. KPFM is used to quantify the surface potential change, which accounts for the number of separated charges due to contact electrification. Yet, quantification of surface potentials via KPFM requires a proper reference, ideally within the same image. This is why we have designed a structured Au/SiO_2_ sample, wherein two adjacent Au stripes are imaged at once: a first Au stripe used for the contacting process and a second Au stripe serving as a reference.

As RAMs for this study, we have chosen organic molecules of opposite polarity, namely electron donors (D) with low ionization potential (IP) and electron acceptors (A) with high electron affinity (EA), with the potential for electron transfer.^[^ ^31^, ^32^, ^33^, ^34^ ^]^ Organic D–A pairs have been studied in chemistry for many years, with a focus on electronic interactions. This for instance resulted in the development of charge‐transfer complexes and/or salts that exhibit electrically conducting, superconducting, and magnetic properties.^[^ ^35^ ^]^ Here, we exploit such D–A pairs for triboelectric charge separation. As donors (D) we have chosen triphenylamine (TPA) and tetrathiafulvalene (TTF),^[^ ^36^, ^37^, ^38^ ^]^ and as acceptor (A) 11,11,12,12‐tetracyano‐9,10‐anthraquinodimethane (TCAQ, Figure 1a).^[^ ^39^, ^40^ ^]^ All are well‐established RAMs, known for either their donor (relatively low IP) or acceptor (relatively high EA) ability (see also Results and Discussion section).

For the CEAM assay, we immobilize these RAMs on Au surfaces as well as on Au AFM cantilever tips and investigate electron‐transfer processes upon contacting (Figure 1b). One of these is functionalized with a donor and the other with an acceptor, such that a significant increase in the number of transferred charges is obtained upon contacting.^[^ ^41^, ^42^ ^]^ The chosen RAMs have short molecular lengths of approximately 1–2 nm and frontier molecular orbitals that are close in energy to the Fermi levels of typically used electrode materials (e.g., Au, Ag, and Pt).^[^ ^43^, ^44^ ^]^ We use Au‐thiol chemistry^[^ ^45^, ^46^ ^]^ for covalent binding of the RAMs to Au surfaces with mercaptomethyl linkers (CH_2_SH), resulting in compounds **1**, **2**, and **3** as RAM‐CH_2_SH (Figure 1a). The saturated ─CH_2_─ bridge inhibits conjugation between the S lone pairs and the π‐system of the RAM and only has a minor influence on the frontier molecular orbital energies of the RAMs. Our model system, consisting of RAMs immobilized on Au‐covered substrates in N_2_ or air atmosphere, is designed not to undergo any further chemical reactions, such as redox reactions or the formation of radicals.^[^ ^47^, ^48^, ^49^ ^]^ Thus, we can fully focus on the charge‐transfer process between pairs of RAMs upon contacting. With this, we aim to provide a proof‐of‐principle study for contact electrification using pairs of RAMs.

## Results and Discussion

**Figure 2:**
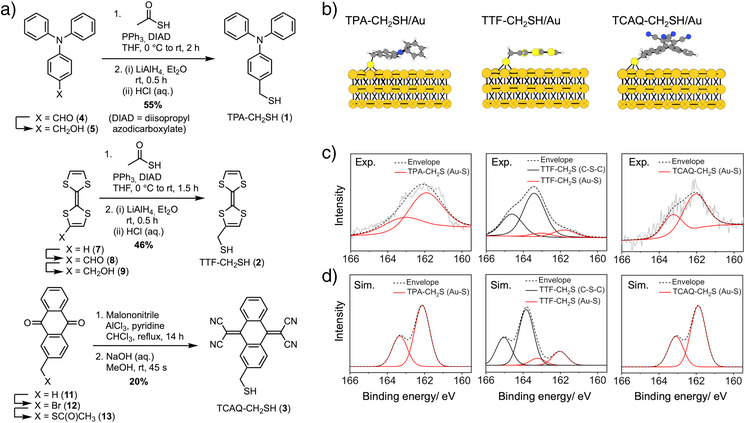
Synthesis of RAM‐CH_2_SH and characterization of their binding to Au. a) Synthetic routes to TPA‐ (**1**), TTF‐ (**2**), and TCAQ‐CH_2_SH (**3**); b) DFT‐predicted binding of RAM‐CH_2_SH to and orientation on the Au(111) surface. The thiol preferably binds on a hollow site on the Au surface and the RAMs lie flat on the Au surface; c) experimental and d) DFT‐predicted XPS intensity versus binding energy spectra of RAM‐CH_2_SH, immobilized on an Au surface. Both spectral envelopes and the deconvoluted peaks are given. The black doublet peak in the TTF‐CH_2_SH/Au spectrum represents C─S─C bonds. Each doublet deconvolution represents the spin‐orbit coupling of the S 2p orbital, S 2p_3/2_ and S 2p_1/2_ with a peak area ratio 2:1 and a splitting energy of 1.2 eV. Theoretical spectra are folded by Gaussians with 0.94 eV as the full width at half maximum value.

The RAMs functionalized with mercaptomethyl linkers (RAM‐CH_2_SH) are synthesized as shown in Figure 2a. TPA‐CH_2_SH (**1**) is synthesized from 4‐formyltriphenylamine (**4**) in three steps via TPA‐CH_2_OH^[^ ^50^ ^]^ (**5**) and the respective thioacetate **6** in 55% yield. Reaction of thioacetate **6** with LiAlH_4_ affords the free thiol TPA‐CH_2_SH (**1**). TTF‐CH_2_SH (**2**) is synthesized analogously in four steps from TTF (**7**), via formyl‐TTF **8**, TTF‐CH_2_OH (**9**) and thioester **10** following literature procedures.^[^ ^51^, ^52^ ^]^ TCAQ‐CH_2_SH (**3**) is synthesized in four steps from 2‐methylanthraquinone (**11**) via 2‐(bromomethyl)anthraquinone (**12**)^[^ ^53^ ^]^ and the anthraquinone‐based thioacetate **13**.^[^ ^54^ ^]^ Condensation of the carbonyl groups in **13** with malononitrile under Lewis‐acid catalysis with AlCl_3_ followed by basic cleavage of the thioacetate affords the free thiol TCAQ‐CH_2_SH (**3**). Further details on the synthesis and characterization can be found in the Supporting Information (Schemes S1–S3, Sections A1 and B2 for synthesis details and Figures S2–S24 for ^1^H NMR and ^13^C NMR data).

The electrochemical characterization of the RAM‐CH_2_SH via cyclic voltammetry (CV) in solution allows estimating their IPs or EA (see Supporting Information, Sections A1 and B3, Figure S25, and Tables S1–S3). Table 1 compares these IP and EA values to results from density‐functional theory (DFT) calculations^[^ ^55^ ^]^ based on the Perdew–Burke–Ernzerhof (PBE) exchange correlation energy approximation^[^ ^56^ ^]^ within a continuum solvent model^[^ ^57^ ^]^ (see Supporting Information, Sections A5 and B4, for further details), where excellent agreement is found. The EA of TCAQ‐CH_2_SH is lower than the IPs of the donors, such that no spontaneous charge transfer occurs for the solvated molecules in CH_2_Cl_2._ The energy difference is even larger in the gas phase. It reduces in aqueous environment, where spontaneous charge transfer requires an energy of 0.8 eV for the TPA‐TCAQ redox‐couple, but only 60 meV for the TTF‐TCAQ redox‐couple. A further significant reduction in IPs and an increase in EA in the order of 1 eV, each favoring electron transfer, is expected after attachment to an Au surface due to a polarization of the metallic surface, which stabilizes the charges in the adsorbates.^[^ ^58^, ^59^ ^]^

**Table 1:** Experimental (exp., from cyclic voltammetry)^a)^ and calculated (sim.)^b)^ ionization potentials (IP) and electron affinities (EA) of RAM‐CH_2_SH in eV.

	IP_sim_ vacuum	EA_sim_ vacuum	IP_exp_ DCM	EA_exp_ DCM	IP_sim_ DCM	EA_sim_ DCM	IP_sim_ H_2_O	EA_sim_ H_2_O
TPA‐CH_2_SH	6.52	–	5.27	–	5.10	–	4.87	–
TTF‐CH_2_SH	6.07	–	4.68	–	4.42	–	4.13	–
TCAQ‐CH_2_SH	–	3.32	–	3.92	–	4.06	–	4.07

^a)^ Obtained in CH_2_Cl_2_ as solvent and internally referenced against ferrocene/ferrocenium (Fc/Fc^+^). The values are shifted by 4.76 eV^[^ ^60^ ^]^ to reference the vacuum level.

^b)^ Calculations (sim.) are done in three different environments: gas phase (vacuum), CH_2_Cl_2_ (DCM), and H_2_O.

Experimentally, we immobilize the RAM‐CH_2_SH molecules on Au(111) surfaces under inert conditions at room temperature (fully Au‐coated and Au/SiO_2_‐patterned CEAM substrate, see Supporting Information, Sections A2–A4 and B1). The Au‐substrate cleaning and characterization via X‐ray diffraction (XRD), scanning‐electron microscopy (SEM), and X‐ray photoelectron spectroscopy (XPS) are described in the Supporting Information (Sections B9 and B10 and Figures S41 and S42).

DFT calculations also shed light into the conformations of the RAM‐CH_2_SH molecules immobilized on Au(111) surfaces (Figure 2b and Supporting Information, Section B5 and Figures S27–S30 for convergence tests). The flexible ─CH_2_SH linker allows for different conformations (Figures S26 and S32–S34), where we find a flat orientation to have an approximately 1 eV lower energy than an upright orientation for any of the RAM‐CH_2_SH (Figure 2b). A flat orientation of TTF on Au(111) was observed in gas‐phase STM studies^[^ ^61^ ^]^ and aligns with configurations of other organic molecules on Au surfaces, even in aqueous environments.^[^ ^61^ ^]^ Furthermore, scratching experiments (Figure S48) align with this prediction. A comparison of binding energies within different unit cells leads to a predicted maximum surface coverage of ca. 0.4 molecules nm^−2^ (Figure S37).

High‐resolution XPS scans (energy resolution of 0.05 eV) allow us to delineate the bonding of the RAM‐CH_2_SH to Au. The spectra are taken with respect to S 2p core‐level spectra (Figure 2c; for survey scans see Supporting Information, Section B10, and Figure S43, and for comparison to C_12_H_25_SH see Figure S44). For comparison, DFT is used to simulate the spectra on the absolute energy scale^[^ ^62^ ^]^ (Figure 2d; for details, see Supporting Information, Sections B5 and B10). We find an excellent agreement both for the peak positions as well as for the spectral shapes between experiment (Figure 2c) and theory (Figure 2d), confirming successful functionalization. The S 2p_3/2_ peaks at 162.10 eV for TCAQ‐CH_2_SH and 161.97 eV for TPA‐CH_2_SH (Figure 2c) relate to the formation of an Au–S bond, the DFT prediction for the binding energy of the molecule lying flat on the surface indicates that the binding energy of the S 2p_3/2_ peak for TCAQ‐CH_2_SH is 161.89 eV and for TPA‐CH_2_SH is 162.13 eV (Figure 2d). For TTF‐CH_2_SH, the S 2p spectra exhibit two deconvoluted doublets. The S 2p_3/2_ peaks at 161.79 and 163.41 eV relate to the Au─S and in‐plane C─S─C of the TTF‐CH_2_SH molecule, respectively (Figure 2c). The DFT‐simulated S 2p_3/2_ binding energies indicate that the Au─S is found at an energy of 162.03 eV, while the in‐plane C─S─C is at 163.83 eV, closely matching the experimental values (Figure 2d). The theoretically determined XPS‐based binding energies are hardly affected by the respective orientations of the RAMS, disallowing this characterization by XPS (Figures S35 and S36).

Unbound and unreacted RAMs can be excluded as their spectra would be clearly distinguishable (Figure S31). Furthermore, high‐resolution C 1s and N 1s XPS scans further confirm the presence of the RAM‐CH_2_SH molecules (Figure S45), as well as AFM imaging (see Supporting Information, Section B11, and Figures S46 and S48), static H_2_O contact angle experiments (see Supporting Information, Section B11, and Figure S45) and electrochemical characterization (see Supporting Information, Section B12, and Figures S49 and S50) of RAM‐CH_2_SH immobilized on Au. An analysis of the redox‐active states (Figures S38–S40) proofs that covalent bonding to the Au surface does not affect the redox‐activity.

We next present the CEAM assay, which allows contacting of two (functionalized) surfaces with controlled contact parameters (contact force, contact time, unloading velocity) under controlled environmental conditions (temperature and humidity) at the micrometer scale. KPFM is performed to determine the surface potential change, which accounts for the number of separated charges due to contact electrification.

KPFM is an AFM‐based tool used to quantify the local surface potential. It uses an AC voltage applied to a KPFM cantilever tip made of Pt, which induces an oscillating electrostatic force. This force varies depending on the contact potential difference *V*_CPD_ between the KPFM cantilever tip and the underlying sample. The AFM system applies a compensating DC voltage to cancel the oscillating force. When the force is zeroed, the applied DC voltage equals the *V*_CPD_. By scanning the tip across the surface and recording the voltage needed to zero the force at each point, the system creates a map of the local *V*_CPD_. In our AFM system, KPFM is performed as a two‐pass technique. First, it acquires a topography image. Second, based on the topographical information, a *V*_CPD_ image is taken at constant height above the sample surface (nap mode).

**Figure 3:**
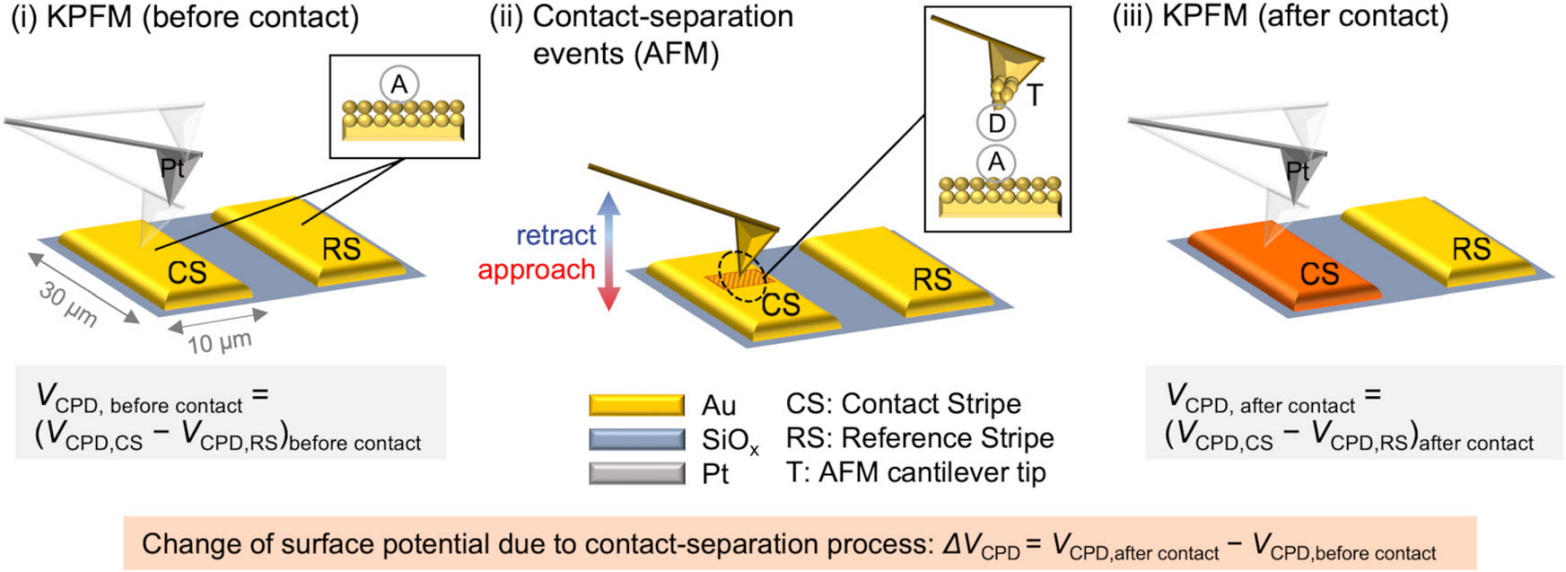
Scheme of the CEAM assay. a) CEAM is performed in a three‐step procedure: (i) KPFM imaging of an Au/SiO_2_‐based sample with a (functionalized, A: acceptor molecule) Au pattern (stripes) using a KPFM cantilever tip. (ii) contact‐separation process on one of the Au stripes (contact stripe, CS) with a (functionalized, D: donor molecule) Au AFM cantilever tip (T) by taking force‐extension curves (approach and retract events) in a grid‐like fashion (force map) with control of contact force, contact time, unloading velocity and environmental conditions (relative humidity and temperature). The second Au stripe is not contacted and serves as a reference stripe (RS). (iii) KPFM imaging after the contact‐separation process, wherein the change of the surface potential due to the contact‐separation process, Δ*V*_CPD_, is determined.

Our CEAM assay uses an Au/SiO_2_‐structured substrate (Figure 3). The Au patterns of the CEAM substrate comprise pairs of stripes spaced apart by 10 µm, isolated against each other by a 500 nm SiO_2_ layer underneath the Au (100 nm) and Cr (10 nm) layers (Figure S1). As the quantification of surface potentials via KPFM requires a proper reference, ideally within the same image, our Au/SiO_2_‐structured substrate comprises two adjacent Au stripes imaged at once: a first Au stripe for the contacting (CS) and a second Au stripe serving as a reference (RS). In the case of RAM‐functionalization, both the CS and RS are functionalized. However, in any case only the CS is used for the contacting process. This substrate design allows for both KPFM measurements at the micrometer scale and controlled contacting. The contacting is performed by AFM with the following parameters: contact force ca. 10 nN, contact time ca. 5.0 s, unloading velocity 50.0 µm s^−1^, 14% relative humidity (RH) at a temperature of 22 °C and in N_2_ atmosphere. We apply a three‐step procedure to investigate the effect of RAM‐functionalization on charge separation. In step (i) we use KPFM to determine the contact potential difference *V*_CPD_ (before contact) between the KPFM Pt cantilever tip and CS and RS. Then, difference of *V*_CPD_ between CS and RS before the contact‐separation process is obtained, as *V*_CPD,before contact_. Step (ii) involves performing 400 contact‐separation events in a grid‐like fashion by AFM between the Au cantilever tip and only the CS. This Au cantilever tip differs from the Pt KPFM cantilever tip used in steps (i) and (iii) and could therefore be functionalized with RAMs. Thus, the contact‐separation process with the (functionalized) Au cantilever tip is only performed after an initial KPFM image in step (i), serving as a baseline measurement. The Au stripes have dimensions of 30 × 10 µm^2^, which allows us to take force maps on a scale much larger than the AFM cantilever tip radius (30 nm, see Supporting Information, Section A6) with non‐overlapping single contact areas. In step (iii) we measure *V*_CPD_ (after contact) for CS and RS via KPFM. Then, the difference of *V*_CPD_ for CS and RS after the contact‐separation process is determined, as *V*_CPD,after contact_. Finally, the change of the surface potential due to the contact‐separation process Δ*V*_CPD_ is determined as the difference of *V*_CPD,after contact_ and *V*_CPD,before contact_.

Thus, the Au patterns of the CEAM substrate allow us to selectively charge (functionalized) Au stripes (CS), while using an adjacent (functionalized) stripe as a reference (RS) within the same KPFM image. Furthermore, we do not use the same cantilever for KPFM and AFM‐based contacting, allowing for well‐defined experiments, for instance with functionalized Au stripes (CS and RS) and functionalized Au AFM cantilever tips (T).

**Figure 4:**
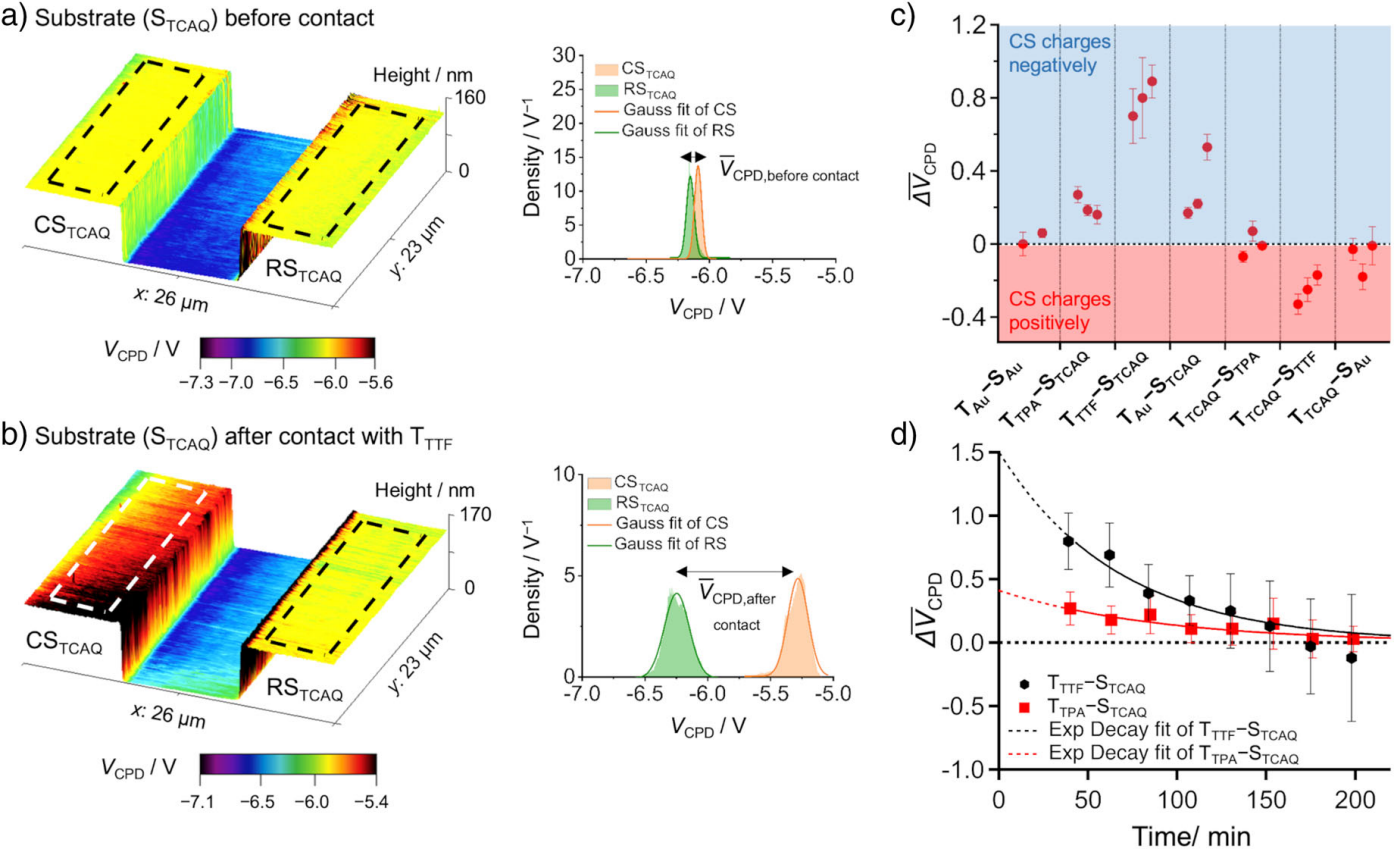
CEAM assay of RAM‐CH_2_SH/Au. a,b) Overlay of topography (height) and KPFM image of TCAQ‐CH_2_SH‐functionalized Au stripes (S_TCAQ_) before a contact‐separation process (a) and after a contact‐separation process with a TTF‐CH_2_SH‐functionalized Au AFM cantilever tip (T_TTF_) (b). *V*_CPD_ distributions ρ(*V*_CPD_) are given for both the CS and RS (a and b, right). c) Mean change of the surface potential due to the contact‐separation process ${\bar{\Delta V}}_{\mathrm{CPD}}$ for different (functionalized) tip (T) ‐substrate (CS) combinations, wherein each combination is taken three times using each time a different CS and RS pair, wherein step (iii) has usually been performed with a time delay of ca. 40 min after the final force‐extension curve of step (ii). The mean values are taken from the maxima of the Gaussian fits, and the error values are obtained by quadratic error propagation of the respective standard deviations of the Gaussian fits (see Supporting Information, Section A7). d) Time‐dependence of ${\bar{\Delta V}}_{\mathrm{CPD}}$ for T_TTF_‐S_TCAQ_ and T_TPA_‐S_TCAQ_ after the contact‐separation process shows an exponential decay. The dotted lines represent an extrapolation to 0, i.e., the time when the force map has been finished.

We use in the following RAM‐CH_2_SH/Au couples with Au AFM cantilever tips (T) and CS and RS Au stripes on the CEAM substrate, assigned by (S), for the contacting process: T_TPA_‐S_TCAQ_, T_TTF_‐S_TCAQ_ and T_Au_‐S_TCAQ_, wherein T_Au_–S_Au_ is a control showing no contact electrification. The areas (much larger than the KPFM cantilever tip radius of 15 nm, see Figure 4a,b and Supporting Information, Section A6) indicated by the broken lines in Figure 4a,b, are used for obtaining *V*_CPD_ distributions ρ(*V*_CPD_) for both the CS and RS, in order to omit any effects related to the topographic steps and KPFM cantilever tip shape.^[^ ^63^, ^64^ ^]^ There, the mean value ${\bar{V}}_{\mathrm{CPD},\mathrm{beforecontact}}$ in Figure 4a takes slight differences between CS and RS before the contact‐separation process into account. The mean value ${\bar{V}}_{\mathrm{CPD},\mathrm{aftercontact}}$ in Figure 4b shows whether the mean CS surface potential has changed significantly after the contact‐separation process. The difference of ${\bar{V}}_{\mathrm{CPD},\mathrm{aftercontact}}$ and ${\bar{V}}_{\mathrm{CPD},\mathrm{beforecontact}}$ is used to obtain the mean change of the surface potential due to the contact‐separation process ${\bar{\Delta V}}_{\mathrm{CPD}}$ (Figure 4a,b, and Supporting Information, Section A7).

Our measurements are summarized in Figure 4c. The largest ${\bar{\Delta V}}_{\mathrm{CPD}}$ is obtained for T_TTF_‐S_TCAQ_, followed by T_Au_‐S_TCAQ_ and T_TPA_‐S_TCAQ_. Here, ${\bar{\Delta V}}_{\mathrm{CPD}}$ has a positive sign, which means that the TCAQ‐functionalized Au stripe takes up negative charges through contact with the TTF‐, TPA‐functionalized or bare Au AFM cantilever tip, as expected for this acceptor. This trend follows the trend in donor ability of the RAMs, i.e., the order of IPs of the donors or Au cantilever tip with IP_TTF_ < IP_Au(111)_ = 4.8 to 5.3 eV^[^ ^65^, ^66^ ^]^ ≅ IP_TPA_ (c.f. Table 1). Hence, TTF most easily donates electrons, followed by Au(111) and TPA. This trend is confirmed by using a mirrored setting, where the CS/RS and tip functionalizations are switched (T_TCAQ_‐S_TPA_, T_TCAQ_‐S_TTF_, and T_TCAQ_‐S_Au_). Here, ${\bar{\Delta V}}_{\mathrm{CPD}}$ on average has a negative sign, which means that the TPA‐, TTF‐functionalized or bare Au stripe (CS) is positively charged after contact with the TCAQ‐functionalized Au cantilever tip. However, in this switched setting, lower absolute ${\bar{\Delta V}}_{\mathrm{CPD}}$ values are obtained, which could possibly result from a lower TCAQ‐based coverage of the curved AFM cantilever tip surface compared to the planar Au surfaces on the CEAM substrate.^[^ ^67^ ^]^

Based on the ${\bar{\Delta V}}_{\mathrm{CPD}}$ values, we can determine the mean surface‐charge‐density changes $\bar{\Delta \sigma }$ using the plate‐capacitor model^[^ ^68^ ^]^ (see Supporting Information, Section A7), wherein an increase in $\bar{\Delta \sigma }$ means an uptake of electrons for the investigated systems.^[^ ^2^ ^]^ As we neglect the influence of the AFM cantilever^[^ ^69^ ^]^ and of the KPFM cantilever tip shape^[^ ^63^, ^64^ ^]^ in the plate‐capacitor model, the surface‐charge densities herein represent a lower limit of the actual values. The obtained $\bar{\Delta \sigma }$ values (*n* = 3) lead to a mean value of (4.0 ± 1.4) e (100 nm)^−2^, i.e., (64 ± 22) µC m^−2^, for T_TTF_‐S_TCAQ_ (see Supporting Information, Section B13, and Table S5).

Practically no spatial dependence of Δ*V*_CPD_ is found in the KPFM measurements, e.g., for T_TTF_‐S_TCAQ_ (Figure 4b), despite the contact area being restricted to a small part of the CS (see Figure 3ii and Supporting Information, Section B13 and Figure S51e). This hints at an immediate distribution of the transferred charges to the complete CS, and agrees with the Hirshfeld analysis of charged RAM‐functionalized Au(111) in our simulations, where most of the charge (>80%) is directly transferred to the Au substrate from the RAM (see Supporting Information, Section B6, and Table S4).

The observed charge transfer is in accordance with an electron‐transfer process. According to the calculated IP values (see discussion above and Table 1), for both TPA‐TCAQ and TTF‐TCAQ redox couples, charge transfer is energetically more probable in an aqueous environment compared to vacuum or dichloromethane. Indeed, the existence of capillary bridges due to water molecules present between the tip and surface is observed in force‐extension curves (see Figure S51a for an exemplary curve), which hints at the presence of a water film on the (functionalized) Au surfaces. Although we cannot entirely rule out material transfer, the adhesion forces (Figure S51c,d, and Table S5) do not show any time‐dependent change, which would be expected once the tip picks up or loses material, as also confirmed by the topography information obtained in step (iii) of the CEAM assay (Figure S52). Hence, we conclude that a transfer of electrons is responsible for the experimental observations. Furthermore, we do not observe any degradation or delamination of the RAM functionalization at the scale of our experiment.

Electron transfer may also explain the exponential decay of ${\bar{\Delta V}}_{\mathrm{CPD}}$ with time observed for T_TTF_‐S_TCAQ_ and for T_TPA_‐S_TCAQ_ right after the contact‐separation process. With ${\bar{\Delta V}}_{\mathrm{CPD}}\;\left(t\right)={\bar{\Delta V}}_{\mathrm{CPD},0}\;\cdot e^{\frac{-t}{\tau }}$, where ${\bar{\Delta V}}_{\mathrm{CPD},0}$ is the initial mean change of the surface potential due to the contact‐separation process, *t* is the time and τ is the decay time constant, we obtain: T_TTF_‐S_TCAQ_: ${\bar{\Delta V}}_{\mathrm{CPD},0}$ = (1504 ± 210) mV, i.e., $\bar{\Delta \sigma }$ = (7.5 ± 1.0) e (100 nm)^−2^ = (120 ± 17) µC m^−2^, τ = (67 ± 10) mins and T_TPA_‐S_TCAQ_: ${\bar{\Delta V}}_{\mathrm{CPD},0}$ = (411 ± 74) mV, i.e, $\bar{\Delta \sigma }$ = (2.1 ± 0.4) e (100 nm)^−2^ = (33 ± 6) µC m^−2^, τ = (91 ± 19) mins. The highest ${\bar{\Delta V}}_{\mathrm{CPD},0}$ value is comparable to strongly triboelectrically active materials, such as cellulose, polyvinyl chloride (PVC), polytetrafluoroethylene (PTFE), polyurethane (PU) rubber, acrylonitrile butadiene styrene (ABS), clear polycarbonate (PC), and polystyrene (PS) showing triboelectric charge density magnitudes from 103 to 137 µC m^−2^, as determined according to Zou et al. against liquid mercury.^[^ ^2^ ^]^ An overview of triboelectric charge densities can be found in the literature,^[^ ^70^, ^71^, ^72^ ^]^ wherein the following systems can serve as benchmarks: Wang et al. used SAM‐functionalized Au substrates against fluorinated ethylene propylene (FEP), obtaining a triboelectric charge density of up to 140 µC m^−2^.^[^ ^18^ ^]^ Chang et al. investigated zwitterionic molecule dimethylethylammoniumpropane sulfonate (NDSB) based systems delivering a triboelectric charge density of up to 50 µC m^−2^.^[^ ^17^ ^]^ Shin et al. measured arylsilane‐terminated with halogens against aminated molecules, both functionalized onto polyethylene terephthalate (PET) films, generating a triboelectric charge density of up to 76 µC m^−2^.^[^ ^73^ ^]^ Jao et al. obtained a triboelectric charge density of up to 90 µC m^−2^ for PTFE against a chitosan‐glycerol film.^[^ ^74^ ^]^

The similarity in the decay‐time constants τ (Figure 4d and Supporting Information, Section B14 and Figures S53 and S54) hints toward a charge dissipation (diffusion or neutralization) process after the contact‐separation process (Figure S51e). This could also involve water molecules still present in the atmosphere during the CEAM experiment (14% RH, see Supporting Information, Section A7, for CEAM assay details).^[^ ^75^, ^76^, ^77^, ^78^, ^79^, ^80^ ^]^ A decrease as well as time‐dependent decay of the ${\bar{\Delta V}}_{\mathrm{CPD}}$, such as obtained in Figures 4d and S53–S56, for both 14% RH and 37% RH conditions, is known from charged Au surfaces when exposed to atmospheric humidity.^[^ ^81^ ^]^ There, a molecular water layer can be formed on the (functionalized) Au surface.^[^ ^82^, ^83^, ^84^, ^85^, ^86^ ^]^ Water can dissociate into H^+^ and OH^−^ ions, which act as charge carriers.^[^ ^87^, ^88^, ^89^, ^90^ ^]^ Next, charges, which are transferred between the RAM pairs, could leak into the SiO_2_ (underneath the Au of the CEAM substrate) and finally to the Si substrate underneath and to ground.^[^ ^91^, ^92^ ^]^ As the humidity increases, the conductivity of the atmosphere increases as well, and the charges could dissipate into the atmosphere.^[^ ^91^, ^93^, ^94^ ^]^ We assign the significant decrease of ${\bar{\Delta V}}_{\mathrm{CPD}}$ for both T_TTF_‐S_TCAQ_ and T_TPA_‐S_TCAQ_ when increasing the humidity from 14% RH to 37% RH to enhanced leakage and dissipation effects. Furthermore, H^+^ and OH^−^ ions might adsorb to the Au‐immobilized RAMs and change the effective capacitance and thus screen the real contact potential difference.^[^ ^91^, ^95^, ^96^ ^]^ Thus, the effectively obtained ${\bar{\Delta V}}_{\mathrm{CPD}}$ value might be lower than the real ${\bar{\Delta V}}_{\mathrm{CPD}}$, i.e., our CEAM assay provides a lower limit for the ${\bar{\Delta V}}_{\mathrm{CPD}}$.

However, the exact dependency of the contact‐separation of RAM electron‐transfer on the atmosphere requires further studies. This is crucial for future applications under varying environmental conditions, where both relative humidity and temperature play an important role.

## Conclusion

We herein present a new approach to efficient contact electrification by chemically modifying Au surfaces with organic redox‐active molecules (RAMs). We select well‐matched couples of organic RAMs of opposite polarity to perform contact electrification, namely triphenylamine, tetrathiafulvalene, and 11,11,12,12‐tetracyano‐9,10‐anthraquinodimethane.

In order to quantify the effect of surface functionalization on contact electrification, we introduce an AFM‐based contact‐electrification assay at the micrometer scale (CEAM). This involves the controlled contacting of two (functionalized) surfaces using an AFM and measuring changes in surface potential due to KPFM, thereby quantifying the degree of charge separation resulting from contact electrification. To that aim, we have engineered a structured Au/SiO_2_ substrate featuring Au stripes that can be selectively functionalized with RAMs. This configuration enables simultaneous surface potential mapping of two adjacent Au stripes: one used for contact electrification due to contact with a (functionalized) AFM cantilever tip, and the other serving as an unaltered reference. This setup allows for precise quantification of charge transfer following the contact between a RAM‐functionalized Au AFM cantilever tip and a functionalized Au stripe. Notably, the electron transfer between tetrathiafulvalene and 11,11,12,12‐tetracyano‐9,10‐anthraquinodimethane reaches values of (120 ± 17) µC  m^−2^.

Additionally, we are able to monitor the decay of surface potential on the electrified Au stripe over time, with charge dissipation occurring on a timescale of several tens of minutes. This dissipation process is likely influenced by atmospheric humidity. Interestingly, water also plays a crucial role in facilitating the charge transfer process, as it lowers the ionization potential of the donor and increases the electron affinity of the acceptor. This synergistic effect significantly enhances the thermodynamic favorability of charge transfer within the redox pair.

Our study delivers a proof‐of‐principle of contact electrification with suitable pairs of RAMs with clear physical and chemical origins of charge separation. This could introduce a new concept for triboelectrically active surfaces,^[^ ^30^ ^]^ which are of interest for triboelectric nanogenerators (TENGs).^[^ ^97^, ^98^, ^99^, ^100^, ^101^ ^]^ TENGs can harvest electrical energy from mechanical energy of the ambient environment, both at the small^[^ ^102^, ^103^ ^]^ and the large scale,^[^ ^104^, ^105^ ^]^ and therefore can be used as power sources for energy‐autonomous systems.^[^ ^106^, ^107^ ^]^ For efficient TENGs, the exact conditions of contacting, e.g., contact force, contact time, and environmental conditions, such as humidity^[^ ^75^, ^76^, ^108^ ^]^ and temperature,^[^ ^109^ ^]^ have to be optimized, and a high triboelectric surface‐charge‐density is required,^[^ ^110^, ^111^ ^]^ for instance, through an optimized choice of materials.^[^ ^2^, ^107^ ^]^

The use of RAMs as coatings on (soft and flexible) electrodes of TENGs provides novel materials‐design concepts for the integration of energy harvesting via contact electrification. Our study showcases that the use of RAMs is of interest for triboelectric applications and as part of materials, which can be used in macroscopic devices to deliver triboelectric charge separation at their interfaces. The charge transfer observed in our study can be explained purely by electron transfer from donor to acceptor molecules and is thus controlled by the redox properties of the RAMs.

Future investigations will have to study the impact of different types of substrates, humidity, contact time, contact force and unloading velocity at the macroscopic scale, paving the way to immobilizing RAMs onto soft substrates and flexible electrodes for use in materials systems. Thus, our results will foster the development of future materials systems with optimized charge‐separation capabilities for TENGs. In order to preserve the chemical functionality for TENG applications of the RAMs over a long period, low humidity conditions would be useful. They could be preserved by, e.g., a quartz cube,^[^ ^112^ ^]^ an ecoflex‐based^[^ ^113^ ^]^ or pouch type^[^ ^114^ ^]^ encapsulation. We would envision chemical RAM‐immobilization on a durable substrate, e.g., polydimethylsiloxane (PDMS), which can be scaled up in an affordable manner and topographically structured to maximize contact electrification.^[^ ^21^, ^22^, ^70^, ^71^, ^115^ ^]^ Furthermore, contact electrification could be controlled through mechanically induced deformation of electronic structures shifting donor and acceptor states.^[^ ^116^ ^]^ Instead of a molecular layer of RAMs, films or coatings of redox‐active polymers containing RAMs as part of their structure could be used to increase the packing density. Additionally, the optimization of the performance (e.g., power density) of such a TENG will require an increase of the effective contact area, e.g., by using a pair of topographically structured and commensurate surfaces. Then, we expect a very significant triboelectric charging at the macroscopic scale.

## Supporting Information

The authors have cited additional references within the Supporting Information.^[^ ^118^, ^119^, ^120^, ^121^, ^122^, ^123^, ^124^, ^125^, ^126^, ^127^, ^128^, ^129^, ^130^, ^131^, ^132^, ^133^, ^134^, ^135^, ^136^, ^137^, ^138^, ^139^, ^140^, ^141^, ^142^, ^143^, ^144^, ^145^, ^146^, ^147^, ^148^, ^149^, ^150^, ^151^, ^152^, ^153^, ^154^, ^155^, ^156^, ^157^, ^158^, ^159^ ^]^

## Conflict of Interests

The authors declare no conflict of interest.

## Supporting information



Supporting Information

## Data Availability

The data that support the findings of this study are available from the corresponding author upon reasonable request.
