# Supplementary material for: Contact Electrification via Redox‐Active Molecules
Source: Angew Chem Int Ed Engl. 2025 Nov 19;65(1):e10031. doi: 10.1002/anie.202510031 (PMC12759219; doi:10.1002/anie.202510031)
Supplement: Supplementary file 1 — Supporting Information [file ANIE-65-e10031-s001.pdf]

Supporting Information

for

Contact Electrification via Redox Active Molecules

Nisha Ranjan,†[a,b,j] Zohreh Izadi,†[b,i] Philipp Gaiser,†§[b,c] María B. Camarada,[b,d,e] Rekha Sharma,[b,c] Andrej Weber,[f] Michael Daub,[d] Qiwei Hu,[a,b] Michael Fiederle,[g] Leonard Mayrhofer,[h] Michael Moseler,[b,e,g,h,i] Anna Fischer,*[b,d,e,g] Michael Walter,*[b,e,g,h] Birgit Esser*[b,f] and Bizan N. Balzer*[a,b,g]

† These authors contributed equally to this work.

§ Present address: Department of Materials Science and Engineering, Division of Nanotechnology and Functional Materials, Uppsala University, Box 35, SE-751 03 Uppsala, Sweden.

[a] Dr. N. Ranjan, Dr. Q. Hu, Dr. B. N. Balzer
Institute of Physical Chemistry, University of Freiburg, Albertstr. 21, 79104 Freiburg, Germany

[b] Dr. N. Ranjan, Z. Izadi, P. Gaiser, Dr. M. B. Camarada, Dr. R. Sharma, A. Weber, Dr. M. Daub, Dr. Q. Hu, Prof. Dr. M. Fiederle, Dr. L. Mayrhofer, Prof. Dr. M. Moseler, Prof. Dr. A. Fischer, Dr. M. Walter, Prof. Dr. B. Esser, Dr. B. N. Balzer
Cluster of Excellence *liv*MatS @ FIT - Freiburg Center for Interactive Materials and Bioinspired Technologies, University of Freiburg, Georges-Köhler-Allee 105, 79110 Freiburg, Germany

[c] P. Gaiser, Dr. R. Sharma
Institute of Organic Chemistry, University of Freiburg, Albertstr. 21, 79104 Freiburg, Germany

[d] Dr. M. B. Camarada, Dr. M. Daub, Prof. Dr. A. Fischer
Inorganic Functional Materials and Nanomaterials, Institute of Inorganic and Analytical Chemistry, University of Freiburg, Albertstr. 21, 79104 Freiburg, Germany

[e] Dr. M. B. Camarada, Prof. Dr. M. Moseler, Prof. Dr. A. Fischer, Dr. M. Walter
Freiburg Center for Interactive Materials and Bioinspired Technologies, University of Freiburg, Georges-Köhler-Allee 105, 79110 Freiburg, Germany

[f] A. Weber, Prof. Dr. B. Esser
Institute of Organic Chemistry II and Advanced Materials, Ulm University, Albert-Einstein-Allee 11, 89081 Ulm, Germany

[g] Prof. Dr. M. Fiederle, Prof. Dr. M. Moseler, Prof. Dr. A. Fischer, Dr. M. Walter, Dr. B. N. Balzer
Freiburg Materials Research Center (FMF), University of Freiburg, Stefan-Meier-Str. 21, 79104 Freiburg, Germany

[h] Dr. L. Mayrhofer, Prof. Dr. M. Moseler, Dr. M. Walter
Fraunhofer IWM, Wöhlerstr. 11, 79108 Freiburg, Germany

[i] Z. Izadi, Prof. Dr. M. Moseler
Institute of Physics, University of Freiburg, Hermann-Herder-Str. 3, 79104 Freiburg, Germany

[j] Dr. N. Ranjan
Max-Planck-Institut für Festkörperforschung, Heisenbergstraße 1, 70569 Stuttgart, Germany

A. Materials and Methods 3
1. Redox-Active Molecule (RAM) Synthesis and Characterization 3
2. Au Substrate Cleaning and Characterization 4
3. RAM-CH_2SH Immobilization on Au Substrate and AFM Cantilever Tip 7
4. Characterization of RAM-CH_2SH Immobilization on Au Substrate 8
5. Density Functional Theory Simulations 10
6. Contact Electrification At the Micrometer Scale (CEAM) Assay of RAM-CH_2SH Immobilized Au Substrates 12
7. Data Evaluation for CEAM Assay 14
B. Supporting Data 18
1. Experimental Au Substrate 18
2. Synthesis and Characterization of CH_2SH-substituted RAMs 19
3. Electrochemical Characterization of RAM-CH_2SH via CV 47
4. DFT Calculations of Molecules 49
5. DFT Calculations of Functionalized Au(111) 51
6. Hirshfeld Charge Analysis 60
7. RAM Coverage on the Au surface 61
8. Projected Density of States of RAMs on the Au Surface 62
9. XRD and SEM of Au Substrate 66
10. XPS of RAM-CH_2SH Immobilized on Au Substrates 67
11. AFM Imaging and Static Contact Angles of RAM-CH_2SH Immobilized on Au 71
12. Electrochemical Characterization of RAM-CH_2SH Immobilized Au Substrates via CV 75
13. CEAM Assay 78
14. CEAM Assay: Time-Dependence of Charge Dissipation after Charge-Separation Process 81
C. References 83

A. Materials and Methods

1. Redox-Active Molecule (RAM) Synthesis and Characterization

Chemicals were purchased from ABCR, Acros-Organics, Alfa-Aesar, Sigma-Aldrich or TCI and used directly without further purification unless otherwise noted. Moisture- or oxygen-sensitive reactions were carried out in dried glassware, heated under vacuum (10^{-2} mbar), using standard Schlenk techniques in a dry argon atmosphere (Argon 4.6, MTI Industrie Gase). Anhydrous solvents (THF, Et_2O) were obtained from an M. Braun solvent purification system (MB-SPS-800) and stored over molecular sieves (3 Å). Other solvents were purchased and used in analytical or HPLC grade.

Analytical thin layer chromatography was carried out using silica gel-coated aluminum plates with a fluorescence indicator (Merck 60 F_{254} or Machery-Nagel ALUGRAM Xtra SIL G/UV_{254}). Detection was carried out by using UV light (λ_{max} = 254 nm or 366 nm).

Flash column chromatography was carried out using silica gel 60, grain size 40-63 μm (230-400 mesh) from Machery-Nagel.

Nuclear magnetic resonance (NMR) spectra were recorded at 300 K, unless otherwise noted, on a Bruker Avance Neo 400 spectrometer [400.1 MHz (^{1}H), 100.6 MHz (^{13}C)], Bruker Avance Neo 600 with a Prodigy CryoProbe [150.9 MHz (^{13}C)], Bruker Avance III HD [500.0 MHz (^{1}H), 125.7 MHz (^{13}C)]. Chemical shifts are reported in parts per million (ppm, δ scale) relative to the signal of tetramethylsilane (δ = 0.00 ppm). ^{1}H NMR spectra are referenced to tetramethylsilane as an internal standard or the residual proton signal of the respective solvent: $CDCl_3$: δ = 7.26 ppm; CD_2Cl_2: δ = 5.32 ppm. ^{13}C NMR spectra are referenced to the following signals: $CDCl_3$: δ = 77.16 ppm; CD_2Cl_2: δ = 53.84 ppm.[118] Analysis followed first order, and the following abbreviations for multiplets are used: singlet

(s), doublet (d), triplet (t), multiplet (m) and combinations thereof, i.e., doublet of doublets (dd). Coupling constants (*J*) are given in Hz.

High-resolution mass spectrometry (HRMS) was performed using a Fourier Transform Ion Cyclotron Resonance (FT-ICR) mass spectrometer solariX (Bruker Daltonik) equipped with a 7.0 T superconducting magnet and interfaced to an Apollo II Dual ESI/MALDI source, which can be switched from ESI to MALDI operation almost instantaneously. In MALDI operation mode 2-[(2*E*)-3-(4-*tert*-butylphenyl)-2-methylprop-2-enylidene]malononitrile (DCTB) was used as the matrix.

Cyclic voltammograms (CVs) **in solution** and differential pulse voltammograms (DPVs) were measured inside an argon-filled glovebox using a PGSTAT128N potentiostat (Metrohm Autolab). As working electrode, a glassy carbon disc electrode (2 mm diameter) was used, as counter electrode a Pt rod was used, as reference electrode an Ag/$AgNO_3$ electrode containing an Ag wire immersed in an inner chamber filled with 1 M $AgNO_3$ and 0.1 M tetrabutylammonium hexafluorophosphate (*n*-Bu_4NPF_6) in anhydrous CH_3CN or an Ag wire was used. The analyte solution contained 10 ml of solvent (anhydrous CH_2Cl_2 or THF) with 0.1 M *n*-Bu_4NPF_6 and the specified analyte concentration. The ferrocene/ferrocenium (Fc/Fc^+) redox couple was used as internal reference. HOMO and LUMO energy levels were estimated using the following equations: E_{LUMO} (eV) = $-(E_{\mathrm{i,Fc}}+E_{1/2,\mathrm{A}})$ ($E_{\mathrm{i,Fc}}$ = 4.76 eV is the ionization energy of ferrocene,[60] $E_{1/2,A}$ is the redox potential for the first reduction of an acceptor molecule *vs*. Fc/Fc^+ in eV) and E_{HOMO} (eV) = $-(E_{\mathrm{i,Fc}}+E_{1/2,\mathrm{D}})$ ($E_{1/2,\mathrm{D}}$ is the redox potential for the first oxidation of a donor molecule *vs*. Fc/Fc^+ in eV).

2. Au Substrate Cleaning and Characterization

Preparation of Au substrates was done by using a double-sided polished phosphorous doped Si-wafer with orientation (100), thickness 525 μm (Siegert Wafer). The wafers were

cleaned with piranha etch, comprising sulfuric acid (96 %) and hydrogen peroxide (30 %), for 10 minutes, followed by an immersion in hydrofluoric acid (1 %) for 20 s (all by Technic), DI H_2O using a quick dump rinse process (custom made wet bench, Stangl), reaching a conductivity of 13 MΩ cm, and dried by heating (ca. 40°C) and application of N_2 in a spin rinser dryer (Semitool). Then, a 500 nm layer of SiO_2 was deposited on the front side of the Si wafer in an oven (Centrotherm) at 990 °C for 170 min. Next, a 10 nm Cr (Cr granulate, size: 0.7 to 3.5 mm, purity ~99,95 %, Evochem) adhesive layer was added (PVD, Leybold UNIVEX 500 with cryopump, Leybold Vacuum), followed by a 100 nm Au (Au granulates, size: 1 to 6 mm, purity ~99.99 %, EMT Edelmetalltechnik) layer. A cross-sectional schematic view of the substrate is shown in Fig. S1. Finally, an AZ 1518 photoresist (Merck) was spin-coated as a protection layer (Delta 20BM, BLE) before dicing the coated wafer into 1 cm^2 pieces using a dicing saw (DAD321, Disco).

Patterned Au substrates were prepared on a double-sided polished phosphorous doped Si-wafer with orientation (100), thickness 525 μm (Siegert Wafer) by utilizing a photolithography process. Initially, the wafer was cleaned with piranha etch, comprising sulfuric acid (96 %) and hydrogen peroxide (30 %), for 10 minutes, followed by an immersion in hydrofluoric acid (1 %) for 20 s (all by Technic), DI H_2O using a quick dump rinse process (custom made wet bench, Stangl), reaching a conductivity of 13 MΩ cm, and dried by heating (ca. 40°C) and application of N_2 in a spin rinser dryer (Semitool). Then, a 500 nm layer of SiO_2 was deposited on the front side of the Si wafer in an oven (Centrotherm) at 990 °C for 170 min. Then, the wafer was coated with hexamethyldisilazane (HMDS) on a vacuum hotplate from BLE. A reverse photoresist (AZ 5214E) was spin coated on the wafer, then soft baked at 110 ºC for 50 s. Then the wafer was exposed to UV light (Maskalinger MA6/BA6, SUSS Micro Tec, with a mercury vapor lamp, Osram) through a mask (antireflective chrome on soda lime, dark field - chrome down - right reading, 5 × 5 × 0.9 $inch^3$, Delta Mask, Netherlands) with our designed

pattern for 0.8 s at an intensity of 9 mW/cm^2. After the light exposure, the wafer was reverse baked at 115 °C for 120 s. Then, the entire wafer was again exposed to light without a mask for 20 s at the same intensity of 9 mW/cm^2, followed by resist development in a spray developer (SUSS Micro Tec) with AZ 726 MIF (Merck). Then, a 10 nm Cr (Cr granulate, size: 0.7 to 3.5 mm, purity ~99,95 %, EVOCHEM) adhesive layer was added (PVD, Leybold UNIVEX 500 with cryopump, Leybold Vacuum), followed by a 100 nm Au (Au granulates, size: 1 to 6 mm, purity ~99.99 %, EMT Edelmetalltechnik) layer. A schematic view of the patterned substrate is shown in Fig. S1. Finally, an AZ 1518 photoresist (Merck) was spin-coated as a protection layer (Delta 20BM, BLE) before dicing the coated wafer into 1 cm^2 pieces using a dicing saw (DAD321, Disco).

Au substrate cleaning and the removal of the AZ1518 resist were done as follows: Au substrates were ultra-sonicated (Elmasonic S15, Elma) in acetone (purity ~100 %, VWR Chemicals) for 30 minutes, repeated 2 times, and then once in ethanol (purity ~100 %, VWR Chemicals). Subsequently, substrates were kept in ethanol for 3 days. After 3 days, substrates underwent another round of ultra-sonication in fresh ethanol, 3 times for 5 minutes each. After two steps of ultra-sonication in acetone and ethanol, respectively, the dried substrates were immersed in RCA solution (Radio Corporation of America) with a volume ratio of 5 : 1 : 1 of H_2O (Purelab Chorus 1, Elga LabWater, 18.2 MΩ cm), NH_3 (Roth, Karlsruhe, 28.0-30.0 %), H_2O_2 (Sigma-Aldrich, ≥30 %) for 1 hour. Then, the Au substrates were 3 times ultra-sonicated in pure H_2O for 5 minutes each, followed by drying under a N_2 stream.

Scanning Electron Microscopy (SEM) was performed with a field emission gun scanning electron microscope (FEG-SEM) SU8220 (Hitachi) with an acceleration voltage of 30.0 kV. The grain size was determined using ImageJ 1.54d/ Java 1.8.0_345 [119] and applying the set scale and the measure function. The distribution was fitted by a Gaussian obtaining the mean

value from the maximum and the error as the standard deviation of the distribution, respectively.

X-ray diffraction (XRD) was performed in the Bragg-Brentano geometry using a D8 DISCOVER diffractometer (Bruker) equipped with Cu Kα radiation, Ni filter, divergence slit (0.1 mm) and a LYNXEYE XE-T detector. Experiments were carried out in a 2θ range of 30 to 90° with a step size of 0.005°. The crystallite size was estimated using the TOPAS program (V6.0, Bruker), taking the integral breadth based L_{Vol} calculation using Lorentzian and Gaussian type component convolutions of the Au(111) peak.

3. RAM-CH$_2$SH Immobilization on Au Substrate and AFM Cantilever Tip

Aliquots of $C_{12}H_{25}SH$ (anhydrous, purity ≥98 %, Sigma-Aldrich) and RAM-CH$_2$SH (TPA-CH$_2$SH (**1**), TTF-CH$_2$SH (**2**), TCAQ-CH$_2$SH (**3**)) and solvents were prepared in a glovebox (Braun Labstar). Prepared aliquots of $C_{12}H_{25}SH$ and solvents were stored at 5 °C and aliquots of RAM-CH$_2$SH were stored at −20 °C. A 0.5 mM RAM-CH$_2$SH solution of the respective molecules was prepared by dissolving $C_{12}H_{25}SH$, TPA-CH$_2$SH (**1**), and TTF-CH$_2$SH (**2**) in toluene (anhydrous, purity ~99.8 %, Thermo Scientific) and TCAQ-CH$_2$SH (**3**) in chloroform (anhydrous, amylene stabilizer, purity >99 %, Sigma Aldrich), respectively. Immobilization of these molecules was done by immersing a cleaned (patterned) Au substrate and NPG-10 (Bruker AFM Probes) cantilevers into 5 ml of the 0.5 mM RAM-CH$_2$SH or $C_{12}H_{25}SH$ solution for 14 hours under dark inert conditions at ca. 22 °C. To retain the immobilization under dark and inert conditions, a brown glass desiccator (WBJLG, China) was used, wherein a 20 ml beaker filled with toluene was placed. Then, the (silicone grease) sealed brown glass desiccator was flooded with N_2 gas. After incubation, unbound molecules were removed by rinsing the Au substrates in fresh toluene and chloroform, respectively, for 3 times, followed by drying under a N_2 stream. The dried substrates were stored in N_2 in a sealed brown glass desiccator.

4. Characterization of RAM-CH_2SH Immobilization on Au Substrate

X-ray photoelectron spectroscopy (XPS) was done using a PHI 5000 VersaProbe III (ULVAC-PHI) with the following acquisition parameters: Al monochromatic probe beam with an energy of 1486.6 eV, a beam power of 25.4 W and a beam diameter of 100 μm. The photoelectron takeoff angle for survey and detail scans was 18.5°, to probe approximately ca. 3 nm of the surface depth. The analyzer pass energy for survey and detail scans were 224 eV and 55 eV, respectively, which delivered an energy resolution of 0.8 and 0.05 eV, respectively. Measured spectra were analyzed using CasaXPS software (Version 2.3.26).[120] For the analysis, all spectra were referenced with Au $4f_{7/2}$ (binding energy of 83.99 eV). Shirley and Lorentzian-Gaussian algorithms were used to correct for the background and for peak deconvolutions and fitting, respectively. Peak deconvolutions were done with the application of the integrated peak area and splitting energy constraints for respective orbitals. For S 2p and Au 4f doublets, 2:1 and 4:3 peak area ratios and 1.2 eV and 3.68 eV splitting energies were applied, respectively.

AFM imaging was done using a Cypher ES (Asylum Research, an Oxford Instruments Company) in the piezo-driven intermittent-contact mode (AC mode) in air with Scout 70 RAl (NuNano) and OMCL-AC240TS (Olympus) cantilevers (nominal spring constant: 2 N/m, resonant frequency: 70 kHz and tip radius: ca. 5 nm) at a temperature of ca. 20 °C and ca. 37% relative humidity (RH). The following parameters were applied: scan size: (500 × 500) nm^2, pixels: 512^2, scan rate: 2.44 Hz and scan angle: 0° (parallel to the long cantilever axis). The substrates were mounted on steel mounting disks with the help of Ag paste (Ted Pella).

AFM-based scratching of the RAM-functionalized CEAM substrates was performed on a Cypher ES (Asylum Research, an Oxford Instruments Company) in the piezo-driven intermittent-contact mode (AC mode) in air with OMCL-AC240TS (Olympus) cantilevers (nominal spring contact: 2 N/m, resonant frequency: 70 kHz and tip radius: ca. 5 nm) at a

temperature of ca. 22 °C and ca. 37% RH. The following parameters were applied: scan size: (10 × 10) μm^2 and (2 × 2) μm^2, (0.5 × 0.5) μm^2, pixels: 512^2, scan rate: 2.44 Hz and scan angle: ca. 0° (parallel to the long cantilever axis). The substrates were mounted on steel mounting disks with the help of Ag paste (Ted Pella). Scratches were introduced in a region covering the border between Au stripes and the SiO_2 regions via contact mode imaging with HQ:CSC17/Al BS (μmash, inverted optical level sensitivity: ca. 160-200 nm/V, spring constant: ca. 360-400 pN/nm and tip radius: < 8 nm) with the following parameters: scan size: (5 × 5) μm^2, pixels: 256^2, scan rate: 4.88 Hz and scan angle: ca. 0° (parallel to the long cantilever axis), setpoint force: 350 to 380 nN. For each scratching process ten consecutive contact mode images were taken to ensure removal of surface bound RAMs.

All AFM images were analyzed using Gwyddion Free SPM analysis software, version 2.62.[121] For analysis, the AFM images (zSensorRetrace) were processed using the following functions: mean plane subtraction, line median matching (median of differences), correction of horizontal scars and shifting the data values to zero (fix zero function). A linear color scale, gwyddion.net, was used for the presentation of the images. Finally, the root-mean-square (RMS) roughness was determined using the statistical quantities tool.

Static contact angle measurements were done in OCA 15EC (Dataphysics Instruments). For the data acquisition, a 0.2 μl H_2O drop (Purelab Chorus 1, Elga LabWater, 18.2 MΩ cm) was placed on five different spots of the freshly functionalized 1 cm^2 Au substrates at ca. 20 °C and ca. 37% RH. SCA20 software (Dataphysics Instruments) was used for optical contact angle (OCA) data acquisition and determination of the static contact angle at the triple-point of the solid-liquid-gas interface. The mean of the left and right static contact angles was taken at each of the five different spots to determine the mean value and standard deviation as a measure for the error, respectively.

CV on substrates. The electrochemical properties of the electrodes were measured at ambient temperature using a VSP electrochemical workstation (BioLogic). A conventional three-compartment cell was employed throughout the experiments. *n*-Bu_4NPF_6 (Sigma Aldrich) was dried at 110 °C and stored in a desiccator until used as the supporting electrolyte in anhydrous acetonitrile (Sigma Aldrich). Prior to each experiment, the working solution was purged with N_2 for 10 minutes, and a N_2 gas blanket was maintained over the solution during measurements. The counter electrode, a large-area Pt mesh, was separated from the electrolytic solution by a sintered glass frit. The working Au electrodes were mounted on a glassy carbon holder, exposing a final area of 1 × 0.5 cm^2. An Ag wire immersed in a solution of $AgNO_3$ (Sigma Aldrich, 0.1 M) and *n*-Bu_4NPF_6 (Sigma Aldrich, 0.1 M) in acetonitrile served as the pseudo-reference electrode. All potentials are reported relative to the Fc/Fc^+ redox couple (Fc form Sigma Aldrich), used as an internal standard. The redox potential was estimated as the average between the cathodic and anodic peak potential ($E^0 \approx E_{1/2} = (E_{p,c}+E_{p,a})/2$).

5. Density Functional Theory Simulations

Atomic structures were set up analyzed using Atomic Simulation Environment (ASE).[122] The electronic structure was described within Density Functional Theory (DFT) as implemented in GPAW,[123,124] which employs the Projector Augmented-Wave (PAW) method.[125] We approximate the exchange-correlation energy using the Perdew-Burke-Ernzerhof (PBE) [126] functional, as devised in the generalized gradient approximation (GGA), incorporating van der Waals (vdW) corrections to account for dispersion interactions.[127] The Brillouin zone integrations were conducted using the Monkhorst-Pack scheme implemented in GPAW. The smooth part of the Kohn-Sham wave-functions is represented on real space grids with a grid spacing of 0.2 Å unless stated otherwise, while the electron density is represented

with a half grid spacing, i.e., 0.1 Å. The Structures were considered to be relaxed when all forces were lower than the convergence threshold of f_{max} = 0.02 eV/ Å.

Molecular structures were described within Dirichlet boundary conditions, where all atoms were ensured to reside at a distance of at least 4 Å apart from the boundary of the simulation box. Solvent effects were described by a polarizable continuum model [57] with a relative permittivity of $\varepsilon_r = 8.93$ representing the solvent CH_2Cl_2 (DCM) used in the experiment.

The Au surface used in our simulations was a FCC(111) surface with a lattice parameter of 4.08 Å.[122] Two Au(111) surface supercells were employed in the majority of the simulations, unless stated otherwise. The first supercell features a 4 × 4 configuration, containing 16 Au atoms per layer, with a total of 48 Au atoms across three layers, covering an area of 1.154 nm × 1.0 nm (1.154 nm^2). This configuration was used for simulations involving SCH_3 and RAM-CH_2SH standing on the Au surface. The second supercell, used for simulations of RAM-CH_2SH lying flat on the Au surface, has a 6 × 6 configuration, containing 36 Au atoms per layer, with a total of 108 Au atoms across three layers, covering an area of 1.731 nm × 1.5 nm (2.597 nm^2). The Brillouin zone in periodic calculations is represented by a 3 × 3 × 1 k-point Monkhorst-Pack mesh unless stated otherwise.[128] The Au atoms in the upper two atomic layers were permitted to relax, while those in the bottom layer were held fixed to simulate a bulk-like termination. A finer grid spacing *h* of 0.18 Å was chosen for the real-space calculations. This threshold was chosen to ensure the forces acting on each atom were minimized, ensuring structural stability and accuracy.

For our XPS analysis, we implemented the offset-corrected Δ-Kohn-Sham scheme as introduced by Walter, Moseler, and Pastewka.[62] This scheme is specifically designed to predict XPS spectra on an absolute energy scale. The offset-corrected Δ-Kohn-Sham scheme involves calculating the core-electron binding energies using DFT and then applying an empirical correction to offset systematic errors. By introducing an offset correction based on

reference data, the method significantly improves the accuracy of the predicted absolute binding energies. First, we calculated the core-level shifts within the final state description as the energy difference between the ground state electronic structure and the electronic structure including a core-hole within the frozen core as well as an additional electron in the valence. As we have a metallic part (the Au support) with vanishing band-gap, this procedure leads to the predicted core-hole energy relative to the electrically connected spectrometer level. The extra electron also neutralizes the system and avoids spurious self-interactions by the charges in periodic unit cells.[129–131] Then, we applied the offset correction to these shifts to obtain the absolute binding energies corresponding to the S $2p_{3/2}$ state. To calculate the S $2p_{1/2}$ contribution arising from spin-orbit coupling, we added the same contribution shifted by the experimental spin-orbit splitting of 1.2 eV [132] to lower energy and with half the weight. This approach ensured high accuracy in our XPS spectral predictions and made our results directly comparable to experimental data. In this study, a full width at half maximum (FWHM) value of 0.94 eV was used for the Gaussian peaks to ensure consistent peak resolution across all measurements in the XPS spectra. This parameter choice allowed for reliable comparisons of binding energy distributions across different samples.

6. Contact Electrification At the Micrometer Scale (CEAM) Assay of RAM-CH_2SH Immobilized Au Substrates

The **CEAM** assay was performed in 3 steps on a Cypher ES (Asylum Research, an Oxford Instruments Company) under N_2 atmosphere (22°C, 14% RH, see below) using the above-described patterned Au/SiO_2 substrate consisting of set of two rectangular Au stripes (dimensions: $(10 \times 30)\,\mu m^2$), wherein the stripe labelled as contact stripe (CS) was used for force spectroscopy-based contact-separation experiment and the other stripe served as a reference stripe (RS). The steps are shown schematically in Fig. 1. In the first step, the contact

potential difference of the CS and the RS was measured via Kelvin probe force microscopy (KPFM, nap mode). OMCL-AC240TM (Olympus) cantilevers were used for KPFM measurements. The KPFM cantilever tip consisted of a Si probe with a conductive 20 nm Pt layer and a titanium (Ti) interfacial layer underneath. The nominal spring constant, resonant frequency and tip radius of the cantilever were 2 N/m, 70 kHz and 15 nm, respectively. The inverted optical level sensitivity (InvOLS) and the spring constant were determined by the Sader method.[133] The KPFM measurement parameters were as follows: scan size: $(30 \times 30)\ \mu m^2$, scan rate: 0.75 Hz, pixels: 512^2, nap height: ca. 20 nm, bias voltage: ca. 3 V. They were kept constant for all measurement, unless stated differently. In the second step, the contact-separation of a (RAM-functionalized) Au AFM cantilever tip and a patterned Au substrate was performed in force spectroscopy mode. For the force spectroscopy mode, NPG-10 (Bruker AFM Probes) cantilevers of type A were used. These cantilevers were made of Si_3N_4 with a (45 ± 5) nm of tip side Au coating and back side reflective Ti/Au coating and had the following nominal spring constant, resonant frequency, tip radius, length and width: 0.35 N/m, 65 kHz, 30 nm, 120 μm and 25 μm. For each new force spectroscopy experiment, the cantilever was calibrated to get the InvOLS and the spring constant using the Sader method.[133] 20×20 force extension curves were taken in a grid-like fashion (force map) across an area of $(5 \times 5)\ \mu m^2$. The contact-separation parameters were the following: trigger force: ca. 10 nN, loading (approach/ indentation) velocity: 1.0 $\mu m\ s^{-1}$, unloading (retraction) velocity: 50.0 $\mu m\ s^{-1}$, dwell time toward the surface: 5.0 s and sampling rate: 16.66 kHz. In the third step, the contact potential difference of the CS and RS were measured via KPFM (nap mode).

Humidity adjustment. The humidity of the AFM was decreased with a N_2 purging system using the gas manifold system of the Cypher ES. The two gas lines attached to the sample cell were routed via tubings and fittings to the bottom of the Cypher ES scanner. One of the lines branches off to a pressure sensor and then passes through a manually or digitally controlled

valve. The maximum operation pressure of the scanner valve was 200 mbar. In order to regulate the N_2 flow safely, a low-pressure gas regulator was installed to the main N_2 gas stream.

The humidity was measured using a humidity sensor (HIH-4000-002, Honeywell), a 5 V power supply (V_{supply}) and a multimeter (VC140, VOLTCRAFT) for measuring the output voltage (V_{out}) upon change of the N_2 purging pressure. With the measured V_{out}, the humidity was calculated using the Eqns. S1 and S2, according to the manual of the humidity sensor. Taking the humidity h_{sensor} measured with the humidity sensor:

$$h_{sensor} = \frac{\frac{V_{out}}{V_{supply}} - 0.16}{0.0062} \quad \text{(S1)}$$

and correcting for the temperature T (22 °C), the true humidity h_{true} can be obtained by:

$$h_{true} = \frac{h_{sensor}}{1.0546 - 0.00216 \cdot T} \quad \text{(S2)}$$

At a pressure of 100 mbar in the sample cell, ca. 14% RH was achieved. For the CEAM assay, the sample cell was first purged with N_2 at 100 mbar pressure for the 15 min, then the pressure was reduced to the 10 mbar for the experiments to prevent any increase of the Cypher ES noise floor.

7. Data Evaluation for CEAM Assay

The obtained **KPFM images** were evaluated using Gwyddion Free SPM analysis software, version 2.62.[121] For analysis, the SiO_2 area in trace images was first masked (with the help of the mask editor tool) to correct for slopes in the SiO_2 background. Then, the whole image was processed in the following order: mean plane subtraction, line median matching (median of differences) and correction of horizontal scars. The presented KPFM images show absolute potential values. A linear spectral color scale was used for the presentation of the image. The contact potential difference V_{CPD} distributions of CS and RS (within the areas indicated by the

broken lines in Fig. 3 b and c), before and after the contact-separation experiment, were computed by normalized distributions $\varrho(V_{\mathrm{CPD}})$, according to:

$$\int_{-\infty}^{\infty} \rho(V_{\mathrm{CPD}}) dV_{\mathrm{CPD}} = 1 \tag{S3}$$

The mean values $\bar{V}_{\mathrm{CPD,CS}}$ and $\bar{V}_{\mathrm{CPD,RS}}$ obtained from the maximum of the respective distributions $\varrho(V_{\mathrm{CPD}})$ were used to determine the mean contact potential difference between the CS and the RS, for both before and after the contact-separation process:

$$\bar{V}_{\mathrm{CPD,before\ contact}} = \left(\bar{V}_{\mathrm{CPD,CS}} - \bar{V}_{\mathrm{CPD,RS}}\right)_{\mathrm{before\ contact}} \tag{S4a}$$

$$\bar{V}_{\mathrm{CPD,after\ contact}} = \left(\bar{V}_{\mathrm{CPD,CS}} - \bar{V}_{\mathrm{CPD,RS}}\right)_{\mathrm{after\ contact}} \tag{S4b}$$

Thus, the mean surface potential change due to the contact-separation process is given by:

$$\overline{\Delta V}_{\mathrm{CPD}} = \bar{V}_{\mathrm{CPD,after\ contact}} - \bar{V}_{\mathrm{CPD,before\ contact}} \tag{S5}$$

Error values were obtained by quadratic error propagation based on the standard deviations of $\varrho(V_{\mathrm{CPD}})$ for CS and RS, either before and after the contact-separation process:

$$sd(\Delta V_{\mathrm{CPD}}) = \left[sd_{\rho}\left(V_{\mathrm{CPD,CS,after\ contact}}\right)^{2} + sd_{\rho}\left(V_{\mathrm{CPD,RS,after\ contact}}\right)^{2} + sd_{\rho}\left(V_{\mathrm{CPD,CS,before\ contact}}\right)^{2} + sd_{\rho}\left(V_{\mathrm{CPD,RS,before\ contact}}\right)^{2}\right]^{1/2} \tag{S6}$$

Force spectroscopy data were evaluated with a self-written program based on Igor Pro. The evaluation of each force-extension curve was done as given by Kolberg *et al.* :[134] after drift correction, the maximum force of the adhesion peak (adhesion force, F_{adhesion}) was obtained from the retraction portion of the force-extension curve. All mean and error (standard deviation) values of the adhesion forces were obtained from 20 × 20 force maps across an area of (5 × 5) μm^2, comprising 400 force-extension curves each (Fig. S51 a and c and Table S5). The respective contact forces were determined from the force values at the beginning of the retraction curves (Fig. S51 b and Table S5).

For the **contact area** and **pressure determination** between AFM cantilever tip and substrate, the Derjaguin-Muller-Toporov (DMT) model [135] was used, considering the plane-

sphere geometry (neglecting the finite substrate roughness [136]), where plane and sphere refer to the Au substrate surface and the Au tip is idealized by a sphere having the nominal tip radius of the NPG10 cantilever (r_{tip} = 30 nm), respectively. The following equations were used to estimate the contact radius r_{DMT}, the contact area a_{DMT}, and the contact pressure p_{DMT}:

$$r_{DMT} = \left[\frac{3r_{tip}(F_{adhesion}+F_{contact})}{4E_{tot}}\right]^{\frac{1}{3}} \quad (S7)$$

$$a_{DMT} = \pi r_{DMT}^2 \quad (S8)$$

$$p_{DMT} = \frac{F_{adhesion}+F_{contact}}{a_{DMT}} \quad (S9)$$

F is the load, i.e., contact force ($F_{contact}$), applied by the AFM cantilever tip (ca. 10 nN, see Table S5). E_{tot} is the total elastic modulus computed by:

$$\frac{1}{E_{tot}} = \left(\frac{1-v_1^2}{E_1} + \frac{1-v_2^2}{E_2}\right) \quad (S10)$$

Here, ν and E represent the Poisson's ratio and the elastic modulus. For Au, those are $\nu = 0.42$ and $E = 74$ GPa [137] leading to $E_{tot} = 45$ GPa. All mean values of the contact area (a_{DMT}) and the contact pressure (p_{DMT}) are listed in Table S5. The errors were determined by using the standard deviations and quadratic error propagation.

The **amount of transferred charge** was determined by obtaining the mean surface-charge-density change $\overline{\Delta\sigma}$ upon contacting based on the mean surface potential change $\overline{\Delta V}_{CPD}$. The surface-charge-density σ is defined by a charge Q and an area A as follows:

$$\sigma = \frac{Q}{A} \quad (S11)$$

For KPFM often the analogy to the plate-capacitor model is used,[68,138,139] which, however, might underestimate the real surface-charge-density, as discussed by Pertl *et al.* [69] and thus serves rather as a lower limit for a surface-charge-density estimation. Using the vacuum dielectric constant $\varepsilon_0 = 8.85 \cdot 10^{-12} \frac{\mathrm{A\,s}}{\mathrm{V\,m}}$, the relative dielectric constant of the dielectric medium (SiO_2, as shown in Fig. S1 c) $\varepsilon_{SiO_2} = 4.5$,[140] the thickness of the dielectric medium

(SiO_2) of $d = 500$ nm, neglecting the ca. 10 nm thick Cr layer (see Fig. S1 for the cross-section), and the mean surface potential change $\overline{\Delta V}_{\mathrm{CPD}}$, the mean surface-charge-density change $\overline{\Delta\sigma}$ can be obtained by:

$$\overline{\Delta\sigma} = \frac{\varepsilon_{\mathrm{SiO_2}} \cdot \varepsilon_0 \cdot \overline{\Delta V}_{\mathrm{CPD}}}{d} \qquad \text{(S12)}$$

B. Supporting Data

1. Experimental Au Substrate

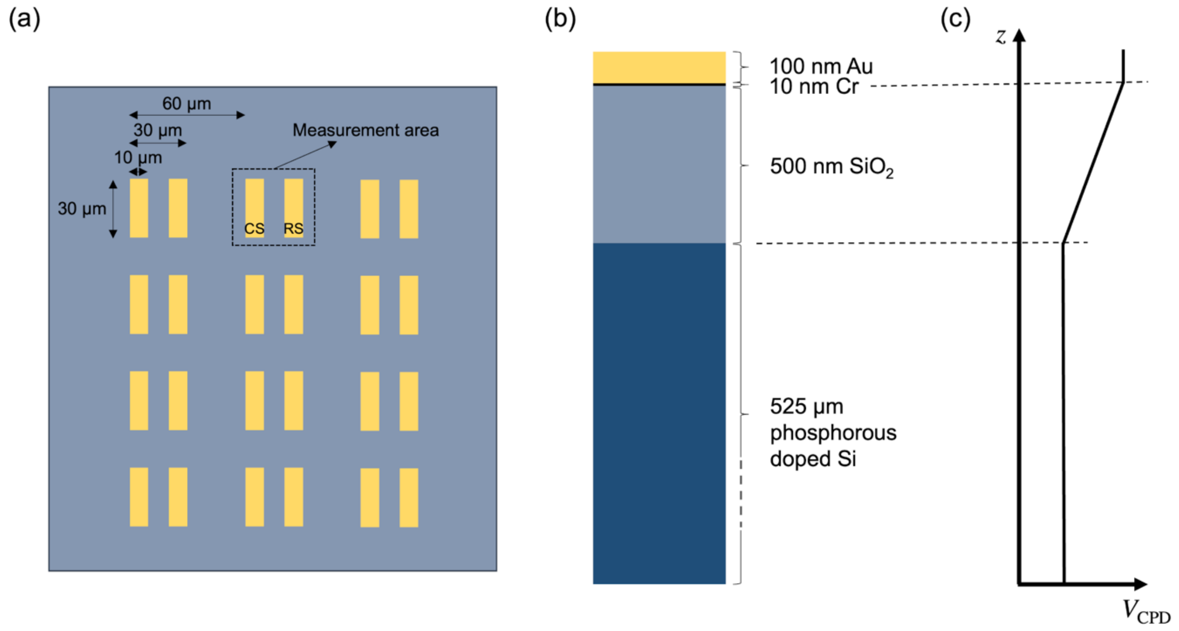

Figure S1. (a) Layout of CEAM substrate. 12 sets of Au stripe pairs (represented as CS and RS), each one with lateral dimensions of $(10 \times 30)\,\mu m^2$. The background shown in blue is SiO_2. One set of Au stripes was used for the each CEAM experiment. (b) Cross-sectional layer structure of the CEAM substrate, wherein the Au stripes consist of a Cr and Au layer on top of the SiO_2 layer, isolating the Au stripes from each other. Fully Au-coated substrates used in this study have the same cross-sectional layer structure as shown in b. (c) Contact potential difference V_{CPD} against KPFM cantilever tip along the cross-sectional layer structure for either CS or RS. The phosphorous doped Si is connected to ground. V_{CPD} drops over the dielectric SiO_2 layer. The mean surface-charge-density change $\overline{\Delta\sigma}$ is obtained via eqn. S12 neglecting the 10 nm thick Cr layer.

2. Synthesis and Characterization of CH_2SH-substituted RAMs

Synthetic Overview

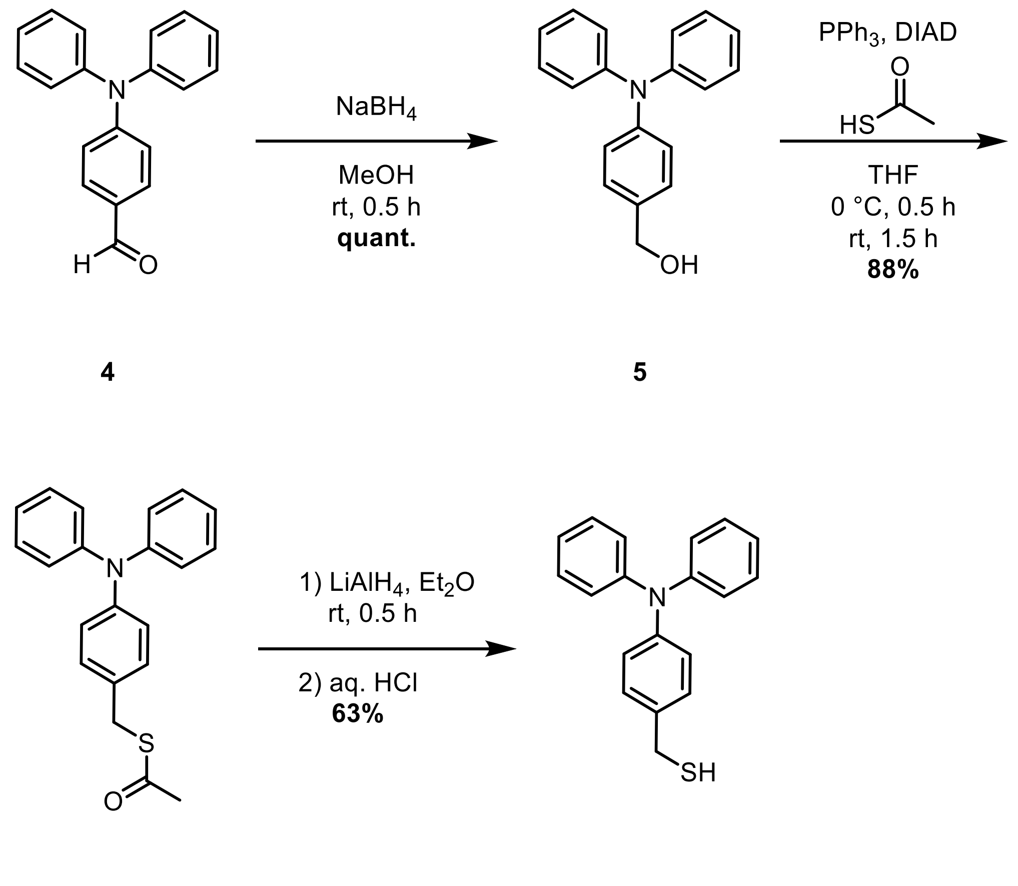

Scheme S1. Synthetic approach to TPA-CH_2SH (**1**).

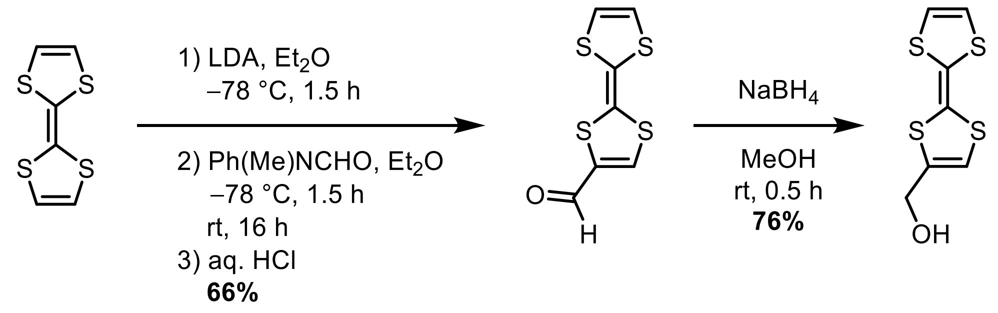

7 8 9

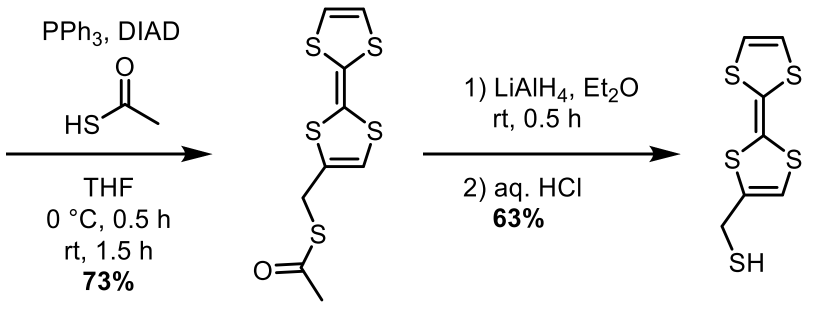

10 2

Scheme S2. Synthetic approach to TTF-CH_2SH (**2**).

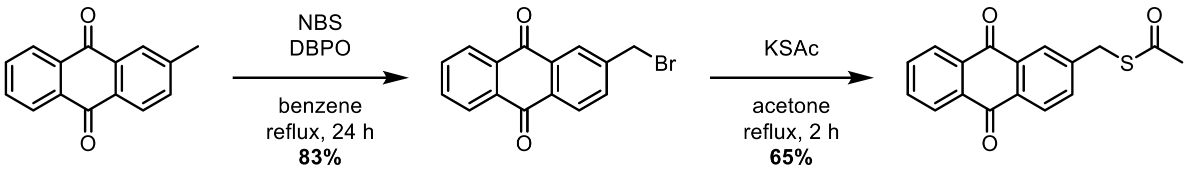

11 12 13

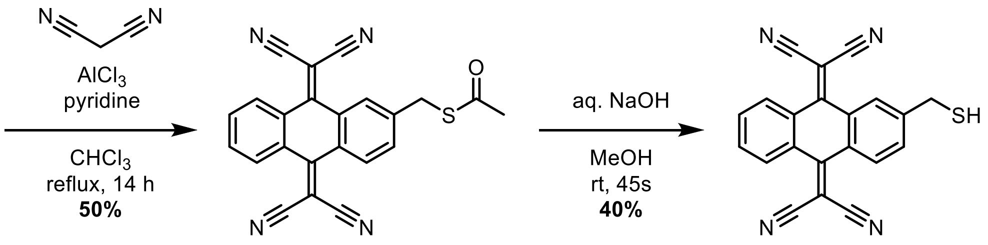

14 3

Scheme S3. Synthetic approach to TCAQ-CH_2SH (**3**).

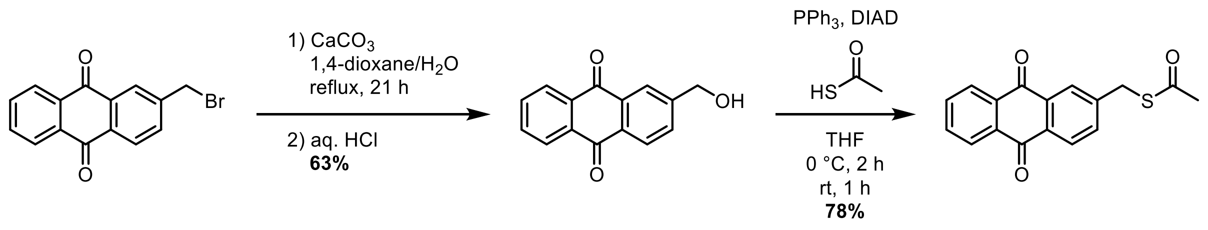

12 **15** **13**

Scheme S4. Alternative approach to 2-(acetylsulfanylmethyl)-anthraquinone (**13**).

Synthetic Procedures

4-Hydroxymethyltriphenylamine (5)

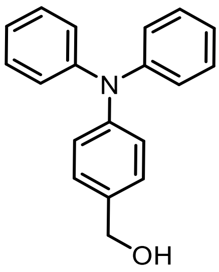

The synthesis was performed following a procedure by Garín *et al.* .[51] 4-Formyltriphenylamine (**4**, 501 mg, 1.83 mmol) was dissolved in degassed MeOH (27 ml). Then, $NaBH_4$ (91 mg, 2.4 mmol, 1.3 eq.) was added in one portion and the yellow suspension was stirred at 24 °C for 30 min under argon. At the end of the reaction time a colorless solution was obtained, indicating the end of the reaction. CH_2Cl_2 (30 ml) was added, and the solution was poured into H_2O (30 ml), followed by separation of the layers. The aqueous (aq.) layer was extracted with CH_2Cl_2 (3 × 20 ml) and the combined organic extracts were washed with brine (50 ml), dried over $MgSO_4$ and the solvent was removed *in vacuo*. 4-Hydroxymethyltriphenylamine (**5**, 504 mg, 1.83 mmol, quant.) was obtained without any further purification as a beige solid.

***R**$_f$* 0.59 (CH_2Cl_2); **^{1}H NMR** (400 MHz, CD_2Cl_2) δ = 7.30–7.19 (m, 6H), 7.12–6.94 (m, 8H), 4.60 (s, 2H), 1.77 (br. S, 1H); **^{13}C NMR** (100 MHz, CD_2Cl_2) δ = 148.2, 147.7, 136.0, 129.6, 128.5, 124.5, 124.4, 123.2, 65.2; **HRMS** (pos. APCI): *m/z* calcd. for $C_{19}H_{18}NO^+$ 276.1388 $[M+H]^+$, found 276.1379.

4-(Acetylsulfanylmethyl)triphenylamine (6)

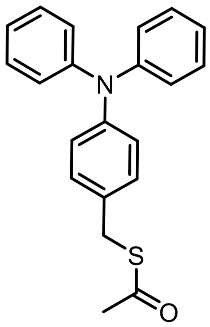

The synthesis was performed following a procedure by Yuge *et al.* .[52] A flame-dried Schlenk tube was charged with triphenylphosphine (381 mg, 1.45 mmol, 2.00 eq.), and anhydrous, degassed THF (4.9 ml) was added. The solution was cooled to 0 °C followed by dropwise addition of diisopropyl azodicarboxylate (0.29 ml, 0.29 g, 1.5 mmol, 2.0 eq.). A white precipitate was formed after 3 min, and the mixture was stirred at 0 °C for 30 min. Afterwards, a solution of 4-hydroxymethyl-TPA (**5**, 0.20 g, 0.73 mmol) and thioacetic acid (0.13 ml, 0.14 g, 1.8 mmol, 2.5 eq.) in anhydrous, degassed THF (1.8 ml) was slowly added over 5 min, and the reaction mixture was stirred at 0 °C for 30 min. After this time, the reaction was stirred at 24 °C for another 1.5 h, before the solvent was removed under reduced pressure. The residue was purified by column chromatography (silica gel, petroleum ether/CH_2Cl_2: 7/3 to 1/1) to yield 4-(acetylsulfanylmethyl)triphenylamine (**6**, 213 mg, 0.667 mmol, 88 %) as a colorless oil.

$\boldsymbol{R_f}$ 0.63 (petroleum ether/CH_2Cl_2: 7/3); **^{1}H NMR** (400 MHz, CD_2Cl_2) δ = 7.30–7.22 (m, 4H), 7.20–7.14 (m, 2H), 7.10–6.96 (m, 8H), 4.10 (s, 2H), 2.36 (s, 3H); **^{13}C NMR** (100 MHz, CD_2Cl_2) δ = 195.3, 148.1, 147.4, 132.2, 130.1, 129.6, 124.6, 124.2, 123.3, 33.4, 30.6; **HRMS** (pos. MALDI): *m/z* calcd. for $C_{21}H_{19}NOS^{\bullet+}$ 333.1187 $[M]^{\bullet+}$, found 333.1180.

4-(Mercaptomethyl)triphenylamine (1)

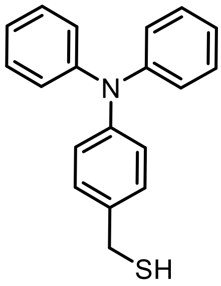

The synthesis was performed following a procedure by Yuge *et al.* .[52] $LiAlH_4$ (21.0 mg, 0.55 mmol, 1.28 eq.) was suspended in anhydrous, degassed Et_2O (7.0 ml) and stirred at 24 °C for 5 min. Then, a solution of thioester (**6**, 0.14 g, 0.43 mmol) in anhydrous, degassed Et_2O (10 ml) was added dropwise at 24 °C and the mixture was stirred at 24 °C for 30 min. Afterwards, the reaction was quenched by adding aq. HCl (1 M, 15 ml) and the layers were separated. The aq. layer was extracted with Et_2O (3 × 10 ml) and the combined organic extracts were washed with brine, dried over $MgSO_4$ and the solvent was removed *in vacuo*. The residue was purified by column chromatography (silica gel, petroleum ether/CH_2Cl_2: 7/3) to yield the 4-(mercaptomethyl)triphenylamine (**1**, 79 mg, 0.27 mmol, 63 %) as a yellow solid.

$\boldsymbol{R_f}$ 0.48 (petroleum ether/CH_2Cl_2: 7/3); **^{1}H NMR** (400 MHz, CD_2Cl_2) δ = 7.29–7.18 (m, 6H), 7.11–6.98 (m, 8H), 3.72 (d, J = 7.4 Hz, 2H), 1.84 (t, J = 7.5 Hz, 1H); **^{13}C NMR** (100 MHz, CD_2Cl_2) δ = 148.2, 147.1, 136.0, 129.6, 129.3, 124.6, 124.4, 123.2, 28.8; **HRMS** (pos. MALDI): *m/z* calcd. for $C_{19}H_{17}NS^{•+}$ 291.1082 $[M]^{•+}$, found 291.1078.

Formyltetrathiafulvalene (8)

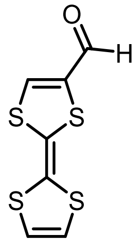

The synthesis was performed following a procedure by Garín *et al.* .[51] Tetrathiafulvalene (**7**, 109 mg, 0.528 mmol) was dissolved in anhydrous Et_2O (6 ml) and cooled to −78 °C. Lithium diisopropylamide (1.33 M in Et_2O/hexane, 0.44 ml, 0.59 mmol, 1.1 eq) was added dropwise. The mixture was stirred at −78 °C for 1.5 h resulting in a yellow suspension. *N*-methylformanilide (0.23 ml, 1.9 mmol, 3.5 eq) in anhydrous Et_2O (0.9 ml) was added. The mixture was stirred at −78 °C for 1.5 h and was allowed to warm to 24 °C over 16 h. The mixture was poured into aq. HCl (0.75 M, 19 ml) resulting in a dark red solution. The phases were separated and the aq. phase was extracted with CH_2Cl_2 (5 × 15 ml). The combined organic extracts were washed with H_2O (2 × 15 ml) and brine (20 ml), dried over $MgSO_4$, and the solvent was removed under reduced pressure. Column chromatography (SiO_2, cyclohexane/CH_2Cl_2: 8/2 to 7/3) yielded formyltetrathiafulvalene (**8**, 81 mg, 0.35 mmol, 66 %) as a dark red solid.

R_f 0.50 (cyclohexane/EtOAc: 3/1); **^{1}H NMR** ($CDCl_3$, 300 MHz, 300 K): δ 9.48 (s, 1H), 7.42 (s, 1H), 6.36 (d, *J* = 6.5 Hz, 1H), 6.32 (d, *J* = 6.5 Hz, 1H); **^{13}C NMR** (151 MHz, $CDCl_3$): δ 179.8, 141.7, 140.0, 119.5, 118.9, 116.1, 105.7.

Hydroxymethyltetrathiafulvalene (9)

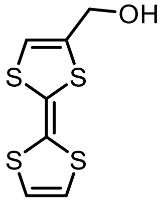

The synthesis was performed following a procedure by Garín *et al.* .[51] Formyltetrathiafulvalene (**8**, 184 mg, 0.748 mmol) was dissolved in degassed MeOH (7.5 ml), and $NaBH_4$ (32 mg, 0.85 mmol, 1.1 eq.) was added. The red solution turned yellow after approximately 2 min indicating the end of the reaction. To ensure complete consumption of the starting material, the mixture was stirred at 24 °C for another 30 min. CH_2Cl_2 (20 ml) was added, and the mixture was poured into H_2O (20 ml). The phases were separated and the aq. phase was extracted with CH_2Cl_2 (2 × 20 ml). The combined organic extracts were washed with brine (20 ml), dried over $MgSO_4$, and the solvent was removed under reduced pressure. Column chromatography (SiO_2, CH_2Cl_2) yielded hydroxymethyltetrathiafulvalene (**9**, 132 mg, 0.563 mmol, 76 %) as a yellow solid.

$\boldsymbol{R_f}$ 0.33 (CH_2Cl_2); **^{1}H NMR** ($CDCl_3$, 500 MHz, 300 K): δ 6.31 (s, 2H), 6.23 (s, 1H), 4.40 (d, J = 5.8 Hz, 2H), 1.78 (d, J = 6.0 Hz, 1H); **^{13}C NMR** ($CDCl_3$, 126 MHz, 300 K): δ 136.8, 119.2, 119.2, 115.6, 111.8, 109.6, 60.9; **HRMS** (pos. APCI): *m/z* calcd. for $C_7H_6OS_4$ 234.9374 $[M+H]^+$, found 234.9374.

Acetylsulfanylmethyltetrathiafulvalene (10)

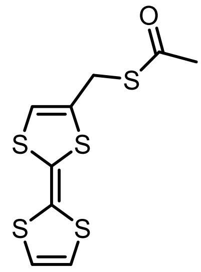

The synthesis was performed following a procedure by Yuge *et al.* .[52] Diisopropyl azodicarboxylate (201 mg, 0.974 mmol, 1.90 eq.) was added dropwise to a solution of triphenylphosphine (272 mg, 1.03 mmol, 2.01 eq.) in anhydrous, degassed THF (2.5 ml) at 0 °C. The mixture was stirred for 30 min at 0 °C and a white precipitate formed. A solution of hydroxymethyltetrathiafulvalene (**9**, 120 mg, 0.512 mmol) and thioacetic acid (102 mg, 1.29 mmol, 2.51 eq.) in anhydrous, degassed THF (1.25 ml) at 0 °C was added dropwise within 5 min. The mixture was stirred at 0 °C for 30 min and for another 1.5 h at 24 °C. Subsequently, it was concentrated under reduced pressure. Column chromatography (SiO_2, $d = 2$ cm, $h = 15$ cm; CH_2Cl_2) yielded acetylsulfanylmethyltetrathiafulvalene (**10**, 110 mg, 0.375 mmol, 73 %) as an orange solid.[1]

$\boldsymbol{R_f}$ 0.55 (cyclohexane/CH_2Cl_2: 1/1); **^{1}H NMR** ($CDCl_3$, 300 MHz, 300 K): δ 6.30 (s, 2H), 6.19 (t, J = 1.1 Hz, 1H), 3.86 (d, J = 1.1 Hz, 2H), 2.37 (s, 3H); **^{13}C NMR** ($CDCl_3$, 126 MHz, 300 K): δ 194.3, 132.4, 119.2, 119.1, 116.8, 111.6, 109.7, 30.6, 29.0; **HRMS** (pos. ESI): *m*/*z* calcd. for $C_9H_8OS_5$ 291.9173 $[M]^+$, found 291.9170.

[1] The product contained 5.5mol % of $SP(Ph)_3$. The yield was corrected according to the ratio of the ^{1}H NMR-signals at 7.54–7.41 ppm (m, 9 H) for $SP(Ph)_3$ and 6.30 ppm (s, 2H) for acetylsulfanylmethyltetrathiafulvalene **10**. However, the compound was used without further purification.

Mercaptomethyl tetrathiafulvalene (2)

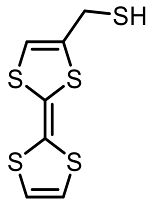

The synthesis was performed following a procedure by Yuge *et al.* .[52] A solution of acetylsulfanylmethyltetrathiafulvalene (**10**, 55 mg, 0.18 mmol) in anhydrous, degassed Et_2O (5 ml) was added dropwise to a suspension of $LiAlH_4$ (8.5 mg, 0.22 mmol, 1.2 eq.) in anhydrous, degassed Et_2O (3 ml). The resulting mixture was stirred at 24 °C for 30 min. Subsequently, aq. HCl (1 M, 5 ml) was added. The phases were separated, and the organic phase was concentrated under reduced pressure. Column chromatography (SiO_2, $d = 2$ cm, $h = 20$ cm; cyclohexane/CH_2Cl_2: 4/1) yielded mercaptomethyl tetrathiafulvale (**2**, 28 mg, 0.11 mmol, 63 %) as a red oil.[2]

R R_f 0.60 (cyclohexane/CH_2Cl_2: 4/1); **^{1}H NMR** ($CDCl_3$, 300 MHz, 300 K): δ 6.31 (s, 2H), 6.17 (t, J = 1.2Hz, 1H), 3.50 (dd, J = 7.9, 1.2Hz, 2H), 6.17 (t, J = 8.0 Hz, 1H); **HRMS** (pos. ESI): m/z calcd. for $C_7H_6S_4$ 249.9068 $[M]^+$, found 249.9065.

[2] The product contained 1.5 % of Et_2O. The yield was corrected according to the ratio of the ^{1}H NMR-signals at 1.21 ppm (t, 6 H) for Et_2O and 6.31 ppm (s, 2H) for TTF-CH_2SH (**2**).

2-(Bromomethyl)anthraquinone (12)

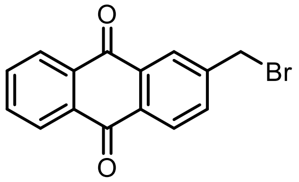

The synthesis was performed following a procedure by Hou *et al.* .[53] 2-Methyl-anthraquinone (**11**, 10.0 g, 44.6 mmol) was dissolved in benzene (500 ml). NBS (10.2 g, 56.2 mmol, 1.26 eq.) and dibenzoylperoxide (75wt % in H_2O, 1.48 g, 4.34 mmol, 9.70 mol %), were added, and the mixture was stirred at reflux for 24h. After addition of H_2O (250 ml), the phases were separated. The organic phase was washed with H_2O (2 × 250 ml) and brine (250 ml), dried over $MgSO_4$ and the solvent was removed under reduced pressure. Recrystallisation from 2-propanol (1 l) afforded 2-(bromomethyl)anthraquinone (**12**, 11.1 g, < 36.9 mmol, < 83 %) as a yellow powder.[3]

***R**$_f$* 0.35 (cyclohexane/acetone: 9/1); **^{1}H NMR** ($CDCl_3$, 300 MHz, 300 K): δ 8.35–8.30 (m, 4H), 7.85–7.79 (m, 3H), 4.60 (s, 2H); **^{13}C NMR** ($CDCl_3$, 126 MHz, 300 K): δ 182.8, 182.7, 144.3, 134.7, 134.4, 134.4, 134.0, 133.6,[4] 133.3, 128.2, 127.7, 127.5,[4] 31.6; **HRMS** (pos. APCI): *m*/*z* calcd. for $C_{15}H_{10}O_2Br$ 300.9859 $[M+H]^+$, found 300.9856.

[3] The compound contained an unknown impurity. Therefore, the exact amount of substance could not be determined. However, the compound was used without further purification. NMR spectroscopy was performed on a different batch, which was purified by column chromatography and therefore the spectra in the appendix do not contain the impurity.

[4] The signals at 133.6 ppm and 127.5 ppm were interpreted as two carbon atoms each.

2-(Hydroxymethyl)anthraquinone (15)

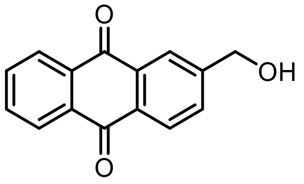

The synthesis was performed adapting a procedure by Hou *et al.* .[53] 2-(Bromomethyl)anthraquinone (**12**, 11.1 g, < 36.9 mmol) was suspended in a mixture of 1,4-dioxane (140 ml) and H_2O (140 ml). $CaCO_3$ (2.78 g, 27.5 mmol, 0.746 eq.) was added, and the mixture was stirred at reflux for 21 h. The mixture was cooled to room temperature (rt). Aq. HCl (1 M, 40 ml) was added. After CH_2Cl_2 (150 ml) was added, the phases were separated, and the aq. phase was extracted with CH_2Cl_2 (4 × 150 ml). The solvent was removed from the combined organic extracts under reduced pressure. Recrystallisation from toluene (250 ml) afforded 2-(hydroxymethyl)anthraquinone (**15**, 5.50 g, 23.1 mmol, 63 %) as orange crystals.[5]

$\boldsymbol{R_f}$ 0.35 (cyclohexane/acetone: 7/3); **^{1}H NMR** ($CDCl_3$, 400 MHz, 300 K): δ 8.31–8.26 (m, 4H), 7.82–7.78 (m, 3H), 4.89 (s, 2H); **^{13}C NMR** ($CDCl_3$, 101 MHz, 300 K): δ 183.3, 183.0, 147.8, 134.3, 134.2, 133.8, 133.7, 133.7, 132.9, 132.1, 127.8, 127.4, 127.4, 125.1, 64.6; **HRMS** (neg. APCI): *m*/*z* calcd. for $C_{15}H_{10}O_3$ 238.0635 $[M]^-$, found 238.0635.

[5] As 2-(bromomethyl)anthraquinone (**12**) was not pure, the yield cannot be calculated exactly. The yield over both reactions was 52 %.

2-(Acetylsulfanylmethyl)anthraquinone (13)

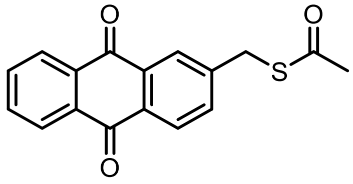

The synthesis was performed via the Mitsunobu reaction.[141] The anhydrous THF used in this reaction was purged with argon for 15 min before use. Diisopropyl azodicarboxylate (3.47 g, 16.8 mmol, 1.99 eq.) was added dropwise to a stirred solution of triphenylphosphine (4.48 g, 16.9 mmol, 2.01 eq.) in anhydrous THF (40 ml) at 0 °C. The mixture was stirred at 0 °C for 40 min, and a white precipitate formed. A suspension of 2-(hydroxymethyl)-anthraquinone (**15**, 2.01 g, 8.43 mmol) and thioacetic acid (1.35 g, 17.0 mmol, 2.02 eq.) in anhydrous THF (12.5 ml) was added dropwise over 15 min. The mixture was stirred at 0 °C for 2 h, allowed to warm up to 24 °C and stirred for 1 h. Subsequently it was poured into sat. aq. $NaHCO_3$ (200 ml), and EtOAc (100 ml) was added. The phases were separated, and the aq. phase was extracted with EtOAc (2 × 100 ml). The combined organic extracts were washed with brine (200 ml), dried over $MgSO_4$ and the solvent was removed under reduced pressure. Column chromatography (SiO_2, cyclohexane/EtOAc: 19/1 to 17/3) afforded 2-(acetylsulfanylmethyl)anthraquinone (**13**, 1.94 g, 6.54 mmol, 78 %) as a white solid.[6]

[6] The product was contaminated with diisopropyl bicarbamate. This was taken into account, and the yield was corrected according to the intergral ratio in the ^{1}H NMR-spectrum. NMR spectroscopy was performed using a pure fraction of the product of the Mitsunobu reaction, therefore the impurity is not observed in the spectra below.

An alternative synthesis was performed via nucleophilic substitution following a procedure by Brogan *et al.* .[54] KSAc (1.02 g, 8.84 mmol, 1.04 eq.) was added to a suspension of 2-(bromomethyl)anthra- quinone (**12**, 2.57 g, < 8.84 mmol) in anhydrous acetone (125 ml). The mixture was stirred at reflux for 2 h. The mixture was cooled to rt, H_2O (100 ml) was added and the mixture was extracted with EtOAc (3 × 150 ml). The combined organic extracts were washed with brine (300 ml), dried over $MgSO_4$ and the solvent was removed under reduced pressure. Column chromatography (SiO_2, d = 5 cm, h = 16.5 cm; cyclohexane/EtOAc: 19/1 to 1/1) afforded 2-(acetylsulfanylmethyl)-anthraquinone (**13**, 1.65 g, 5.57 mmol, 65 %) as a yellow solid.[7]

***R**$_f$* 0.20 (cyclohexane/acetone: 9/1); **^{1}H NMR** ($CDCl_3$, 400 MHz, 300 K): δ 8.32–8.27 (m, 2H), 8.24 (d, J = 8.0 Hz, 1H), 8.21 (d, J = 2.0 Hz, 1H), 7.81–7.77 (m, 2H), 7.73 (dd, J = 8.0, 1.9 Hz, 1H), 4.25 (s, 2H), 2.38 (s, 3H); **^{13}C NMR** ($CDCl_3$, 101 MHz, 300 K): δ 194.4, 183.0, 182.8, 145.0, 134.6, 134.3, 134.2, 133.9, 133.7, 132.6, 128.0, 127.5, 127.4, 33.3, 30.5; **HRMS** (pos. APCI): m/z calcd. for $C_{17}H_{16}O_3NS_1$ 314.0845 $[M+NH_4]^+$, found 314.0843.

[7] As 2-(bromomethyl)anthraquinone (**12**) was not pure, the yield cannot be calculated exactly. The yield over both reactions was 50 %.

2-(Acetylsulfanylmethyl)-11,11,12,12-tetracyanoanthraquinodimethane (14)

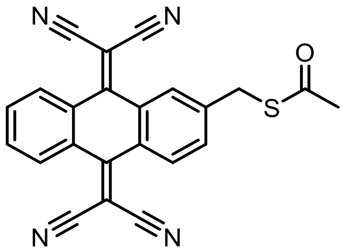

2-(Acetylsulfanylmethyl)anthraquinone (**13**, 500 mg, 1.67 mmol) and malononitrile (1.07 g, 16.0 mmol, 9.56 eq.) were dissolved in anhydrous $CHCl_3$ (57 ml). Anhydrous pyridine (2.7 ml, 2.6 g, 34 mmol, 20 eq.) and $AlCl_3$ (2.19 g, 16.4 mmol, 9.83 eq.) were added. The mixture was stirred at reflux for 14 h. The mixture was cooled to rt, H_2O (40 ml) was added, and the mixture was filtered through a plug of celite. The phases were separated, and the aq. phase was extracted with $CHCl_3$ (2 × 50 ml). The combined organic extracts were concentrated under reduced pressure. Column chromatography (SiO_2, *d* = 5.5 cm, *h* = 18 cm; cyclohexane/acetone: 9/1) afforded 2-(acetylsulfanylmethyl)-11,11,12,12-tetracyanoanthraquinodimethane (**14**, 330 mg, 0.841 mmol, 50 %) as a yellow solid.

$\boldsymbol{R_f}$ 0.40 (cyclohexane/acetone: 7/3); **^{1}H NMR** ($CDCl_3$, 500 MHz, 300 K): δ 8.25–8.22 (m, 2H), 8.18–8.16 (m, 2H), 7.75–7.71 (m, 2H), 7.64 (dd, *J* = 8.2, 1.7 Hz, 1H), 4.20 (br s, 2H), 2.38 (s, 3H); **^{13}C NMR** ($CDCl_3$, 126 MHz, 300 K): δ 194.6, 160.1, 159.9, 144.2, 132.7, 132.6, 132.6, 130.8, 130.4, 130.3, 129.0, 128.1, 128.0, 127.8, 127.7, 113.2, 113.2, 113.1, 113.1, 83.6, 83.1, 32.9, 30.6; **HRMS** (neg. ESI): *m*/*z* calcd. for $C_{23}H_{11}ON_4S$ 391.0659 $[M-H]^-$, found 391.0660.

2-(Mercaptomethyl)-11,11,12,12-tetracyanoanthraquinodimethane (3)

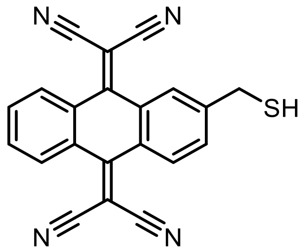

Aq. NaOH (1 M, 2.5 ml, 2.5 mmol, 5.2 eq.) was added to a stirred dispersion of 2-(acetylsulfanylmethyl)-11,11,12,12-tetracyanoanthraquinodimethane (**14**, 200 mg, 0.483 mmol) in MeOH (50 ml) at 23 °C. The mixture turned red and cleared up immediately. It was stirred for 45 s and subsequently aq. HCl (1 M, 3.75 ml, 3.75 mmol, 7.77 eq.) was added until the red color vanished and a yellow solution was obtained.[8] CH_2Cl_2 (50 ml) and H_2O (50 ml) were added. The phases were separated, and the aq. phase was extracted with CH_2Cl_2 (2 × 20 ml). The combined organic extracts were dried over $MgSO_4$, and the solvent was removed under reduced pressure. 2-(Mercaptomethyl)-11,11,12,12-tetracyanoanthraquinodimethane (**3**, 68 mg, 0.195 mmol, 40 %) was obtained after column chromatography (SiO_2, d = 2 cm, h = 42 cm; cyclohexane/CH_2Cl_2: 1/3 to CH_2Cl_2) and drying under reduced pressure at 45 °C over night as a yellow solid.

***R**$_f$* 0.46 (cyclohexane/CH_2Cl_2: 1/3); **^{1}H NMR** ($CDCl_3$, 500 MHz, 300 K): δ 8.28–8.23 (m, 2H), 8.21 (d, J = 8.2 Hz, 1H), 7.76–7.73 (m, 2H), 7.68 (dd, J = 8.1, 1.7 Hz, 1H), 3.87 (d, J = 8.0 Hz, 2H), 1.90 (t, J = 8.1 Hz, 1H); **^{13}C NMR** ($CDCl_3$, 126 MHz, 300 K): δ 160.2, 159.9, 146.7, 132.7, 132.6, 132.1, 130.9, 130.4, 130.3, 128.9, 128.2, 127.8, 127.5, 113.2, 113.2, 113.2, 113.1, 83.5, 83.1, 28.7; **HRMS** (neg. ESI): m/z calcd. for $C_{21}H_9N_4S$ 349.0553 $[M{-}H]^-$, found 349.0553.

---

[8] The reaction has not reached full conversion at this point, however longer reaction times led to unwanted side products and degradation of the product. The remaining starting material was recovered via column chromatography.

NMR Spectra

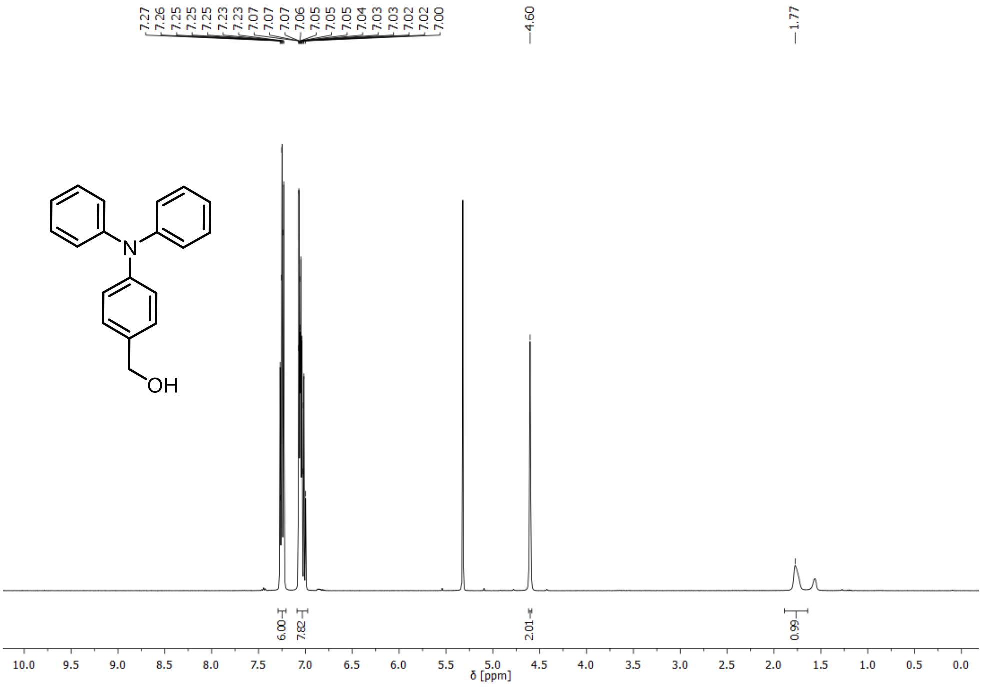

Figure S2. ^{1}H NMR spectrum of 4-hydroxymethyltriphenylamine (**5**) (CD_2Cl_2, 400 MHz, 300 K).

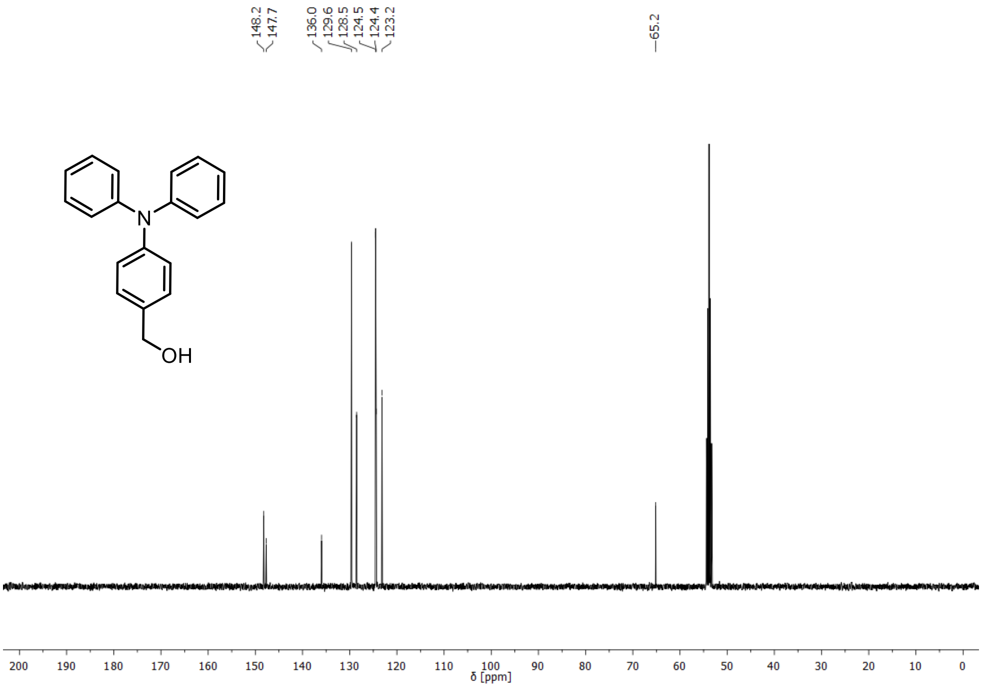

Figure S3. ^{13}C NMR spectrum of 4-hydroxymethyltriphenylamine (**5**) (CD_2Cl_2, 100 MHz, 300 K).

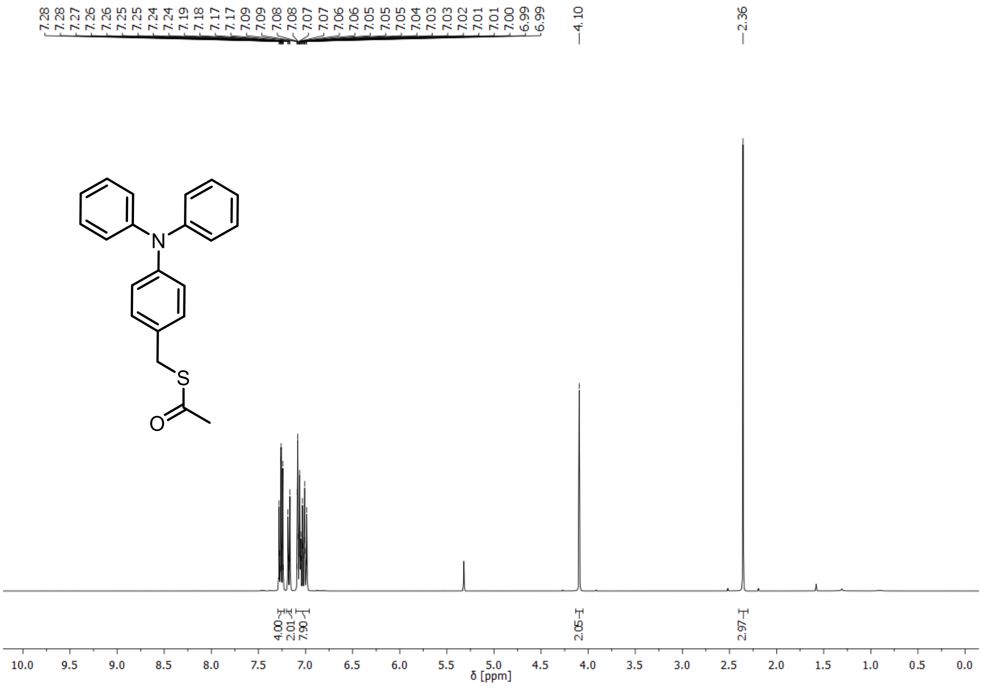

Figure S4. ^{1}H NMR spectrum of 4-(acetylsulfanylmethyl)triphenylamine (**6**) (CD_2Cl_2 400 MHz, 300 K).

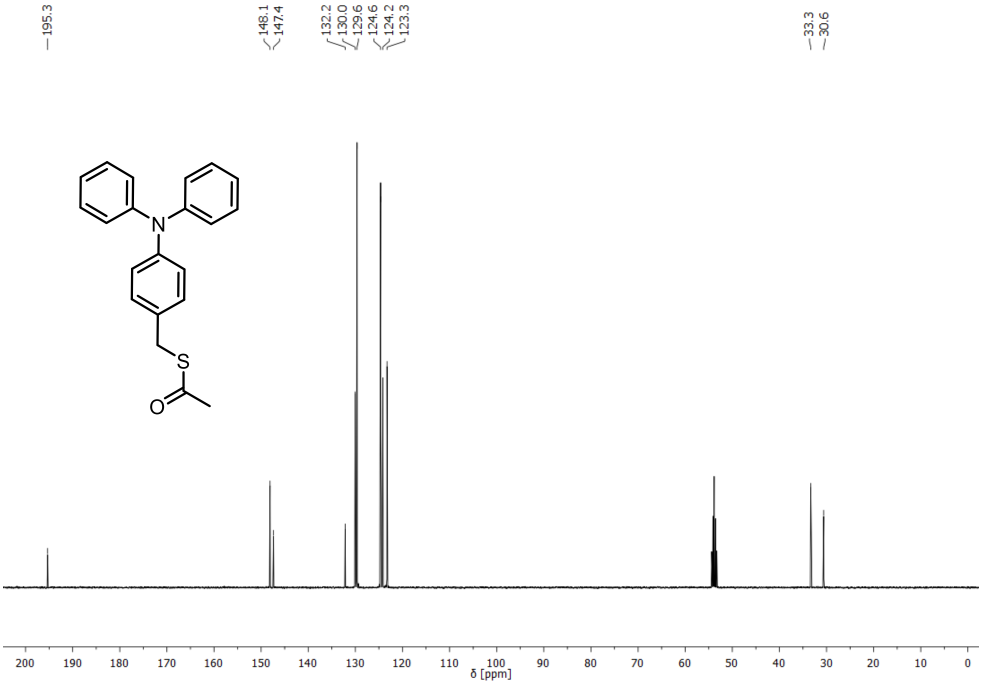

Figure S5. ^{13}C NMR spectrum of 4-(acetylsulfanylmethyl)triphenylamine (**6**) (CD_2Cl_2, 100 MHz, 300 K).

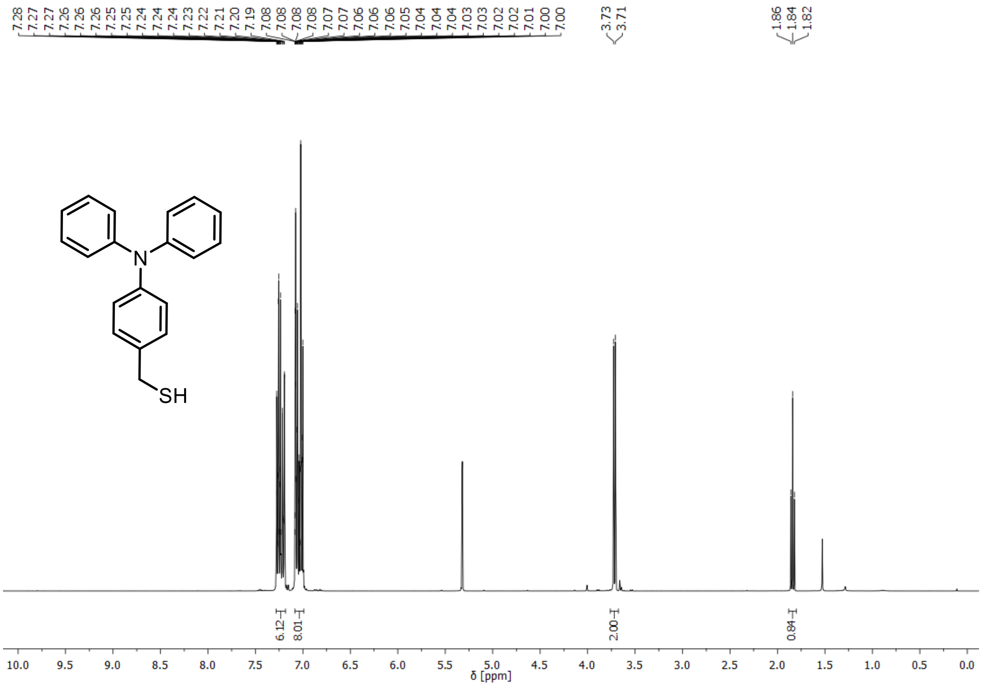

Figure S6. ^{1}H NMR spectrum of 4-(mercaptomethyl)triphenylamine (**1**) (CD_2Cl_2, 400 MHz, 300 K).

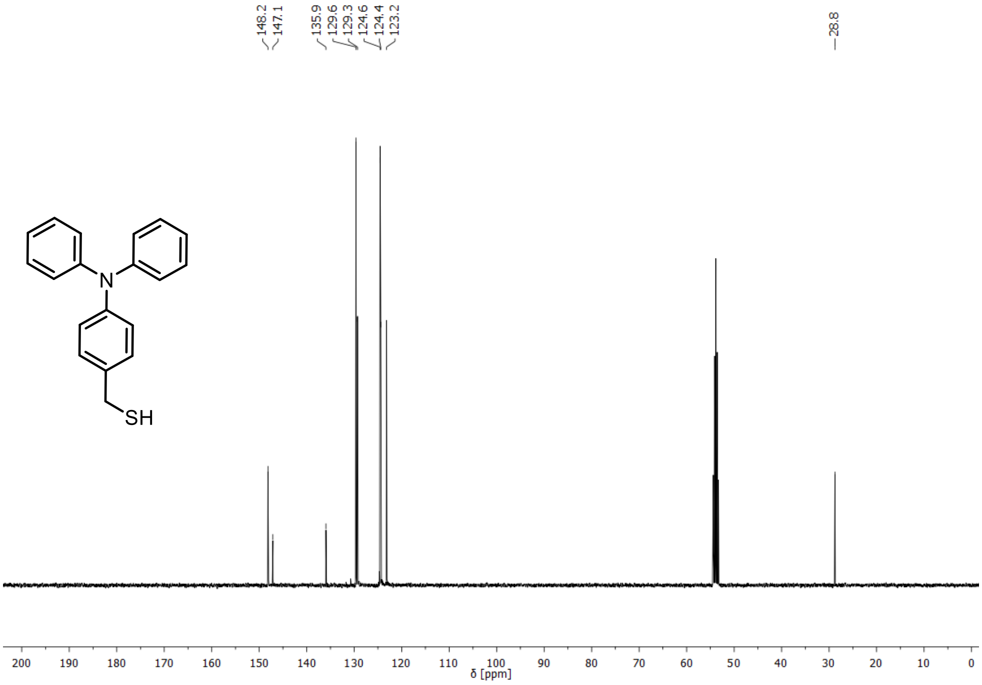

Figure S7. ^{13}C NMR spectrum of 4-(mercaptomethyl)triphenylamine (**1**) (CD_2Cl_2, 100 MHz, 300 K).

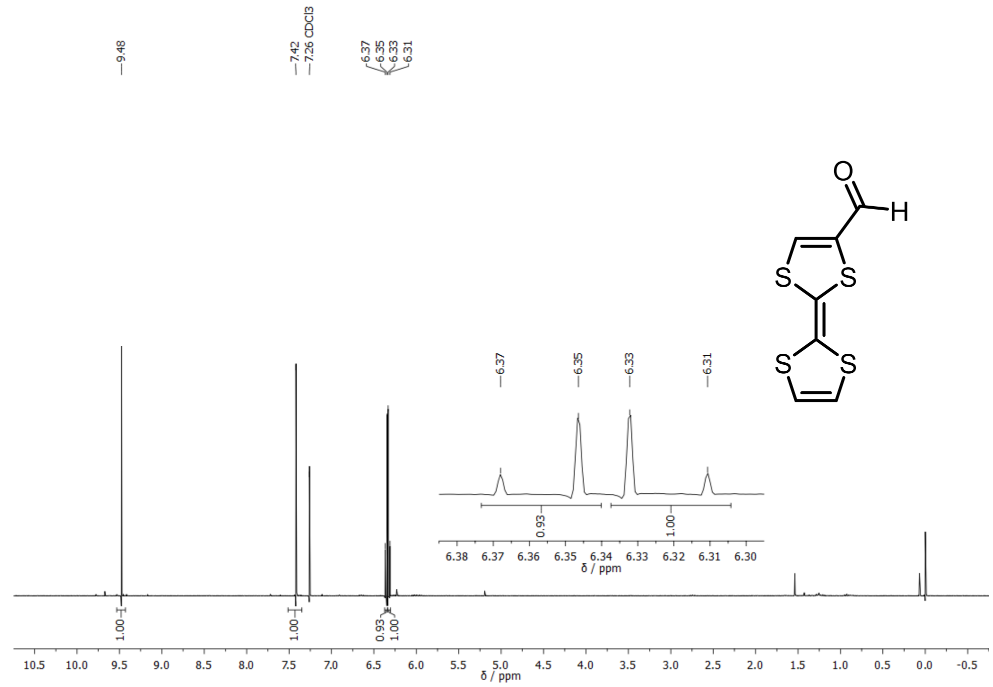

Figure S8. ^{1}H NMR spectrum of formyltetrathiafulvalene (**8**) ($CDCl_3$, 300 MHz, 300 K, contains H_2O (1.54 ppm), cyclohexane (1.43 ppm), H grease (1.25 ppm), silicone grease (0.07 ppm)).

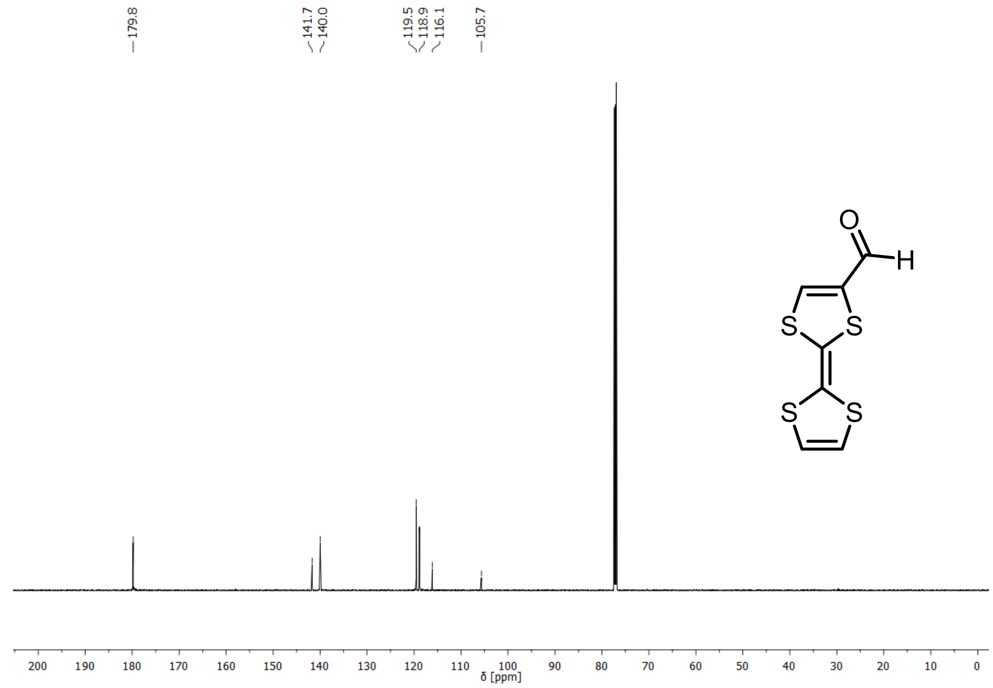

Figure S9. ^{13}C NMR spectrum of formyltetrathiafulvalene (**8**) ($CDCl_3$, 100 MHz, 300 K).

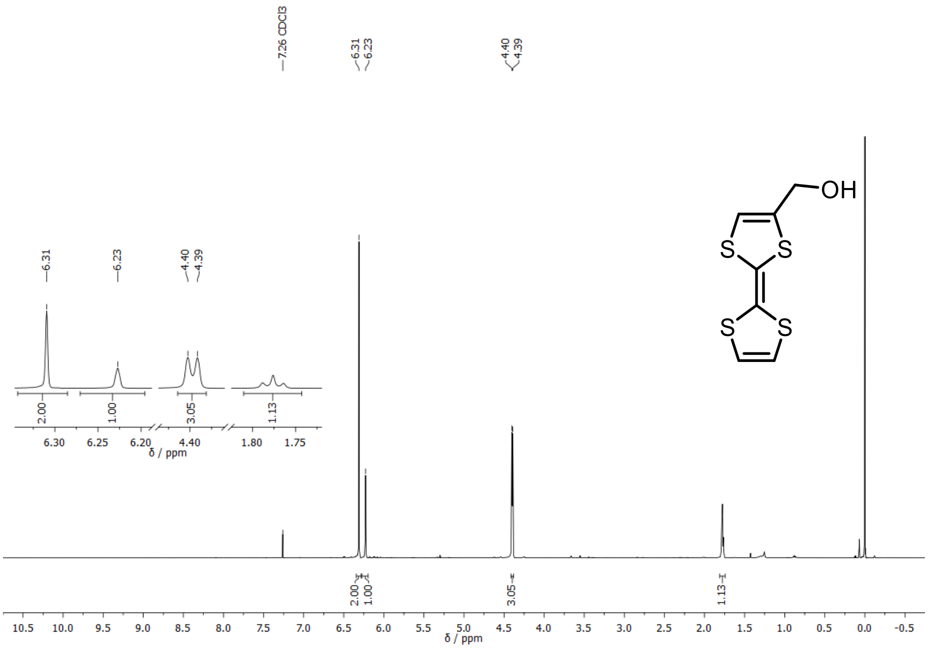

Figure S10. ^{1}H NMR spectrum of hydroxymethyltetrathiafulvalene (**9**) ($CDCl_3$, 500 MHz, 300 K, contains CH_2Cl_2 (5.30 ppm), cyclohexane (1.43 ppm), H grease (1.25 ppm), silicone grease (0.07 ppm)).

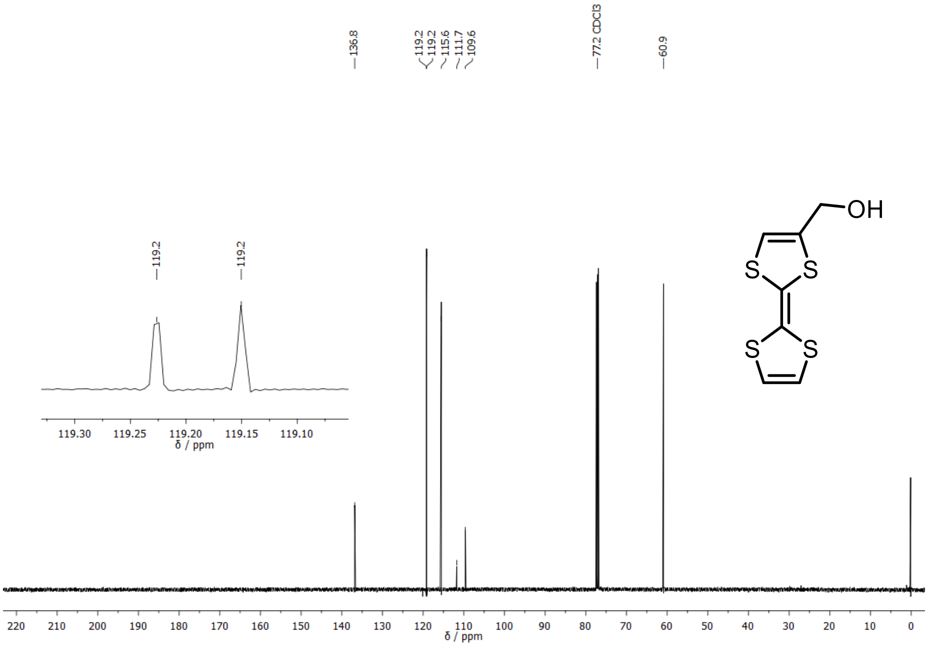

Figure S11. ^{13}C NMR spectrum of hydroxymethyltetrathiafulvalene (**9**) ($CDCl_3$, 126 MHz, 300 K).

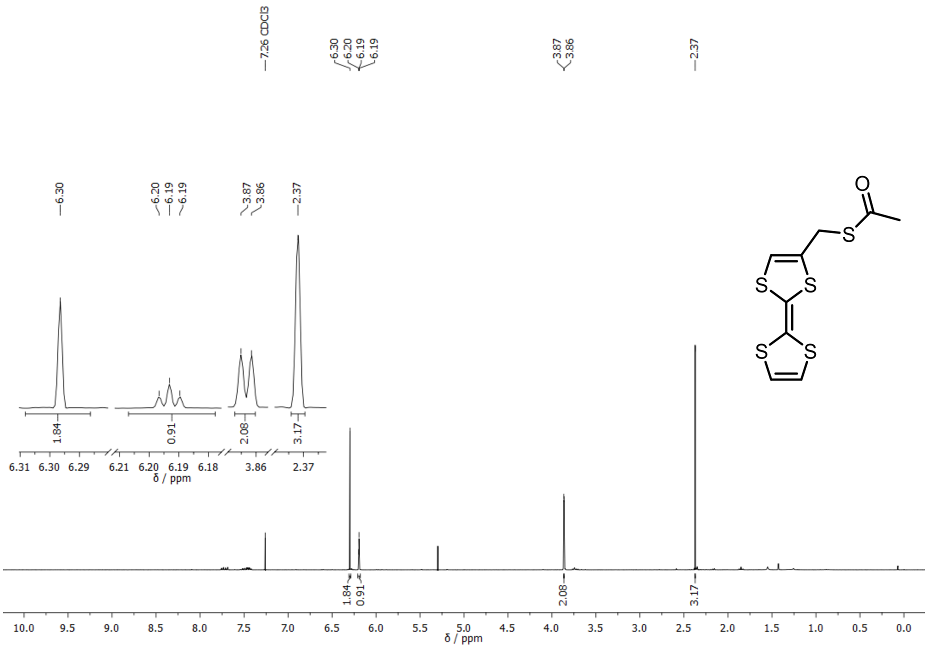

Figure S12. ^{1}H NMR spectrum of acetylsulfanylmethyltetrathiafulvalene (**10**) ($CDCl_3$, 300 MHz, 300 K, contains SP(Ph)$_3$ (7.77-7.68 and 7.54-7.41 ppm), CH_2Cl_2 (5.30 ppm), THF (3.74 and 1.85 ppm) cyclohexane (1.43 ppm), H grease (1.25 ppm) and silicon grease (0.07 ppm)).

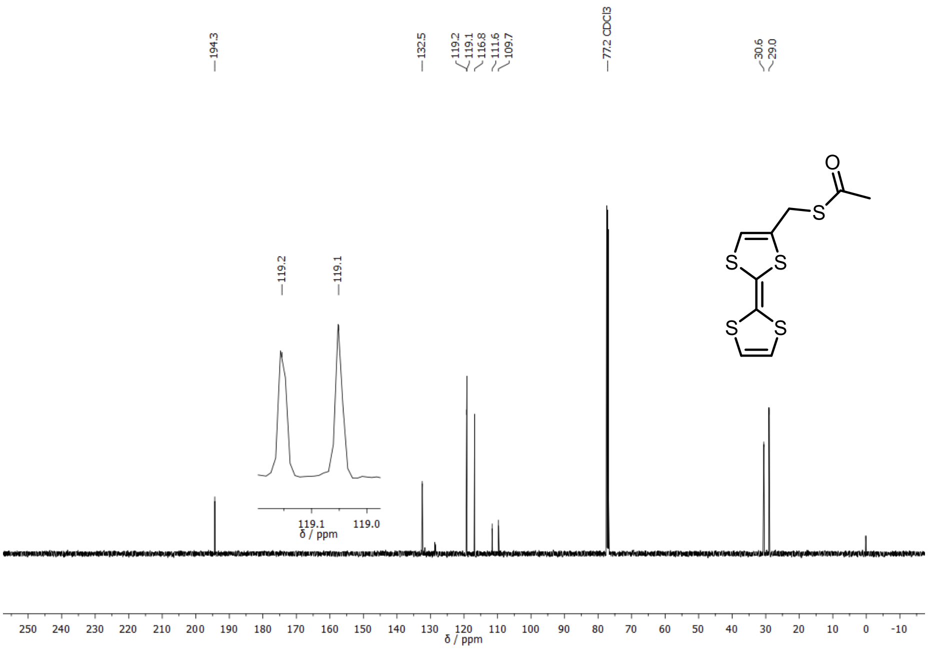

Figure S13. ^{13}C NMR spectrum of acetylsulfanylmethyltetrathiafulvalene (**10**) ($CDCl_3$, 126 MHz, 300 K, contains SP(Ph)$_3$ (132.4, 131.7, 128.7 and 128.6 ppm)).

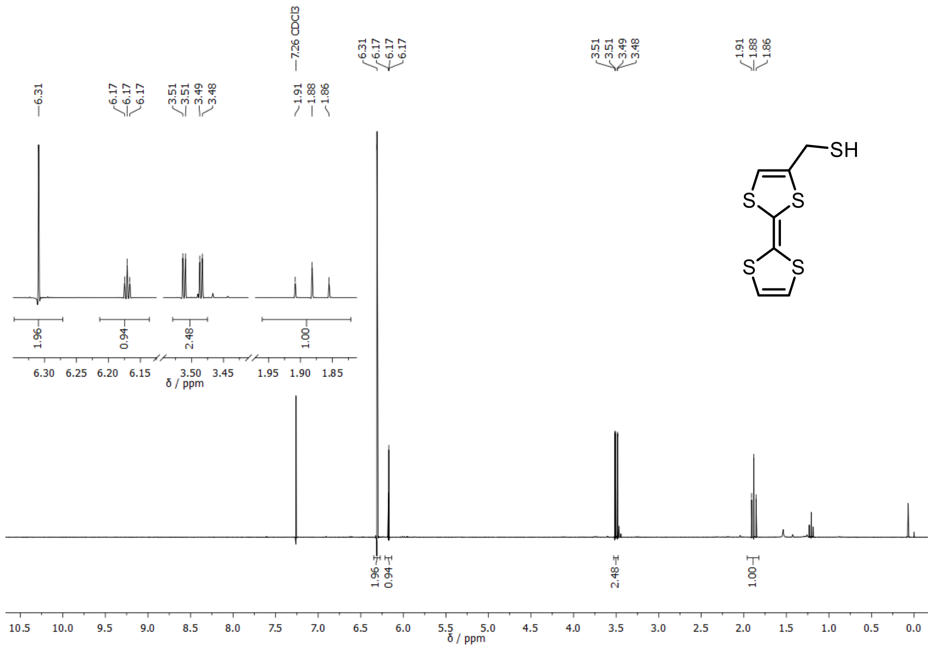

Figure S14. ^{1}H NMR spectrum of mercaptomethyl tetrathiafulvalene (**2**) ($CDCl_3$, 300 MHz, 300 K, contains Et_2O (3.48 and 1.21 ppm) cyclohexane (1.43 ppm), H grease (1.26 ppm) and silicon grease (0.07 ppm)).

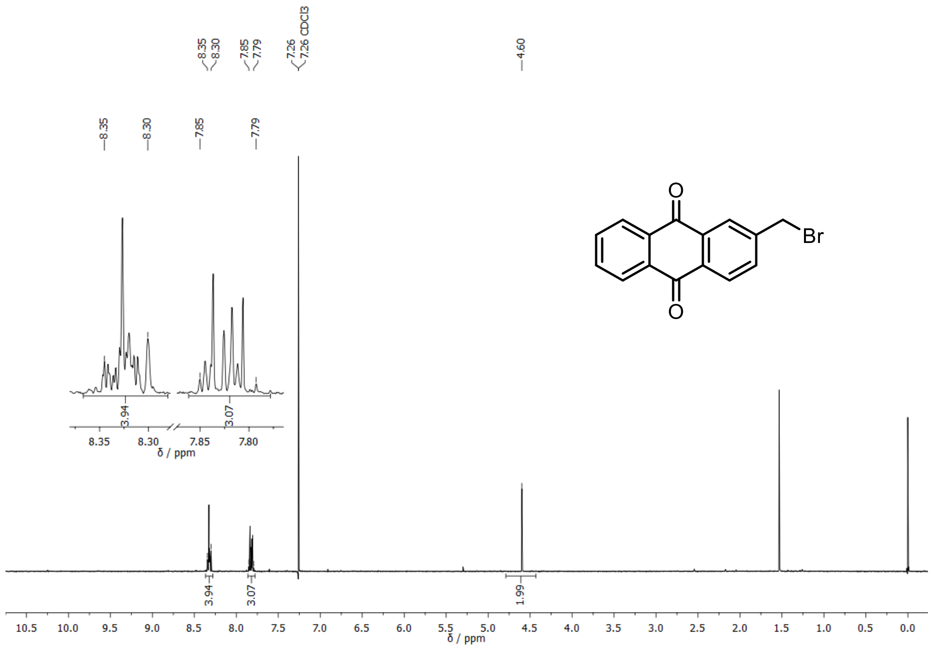

Figure S15. ^{1}H NMR spectrum of 2-(bromomethyl)anthraquinone (**12**) ($CDCl_3$, 300 MHz, 300 K, contains CH_2Cl_2 (5.30 ppm) and H_2O (1.56 ppm)).

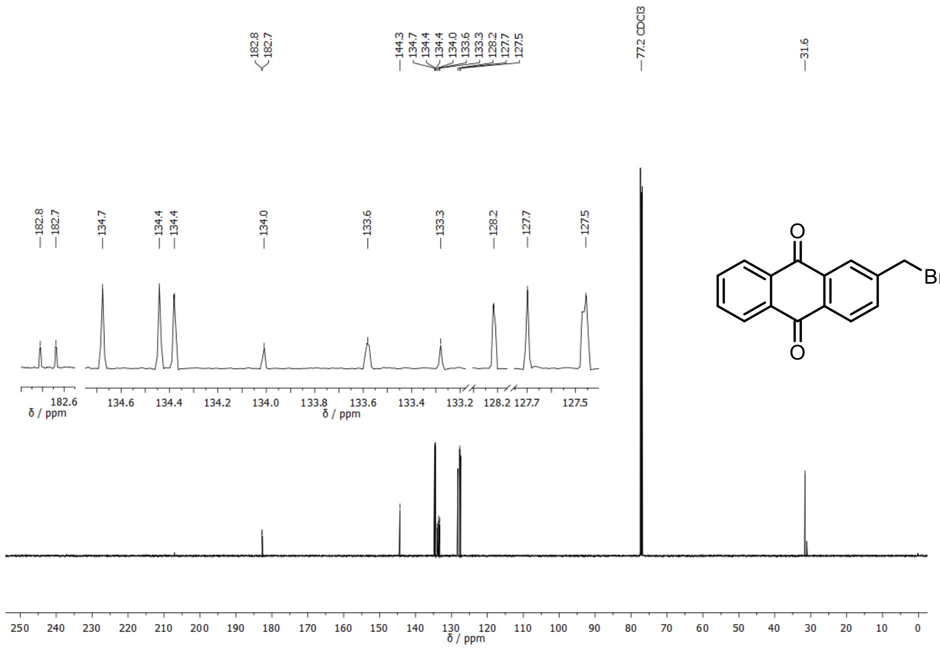

Figure S16. ^{13}C NMR spectrum of 2-(bromomethyl)anthraquinone (**12**) ($CDCl_3$, 126 MHz, 300 K, contains acetone (207.1 and 31.1 ppm)).

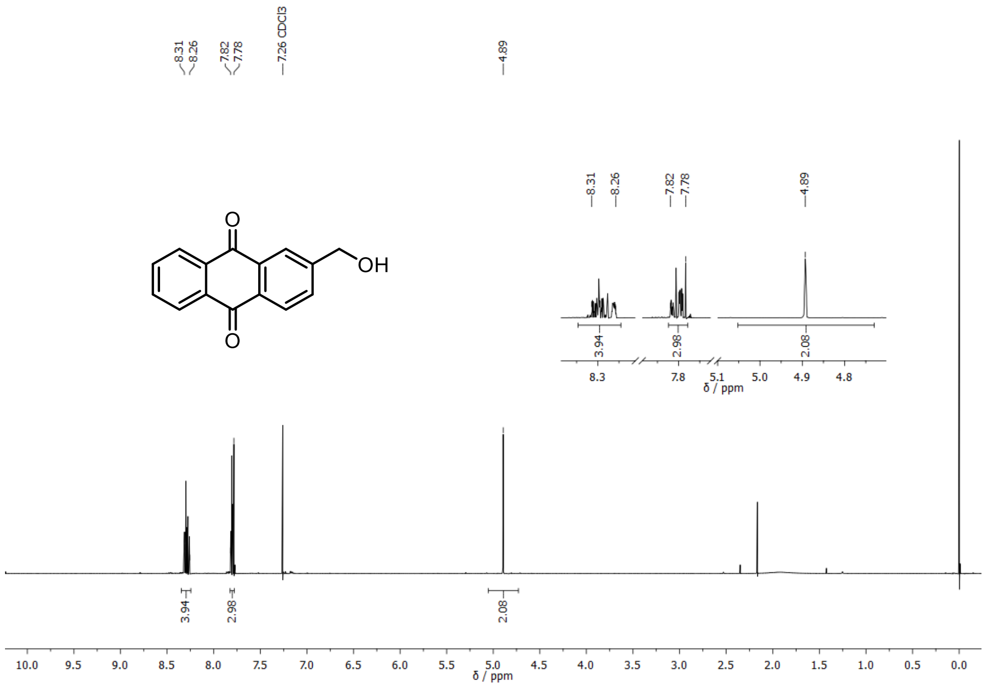

Figure S17. ^{1}H NMR spectrum of 2-(hydroxymethyl)anthraquinone (**15**) ($CDCl_3$, 400 MHz, 300 K, contains acetone (2.17 ppm), cyclohexane (1.43 ppm) and H grease (1.25 ppm)).

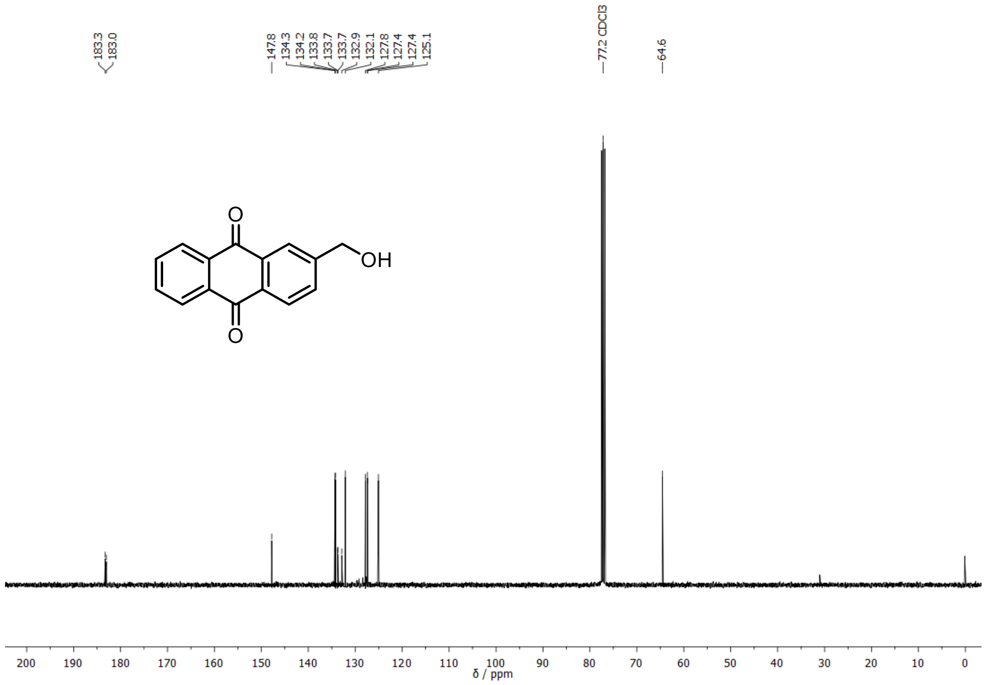

Figure S18. ^{13}C NMR spectrum of 2-(hydroxymethyl)anthraquinone (**15**) ($CDCl_3$, 101 MHz, 300 K, contains acetone (31.0 ppm)).

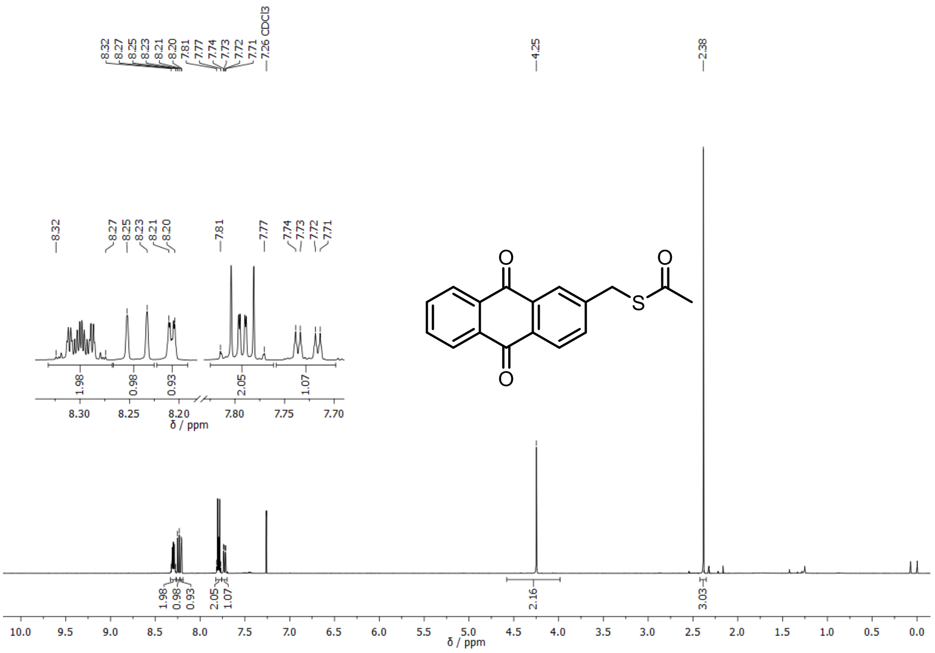

Figure S19. ^{1}H NMR spectrum of 2-acetylsulfanylmethylanthraquinone (**13**) ($CDCl_3$, 400 MHz, 300 K, contains acetone (2.17 ppm), cyclohexane (1.43 ppm), H grease (1.25 ppm) and silicone grease (0.07 ppm)).

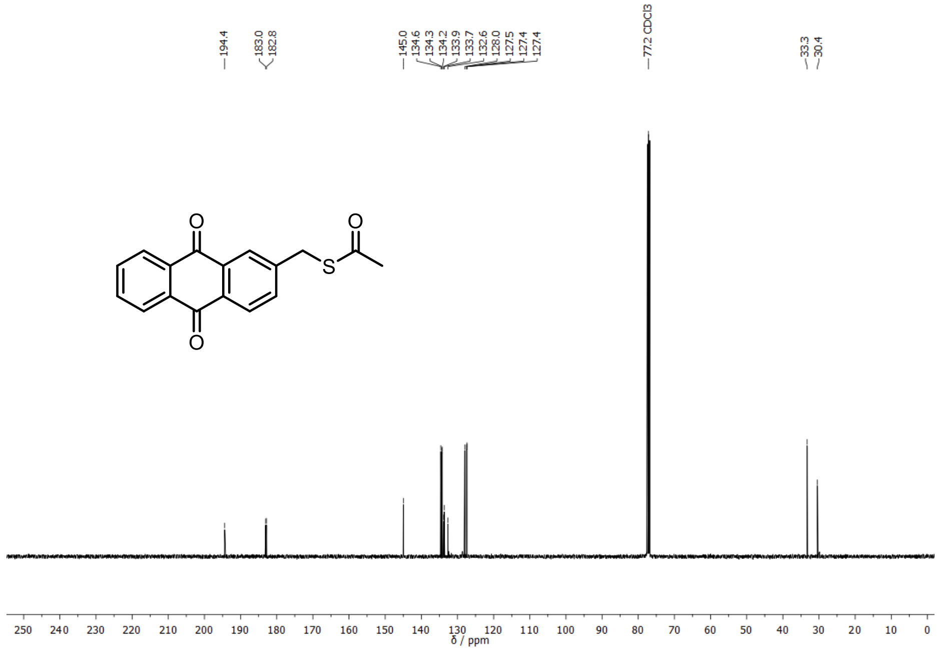

Figure S20. ^{13}C NMR spectrum of 2-acetylsulfanylmethylanthraquinone (**13**) ($CDCl_3$, 101 MHz, 300 K).

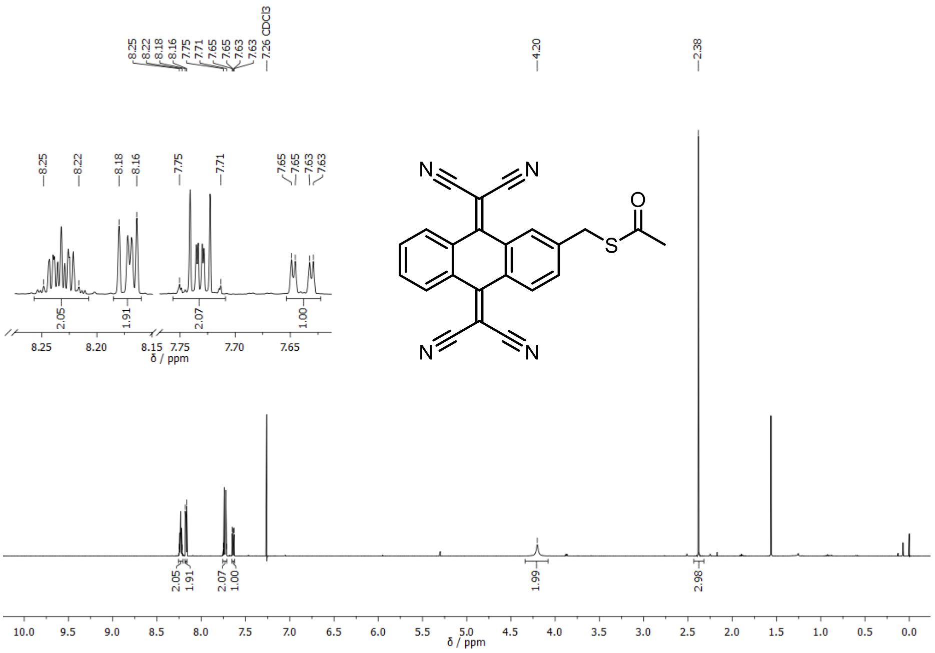

Figure S21. ^{1}H NMR spectrum of 2-(acetylsulfanylmethyl)-11,11,12,12-tetracyanoanthraquinodimethane (**14**) ($CDCl_3$, 500 MHz, 300 K, contains CH_2Cl_2 (5.30 ppm), H_2O (1.56 ppm), H grease (1.25 ppm) and silicone grease (0.07 ppm)).

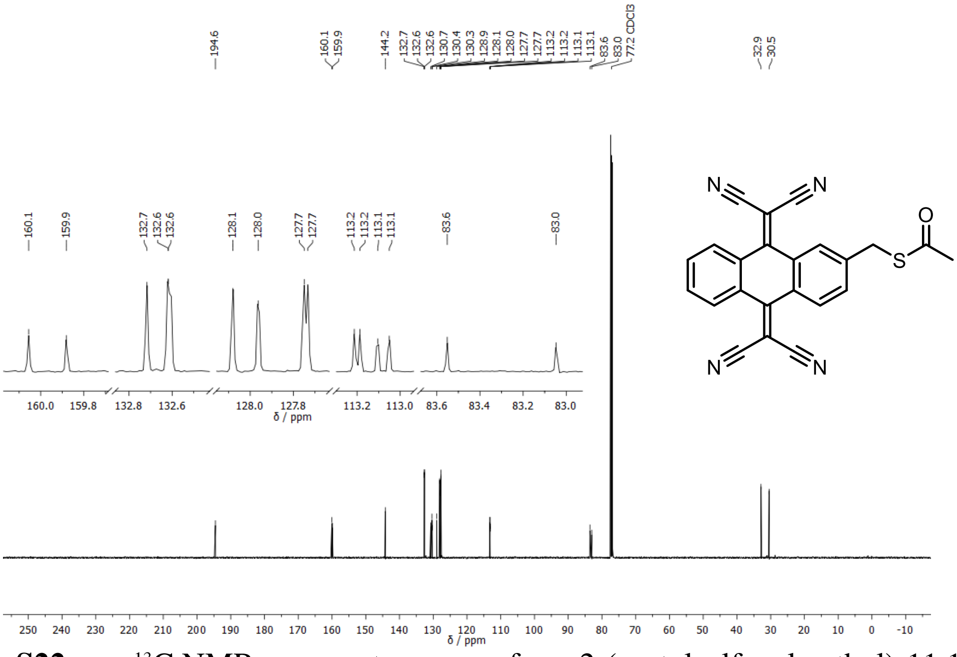

Figure S22. ^{13}C NMR spectrum of 2-(acetylsulfanylmethyl)-11,11,12,12-tetracyanoanthraquinodimethane (**14**) ($CDCl_3$, 126 MHz, 300 K).

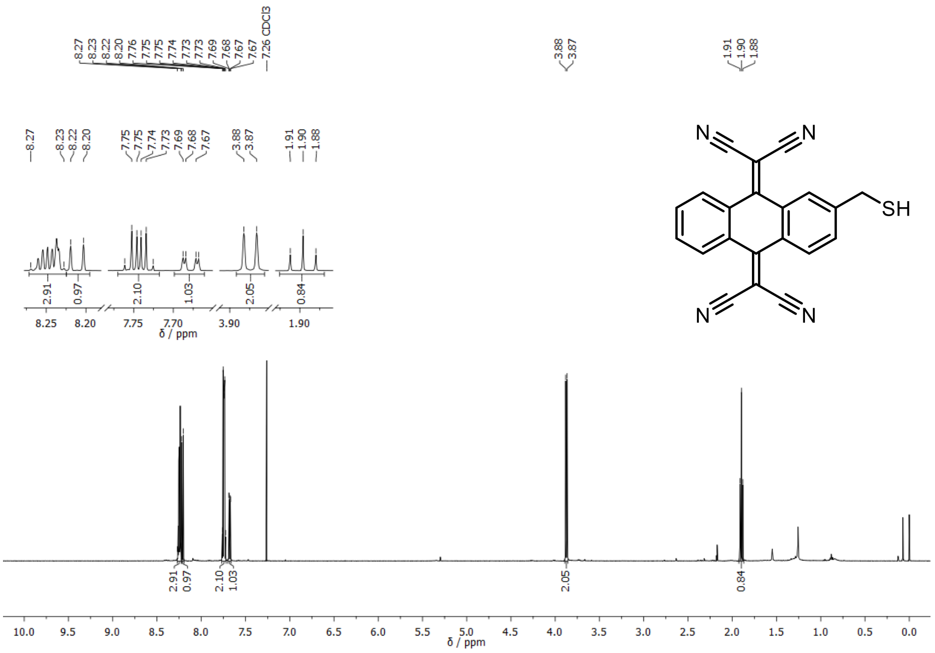

Figure S23. ^{1}H NMR spectrum of 2-(mercaptomethyl)-11,11,12,12-tetracyanoanthraquinodimethane (**3**) ($CDCl_3$, 500 MHz, 300 K, contains CH_2Cl_2 (5.30 ppm), acetone (2.17 ppm), H_2O (1.56 ppm), H grease (1.25 ppm) and silicone grease (0.07 ppm)).

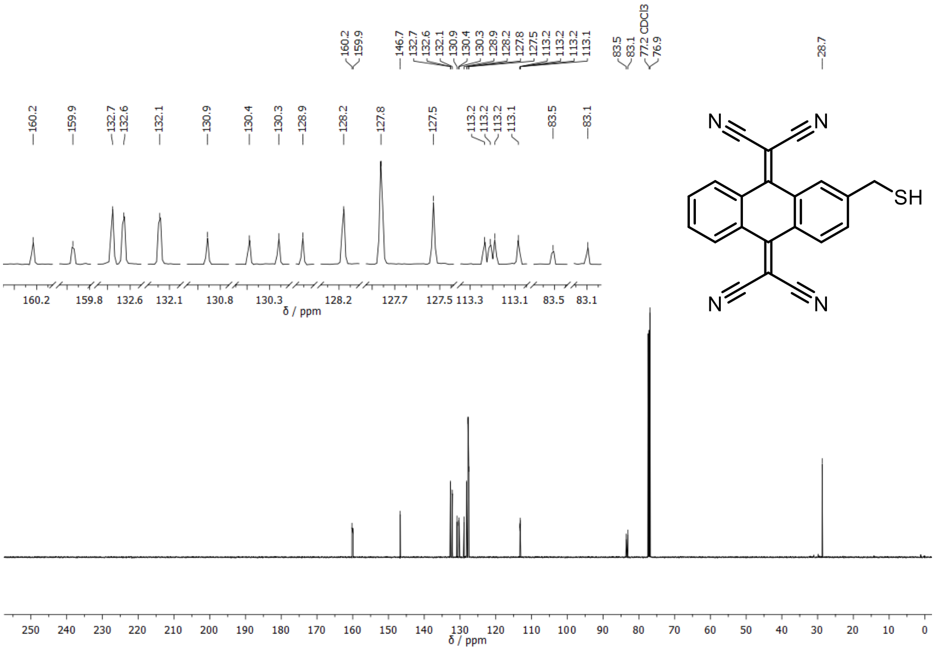

Figure S24. ^{13}C NMR spectrum of 2-(mercaptomethyl)-11,11,12,12-tetracyanoanthraquinodimethane (**3**) ($CDCl_3$, 126 MHz, 300 K).

3. Electrochemical Characterization of RAM-CH_2SH via CV

TPA-CH_2SH (**1**) and TTF-CH_2SH (**2**) are p-type molecules with chemically reversible oxidations. TPA-CH_2SH (**1**) can be reversibly oxidized at a redox potential of 0.51 V vs. Fc/Fc$^+$. The redox potential was estimated as the average between the cathodic and anodic peak potential ($E^0 \approx E_{1/2} = (E_{p,c} + E_{p,a})/2$) (Table S1-S3). TTF-$CH_2SH$ (**2**) can be chemically reversibly oxidized in two consecutive one-electron processes. The first one forms a radical cation and the second a dication at redox potentials of –0.08 V and 0.35 V vs. Fc/Fc$^+$ in CH_2Cl_2. TCAQ-CH_2SH (**3**) was chemically reversibly reduced in a two-electron process at a potential of –0.84 V vs. Fc/Fc$^+$. Based on the data from the CV (Fig. S25), the HOMO energies for TPA-CH_2SH (**1**) and TTF-CH_2SH (**2**) were estimated to be –5.27 eV and –4.68 eV, respectively. For TCAQ-CH_2SH (**3**) the LUMO energy is –3.92 eV. These values were estimated from the redox potentials, assuming an ionization energy of 4.76 eV for ferrocene.[60]

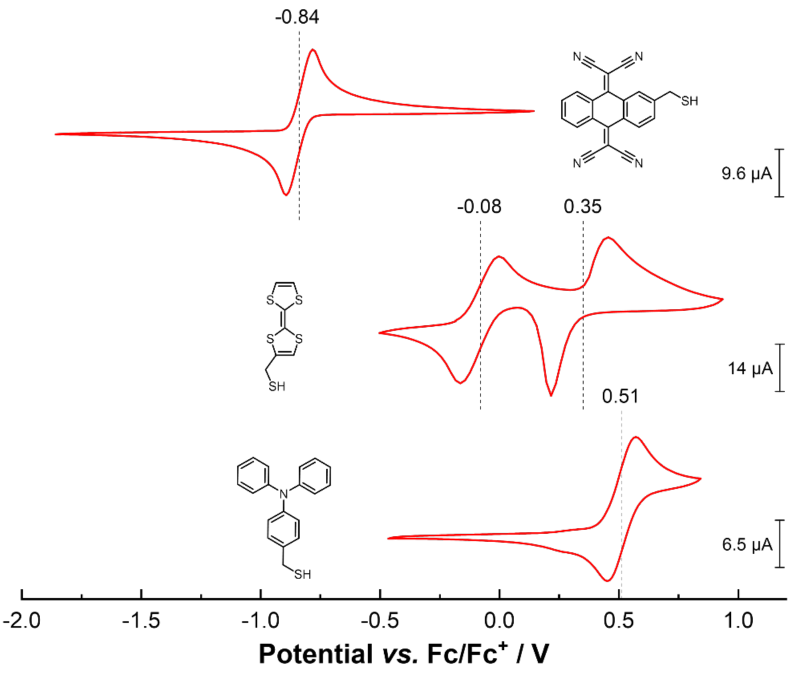

Figure S25. Electrochemical characterization of RAM-CH_2SH. CV of TPA-CH_2SH (**1**, 1.00 mM in CH_2Cl_2), TTF-CH_2SH (**2**, 1.17 mM in CH_2Cl_2), TCAQ-CH_2SH (**3**, 0.74 mM in CH_2Cl_2) using 0.1 M n-Bu_4NPF_6, scan rate 0.1 V s^{-1}, glassy carbon electrode, referenced to Fc/Fc$^+$ as internal standard.

Table S1. Electrochemical data for TPA-CH_2SH (**1**).

E_{pa} [V][a]	E_{pc} [V][b]	$E_{1/2}$ [V]	E_{HOMO} [eV][c]
0.57	0.45	0.51	–5.27

[a]Anodic peak potential. [b]Cathodic peak potential. [c]Calculated from the redox potential of the oxidation, assuming an ionization energy of 4.76 eV for ferrocene.

Table S2. Electrochemical data for TTF-CH_2SH (**2**).

$E_{pa,1}$ [V][a]	$E_{pa,2}$ [V]	$E_{pc,1}$ [V][b]	$E_{pc,2}$ [V]	$E_{1/2,1}$ [V]	$E_{1/2,2}$ [V]	E_{HOMO} [eV][c]
–0.01	0.44	0.25	–0.14	–0.08	0.35	–4.68

[a]Anodic peak potential. [b]Cathodic peak potential. [c]Calculated from the redox potential of first oxidation process, assuming an ionization energy of 4.76 eV for ferrocene.

Table S3. Electrochemical data for TCAQ-CH_2SH (**3**).

E_{pc} [V][a]	E_{pa} [V][b]	$E_{1/2}$ [V]	E_{LUMO} [eV][c]
–0.89	–0.78	–0.84	–3.92

[a]Cathodic peak potential. [b]Anodic peak potential. [c]Calculated from the redox potential of the reduction, assuming an ionization energy of 4.76 eV for ferrocene.

4. DFT Calculations of Molecules

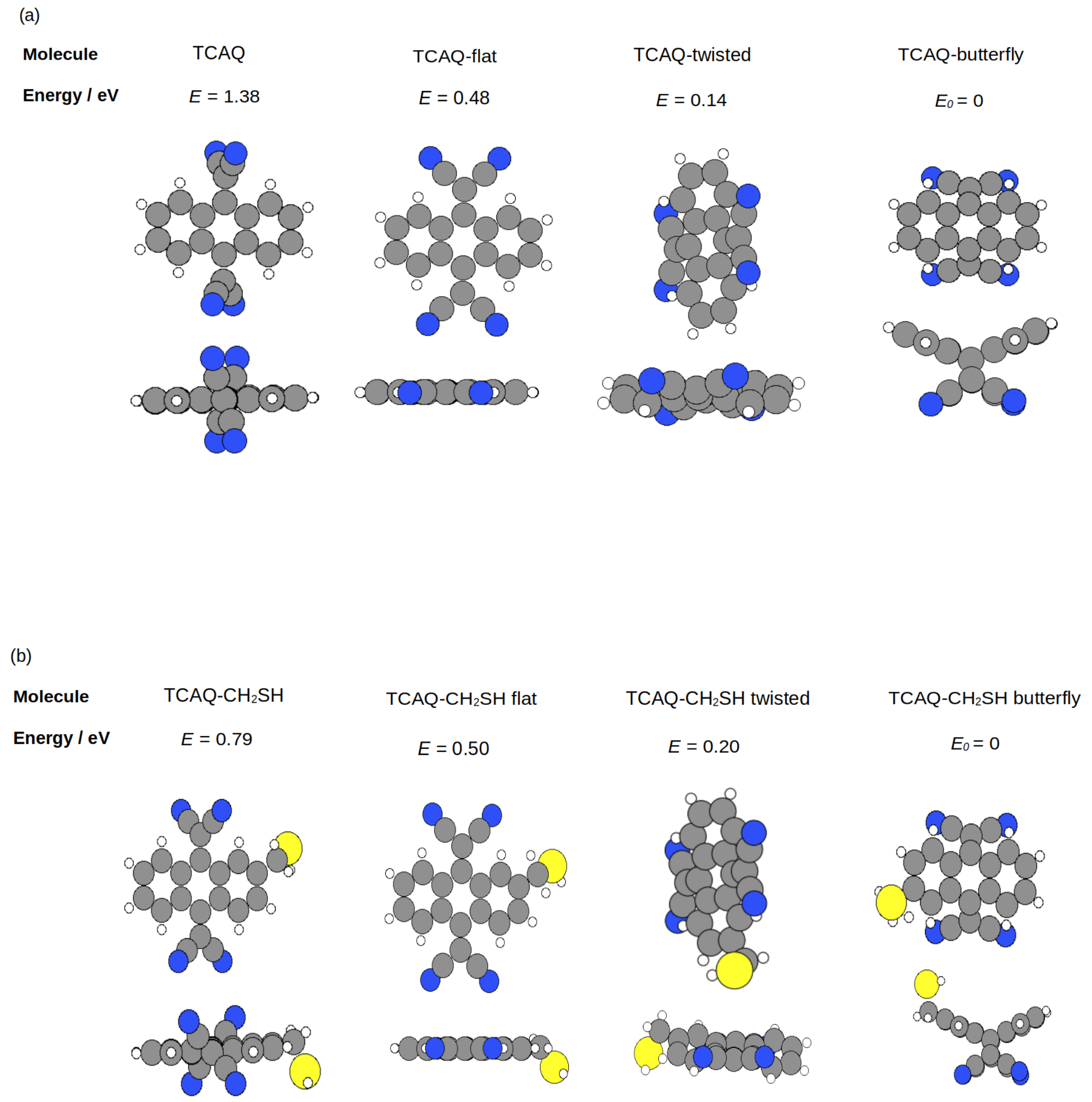

Figure S26. Conformers of TCAQ (a) and TCAQ-CH_2SH (**3**) (b) with their respective relative energies (in eV) specified on top of each structure. The potential energy of the most stable structure is denoted as E_0, with the potential energies of other conformers represented as E.

To determine the minimum energy conformation for TCAQ, we analyzed various conformations, as illustrated in Fig. S26 a. After thorough calculations, we identified that the TCAQ-butterfly conformation has the lowest potential energy. TCAQ-butterfly and TCAQ-

flat correspond to the C_{2V} and D_{2h} structures, respectively, as described by Ortí *et al.* . [142] TCAQ-twisted is similar to the D_2 structure, which is considered a transition state in the paper, derived from a planar form but with the rings twisted so that the arms are not in the same plane. TCAQ has higher potential energy than TCAQ-flat (D_{2h}) and, although not fully planar, retains significant symmetry.

We extended this analysis to examine different conformations of TCAQ with an attached CH_2SH group, as depicted in Fig. S26 b. While the structure of TPA-CH_2SH (**1**) and TTF-CH_2SH (**2**) do not have much freedom, TCAQ-CH_2SH (**3**) may adopt different conformers, as depicted in Fig. S26 b. Similar to the non-substituted TCAQ (Fig. S26 a), the neutral form of TCAQ-CH_2SH (**3**) has its lowest potential energy for the butterfly conformation. The twisted structure closely follows with a potential energy of only 0.2 eV higher than the butterfly structure.

5. DFT Calculations of Functionalized Au(111)

K points convergence test

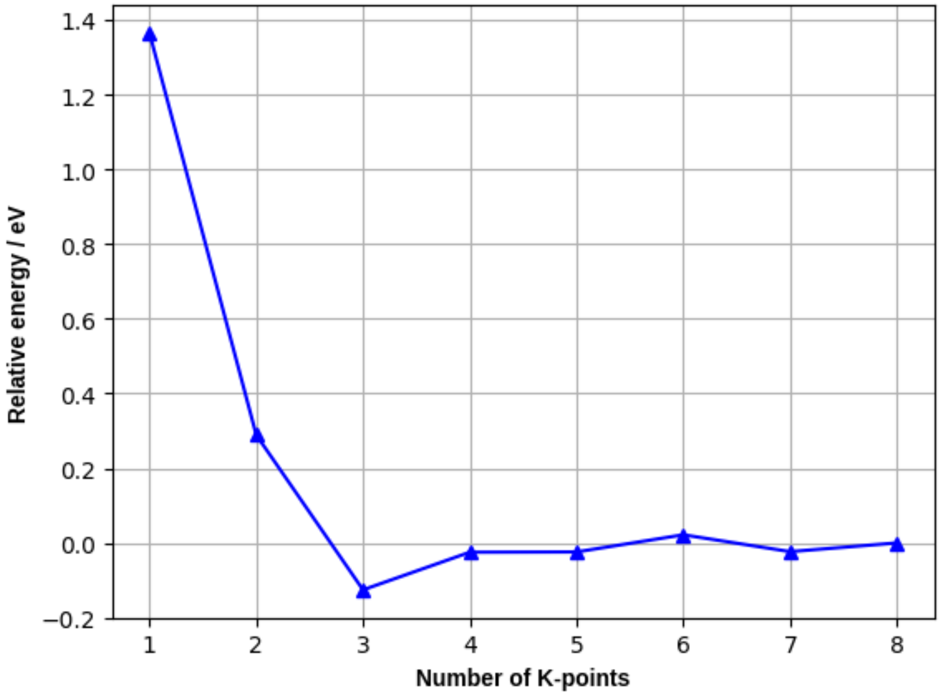

Figure S27. Relative energy convergence of SCH_3 on an Au(111) surface with varying k-points in *x* and *y* directions.

To ensure the accuracy of our DFT calculations, we performed a convergence test for the k-point sampling of the Brillouin zone for the SCH_3 molecule on the Au(111) surface. The structure under investigation consists of a 4 × 4 Au(111) surface supercell, which includes 16 Au atoms per layer, summing up to a total of 48 Au atoms distributed across three layers. The energy values were adjusted by subtracting the energy of the structure with 8 × 8 k points to highlight the relative differences. The results, as depicted in Fig. S27, indicate that the energy fluctuates at lower k-point values (1 and 2). However, starting from a 3 × 3 k-point sampling, the relative energy values show minimal variation, suggesting that the energy is converged. This stability indicates that a k-point sampling of 3 × 3 k-points is sufficient for reliable calculations, balancing computational efficiency with accuracy.

Convergence test for the number of Au layers

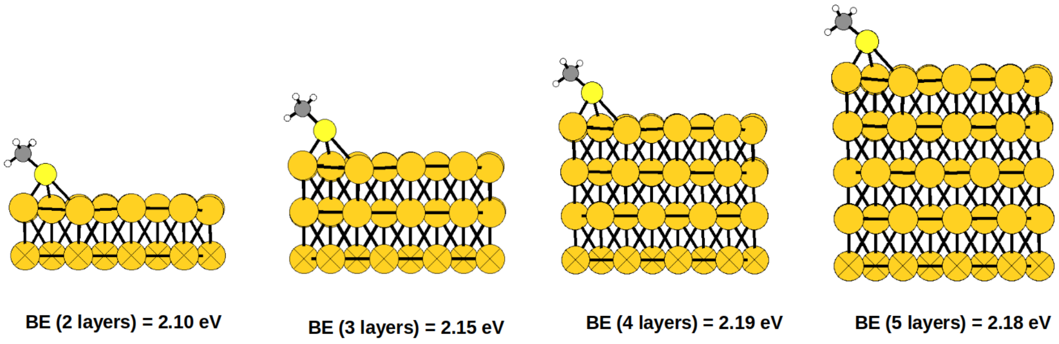

Figure S28. Schematic views of SCH_3 on Au(111) surfaces with different number of layers. Below each panel the respective binding energies (BE) are specified.

The binding energies of the SCH_3 molecule on an Au FCC(111) surface were calculated for varying numbers of Au layers to examine the convergence of the results relative to the number of layers composing the Au surface. In this simulation, we constrained the Au atoms in the bottom layer to remain fixed, ensuring that only the upper layers were allowed to relax and optimize during the calculations. This approach was chosen to simulate a more realistic surface interaction while maintaining computational efficiency. The binding energy values for each structure are specified below the respective structures in Fig. S28. As evident the binding energy of the SCH_3 molecule on the Au surface with four and five layers is nearly equal to the binding energy on three layers of Au, with a value of ca. 2.15 eV.

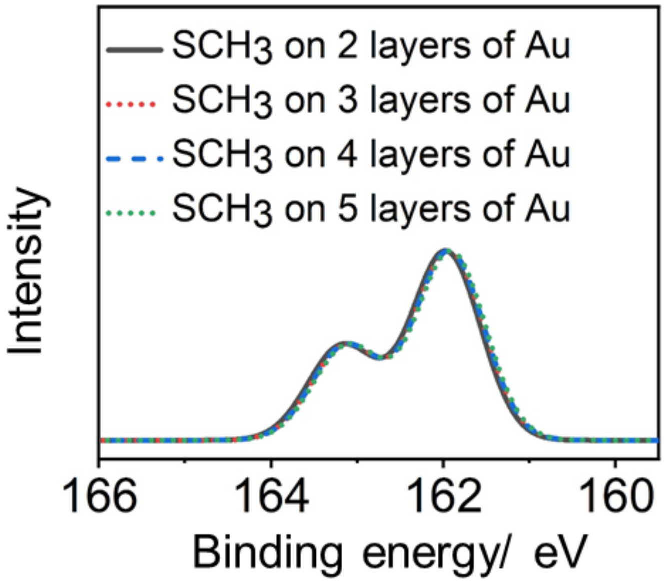

Figure S29. XPS S 2p spectra for bound SCH_3 on an Au FCC(111) surface with 2, 3, 4 and 5 layers.

In Fig. S29, the XPS S 2p spectra for the bound SCH_3 thiol on an Au FCC(111) surface depending on the number of layers is presented. The XPS spectra are indistinguishable. The binding energy of the S $2p_{3/2}$ peak for structures with three, four and five layers of Au is nearly identical: ca. 161.93 eV. The data clearly shows that the number of Au layers has a negligible effect on the binding energy. This finding is significant because it allows us to use a three-layer Au surface model for future calculations without compromising the accuracy of our results. Using fewer layers reduces computational costs and complexity, facilitating more efficient and extensive studies. Therefore, we conclude that a three-layer Au surface is sufficient to accurately model the binding energy of the SCH_3 molecule, making it a practical choice for further computational investigations.

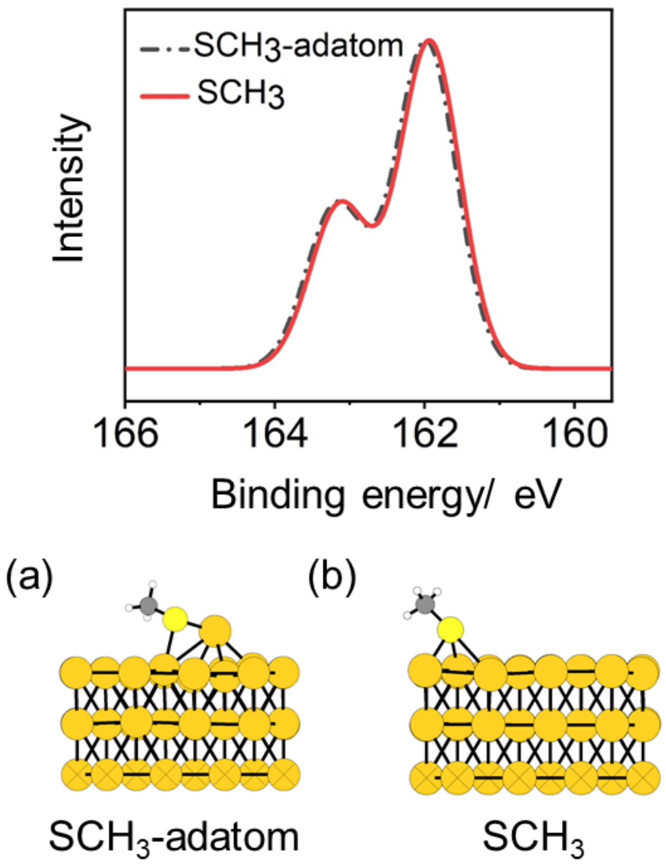

Figure S30. XPS S 2p spectra for bound SCH_3 on an Au FCC(111) surface: (a) SCH_3-adatom and (b) SCH_3.

To gain a better understanding of the effect of an adatom on an Au FCC(111) surface on the XPS spectra, we used the conformation introduced by H. Häkkinen.[143] In this conformation, one SCH_3 molecule forms bonds with both the extra Au atom and the surface. The XPS S 2p spectra of SCH_3-adatom (Fig. S30 a) and the SCH_3 (Fig. S30 b) are identical. Thus, based on the XPS S 2p results alone, we cannot distinguish the presence of an adatom in our system. This finding suggests that the XPS S 2p spectra are not sensitive to the subtle differences introduced by the adatom.

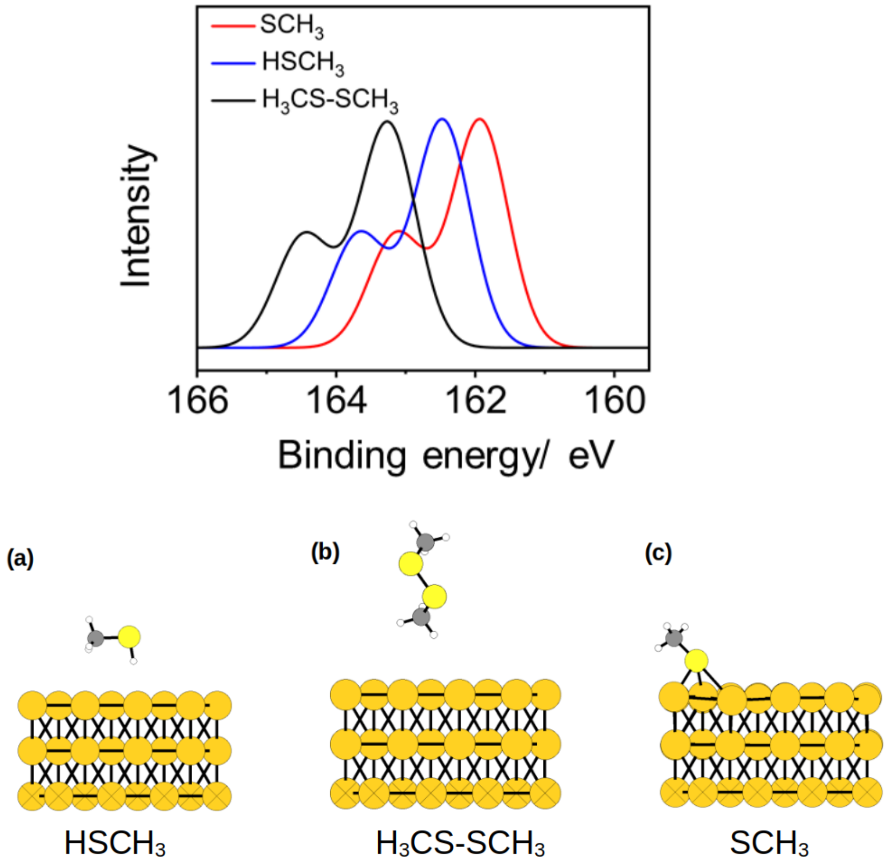

Figure S31. XPS S 2p spectra and schematics of different binding types for methanethiols on an Au FCC(111) surface: (a) $HSCH_3$ (unreacted) molecule, (b) H_3CS-SCH_3 (dimer) molecule, and (c) SCH_3 molecule bound directly to the Au surface.

We investigated the possible binding types between thiols and the Au surface, where we consider CH_3SH. There are different possibilities: the CH_3SH may remain intact, form dimers, or form a bond with the Au FCC(111) surface, as shown in Fig. S31 a to c. There, a thiol preferably binds on a hollow site on the Au surface.[144]

The simulated spectrum of unreacted methanethiol ($HSCH_3$) on the Au FCC(111) surface displays a S $2p_{3/2}$ peak at 162.47 eV, which is higher than the S $2p_{3/2}$ peak of the monomer bound to the surface, but lower than that of the dimer peak predicted in our simulations.

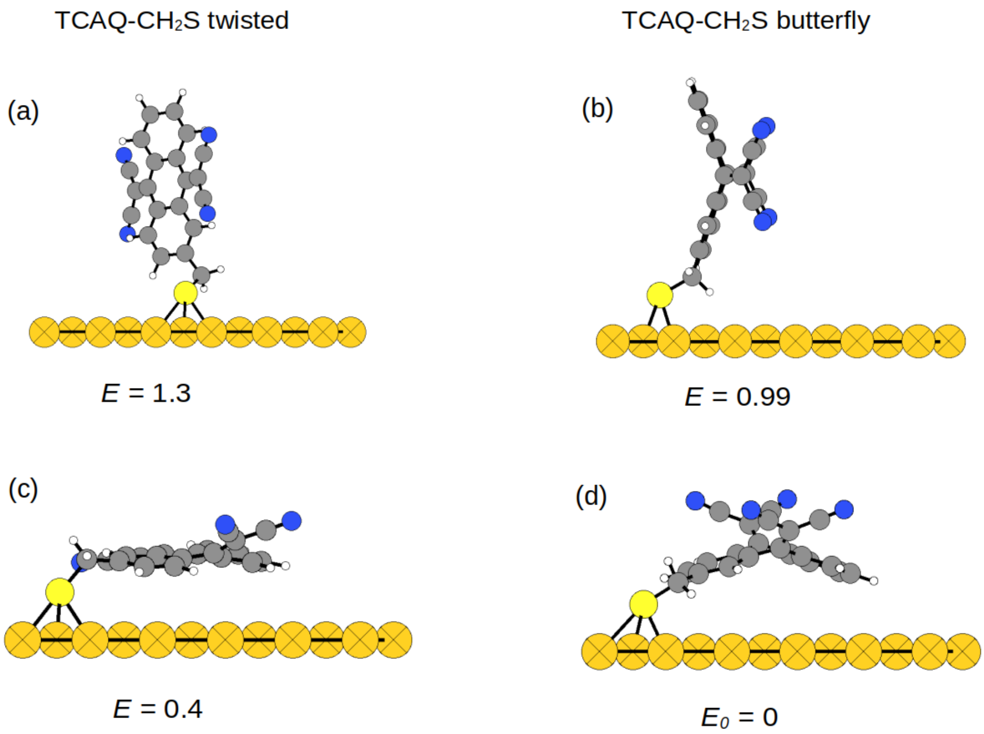

Figure S32. Different orientations of TCAQ-CH_2S twisted and TCAQ-CH_2S butterfly on one layer of Au FCC(111). Below each structure their respective relative potential energy (in eV) are specified.

For the next step of our study, we considered different orientations of TCAQ-CH_2S on the Au surface. As illustrated in Fig. S32, the two best conformations of this molecule on one layer of Au FCC(111) are twisted and butterfly. For both conformation of TCAQ-CH_2S, the potential energy of the molecule when standing on the Au surface (a and b) is approximately 1 eV higher than when the molecule lies flat on the Au surface (c and d). Notably, the TCAQ-CH_2SH (**3**) butterfly conformation, when flat on the Au surface, exhibits the minimum potential energy among all the examined conformations. To accommodate the molecule lying on the Au surface, we used the 6 × 6 × 1 Au FCC(111) surface and fixed the Au atoms to reduce computational costs.

We conducted the same calculation for TPA-CH_2SH (**1**) and TTF-CH_2SH (**2**) to determine the optimal orientation of the molecules on the Au FCC(111) surface. Our results indicate that

redox molecules lying flat on the Au surface exhibit a lower potential energy. Specifically, Fig. S33 shows that the TPA-CH_2S molecule lying flat on the Au surface has a potential energy that is 0.78 eV lower than when in an upright orientation. Similarly, Fig. S34 demonstrates that for TTF-CH_2S, the potential energy of the molecule lying flat on the Au surface is nearly 1 eV lower compared to the upright orientation.

Yuge *et al.* [52] claim that based on their results on the thickness of self-assembled monolayers (SAMs) on Au, the TTF-CH_2S molecules are standing on the Au surface, while the TCNQ molecules are lying on the Au surface. However, our calculations clearly show that redox molecules lying flat on the Au surface exhibit lower potential energies, contradicting the conclusions by Yuge *et al.* .[52]

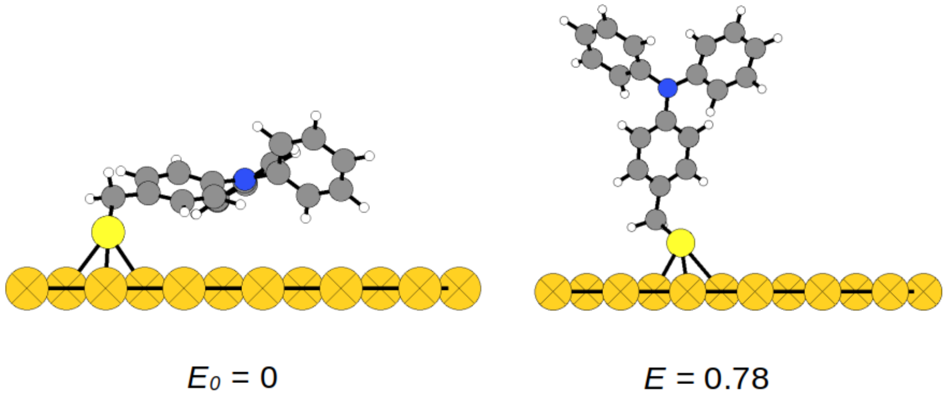

Figure S33. Different orientations of TPA-CH_2S on one layer of Au FCC(111). Below each structure their respective relative potential energy (in eV) are specified.

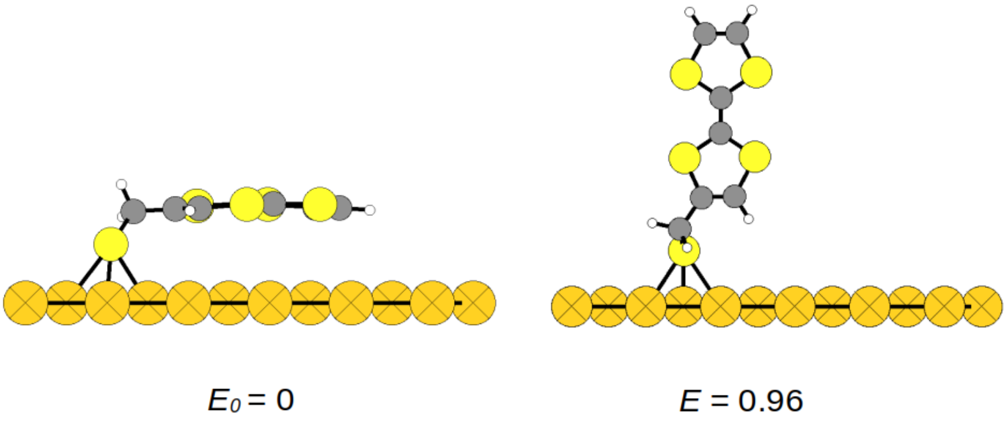

Figure S34. Different orientations of TTF-CH_2S on one layer of Au FCC(111). Below each structure their respective relative potential energy (in eV) are specified.

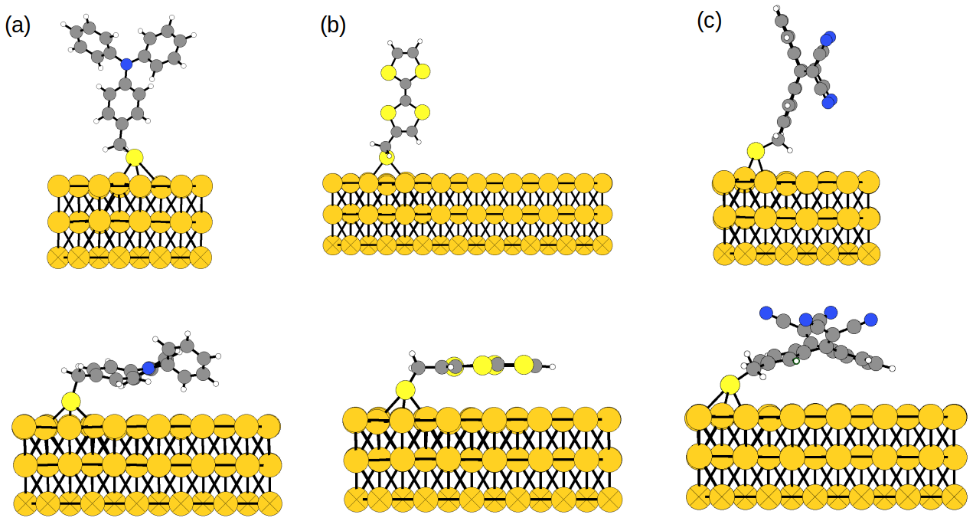

Figure S35. Different orientations of RAM-CH_2S on three layers of Au FCC(111): (a) TPA-CH_2S, (b) TTF-CH_2S, (c) TCAQ-CH_2S.

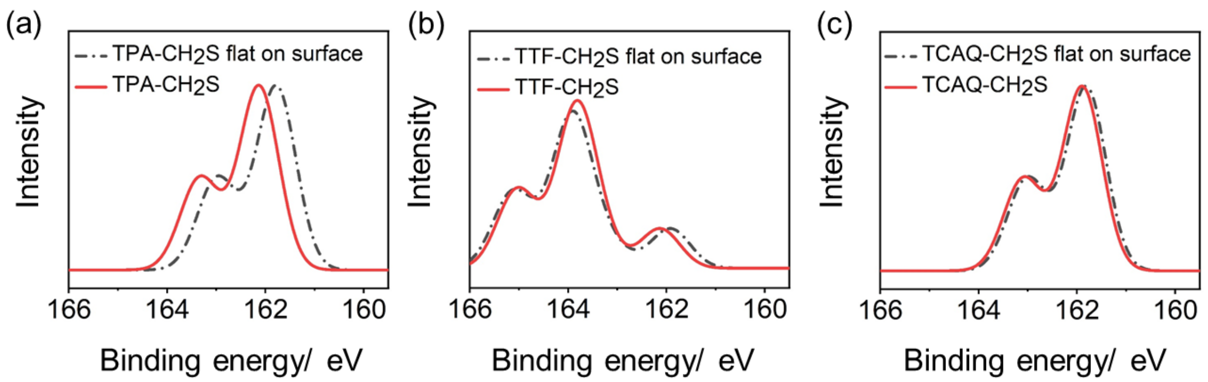

Figure S36. XPS S 2p spectra of different orientations of RAM-CH_2S on three layers of Au FCC(111): (a) TPA-CH_2S, (b) TTF-CH_2S, (c) TCAQ-CH_2S.

To investigate the effect of different orientations of RAM-CH_2S on the Au FCC(111) surface on the XPS S 2p spectra. We calculated the S 2p spectra for both orientations of the RAM-CH_2S on three layers of Au FCC(111) (Fig. S35). Fig. S36 b and c show that the S 2p spectra for both orientations of TTF-CH_2S and TCAQ-CH_2S are almost identical. However, the difference between the two binding energies of TPA-CH_2S (Fig. S36 a), is slightly higher, ca. 0.35 eV. This difference might be due to changes in the conformations of the TPA-CH_2S lying flat on the Au surface.

Similar changes have been reported for F4-TCNQ on a Cu(111) surface by Ortí *et al.* .[142] According to their review, in some organic molecules, significant conformational changes occur when adsorbed on metal surfaces, due to van der Waals attraction and charge transfer from the substrate. For example, in the F4-TCNQ/ Cu(111) system, experiments (UV photoelectron spectroscopy, X-ray standing wave) and theoretical analyses (DFT) revealed that the strong molecule-metal interaction leads to notable modifications in molecular geometry.[145] In conclusion, we can say that the XPS energies are hardly affected by the orientations of the molecules relative to the surface.

6. Hirshfeld Charge Analysis

Table S4. Hirshfeld charge analysis of RAM-CH_2S on the Au FCC(111) surface in neutral and charged states.

	**TPA (q = 0)**	**TPA (q = 1)**	**TTF (q = 0)**	**TTF (q = 1)**	**TCAQ (q = 0)**	**TCAQ (q = -1)**
**RAM**	-0.22	-0.05	+0.28	+0.42	-0.38	-0.51
**Au**	+0.22	+1.05	-0.28	+0.58	+0.38	-0.49
**Δq**		+0.83		+0.86		-0.87

We conducted a Hirshfeld charge analysis for TPA-CH_2S and TTF-CH_2S on an Au FCC(111) surface in neutral (q = 0) and positively charged (q = +1) states, as well as for TCAQ-CH_2S on an Au FCC(111) surface in neutral (q = 0) and negatively charged (q = -1) states. The results given in Table S4 reveal that 80-90% of the charge is transferred to the Au FCC(111) surface, while the charge state of the RAM changes very little. Thus, there is significant charge redistribution between the RAM and the surface.

In the KPFM experiment, the $q = 0$ state represents the pre-contact condition, while $q = +1$ and $q = -1$ represent post-contact states for the TCAQ–TTF system. Calculations indicate that most of the transferred charge originates from the Au AFM cantilever tip functionalized with TPA-CH_2S or TTF-CH_2S molecules and moves to the Au surface functionalized with TCAQ-CH_2S molecules, aligning well with experimental measurements.

7. RAM Coverage on the Au surface

To investigate the possible coverage of RAMs on the Au surface, we considered different Au surface sizes (Fig. S37 a) that are each decorated by a single RAM. A comparison of the binding energy of the RAMs to these surfaces reveals the most stable coverage: larger binding energies indicate a more probable configuration.

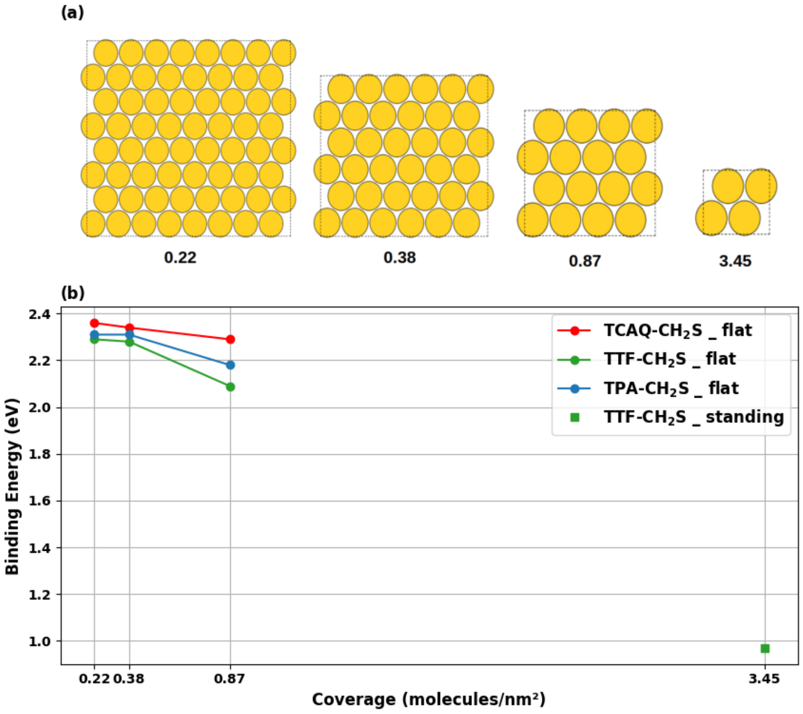

Figure S37. (a) Unit-cells of the Au surfaces considered; the numbers beneath indicate coverage (molecules/nm^2) for one molecule per cell. (b) Binding energy of RAMs on the Au surface versus coverage.

Generally, the most stable orientation for the RAMs on the Au surface is the flat one. TTF-CH_2S was the only molecule that could fit on the smallest Au surface (coverage ≈ 3.45 molecules nm^{-2}), but in a different orientation—standing on the Au surface—and its binding energy was the lowest (0.97 eV).

As can be seen in Fig. S37 b, we observe convergence of the binding energy for the RAMs in a flat orientation on the Au surface with a coverage of around 0.4 molecules nm^{-2} or lower. At higher coverages, the molecules repel each other leading to a decrease in binding energy. We therefore expect a maximal coverage of 0.4 molecules nm^{-2} from energetics.

8. Projected Density of States of RAMs on the Au Surface

To gain a better understanding of the electronic structure of our systems, we performed projected density of states (PDOS) calculations for the RAMs. In the analysis that follows, we consider only the RAMs in the flat orientation on the Au FCC(111) surface.

The most relevant states for our study are those near the Fermi level, in particular the HOMO of the donor molecules (TPA-CH_2SH (**1**) and TTF-CH_2SH (**2**)) and the LUMO of the acceptor molecule (TCAQ-CH_2SH (**3**)). To gain insight into the properties of these states when the molecules are covalently bound to the surface, it is important to identify which atoms contribute to the states near the Fermi level. Accordingly, we provide PDOS graphs showing both individual atomic contributions as well as their combined contributions to clarify the electronic structure at the RAM-Au interface. To evaluate the effect of introducing the linker to the RAMs and the Au surface, we also present PDOS graphs for the RAMs both on the Au surface and in isolation, with and without the linker. Additionally, calculations were performed in both, the gas phase as well as in implicit aqueous environment, to assess the influence of the environment on the electronic structure.

Fig. S38 compares the PDOS for TPA and TPA-CH_2SH (**1**) (Fig. S38 a and b), adding the CH_2SH linker to TPA. An extra peak from the linker can be seen in Fig. S38 b around -2.4 eV, which originates mainly from the S atom, while the HOMO of TPA-CH_2SH (**1**) is composed of C and N atoms. After adsorption on the Au surface, the S-derived state is strongly broadened, contributing mostly below but also above the Fermi level of Au. There is still a slightly broadened peak derived from N and C atoms near the Au Fermi level in Fig. S38 c, indicating minor interaction between this redox-active state of TPA with Au. The effect of H_2O is not significant.

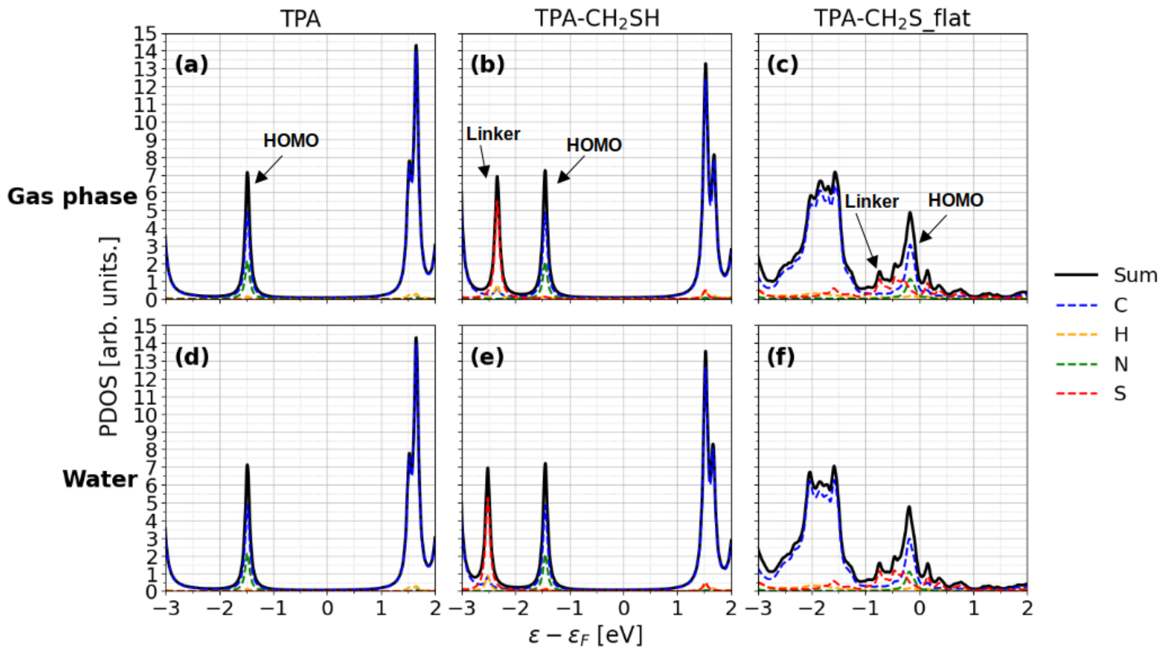

Figure S38. Projected density of states (PDOS) for TPA: (a) TPA in the gas phase; (b) TPA-CH_2SH (**1**) in the gas phase; (c) TPA-CH_2S, flat on the Au surface, in the gas phase; (d) TPA in H_2O; (e) TPA-CH_2SH (**1**) in H_2O; (f) TPA-CH_2S, flat on the Au surface, in H_2O.

Fig. S39 compares the PDOS for TTF and TTF-CH_2SH (**2**). We can see that the CH_2SH linker introduces an extra peak around -2.6 eV, while the HOMO level, important for the electron donor TTF, at -1 eV remains unchanged. The HOMO peaks are primarily contributed by the S and C atoms through the S atoms in the two upper rings of TTF. The contribution of the S atom from the linker is in the deeper states.

Broadening of the HOMO states of TTF-CH_2S and the peak related to the linker after adsorption on the Au surface is evident. Since the HOMO of TTF-CH_2S on Au lies at the Fermi level, the ionization potential of the molecule is close to the work function of the Au surface, indicating that electron loss to Au is facilitated. This is in line with TTF having a smaller ionization potential (IP) than the work function of Au, as discussed in the main text. We observe the same effects in H_2O from adding the linker to TTF and from adsorption on the Au surface (Fig. S39 d–f).

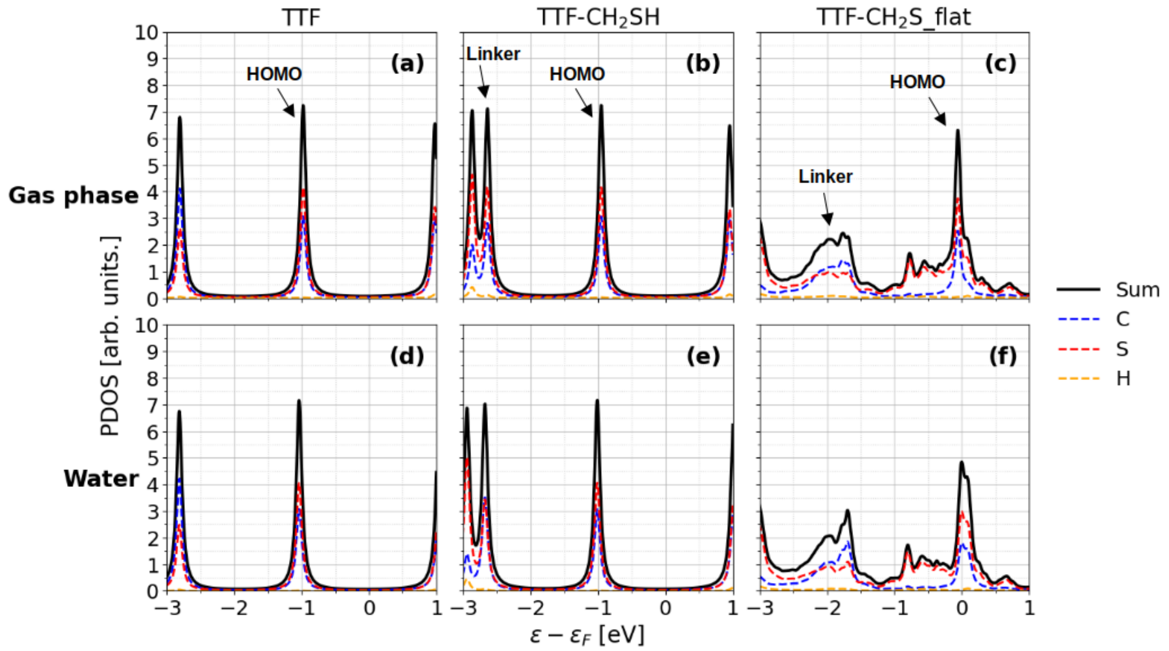

Figure S39. Projected density of states (PDOS) for TTF: (a) TTF in the gas phase; (b) TTF-CH_2SH (**2**) in the gas phase; (c) TTF-CH_2S, flat on the Au surface, in the gas phase; (d) TTF in H_2O; (e) TTF-CH_2SH (**2**) in H_2O; (f) TTF-CH_2S, flat on the Au surface, in H_2O.

As shown in Fig. S40, by comparing TCAQ and TCAQ-CH_2SH (**3**) (Fig. S40 a and b), adding the CH_2SH linker to TCAQ introduces an extra peak in the HOMO, which mainly originates from the S atom of the linker. The LUMO peak, important for the electron acceptor TCAQ, mostly composed of contributions from C and N atoms, remains intact. Fig. S40 c shows that the covalent binding of TCAQ-CH_2S to the Au surface results in a broadening of the HOMO peak due to bond formation. The LUMO of TCAQ still remains the same. Adding the linker and adsorption on the Au surface thus did not change the LUMO level of TCAQ, which is the most important peak in an acceptor (i.e., the redox activity properties of the structure did not change).

The same trend is observed in H_2O. By comparing the PDOS for each structure in the gas phase with that in H_2O, a slight shift of the LUMO peak toward the Fermi level of Au is seen in comparing Fig. S40 c and f, suggesting that the presence of H_2O in the system can enhance charge transfer from the Au surface.

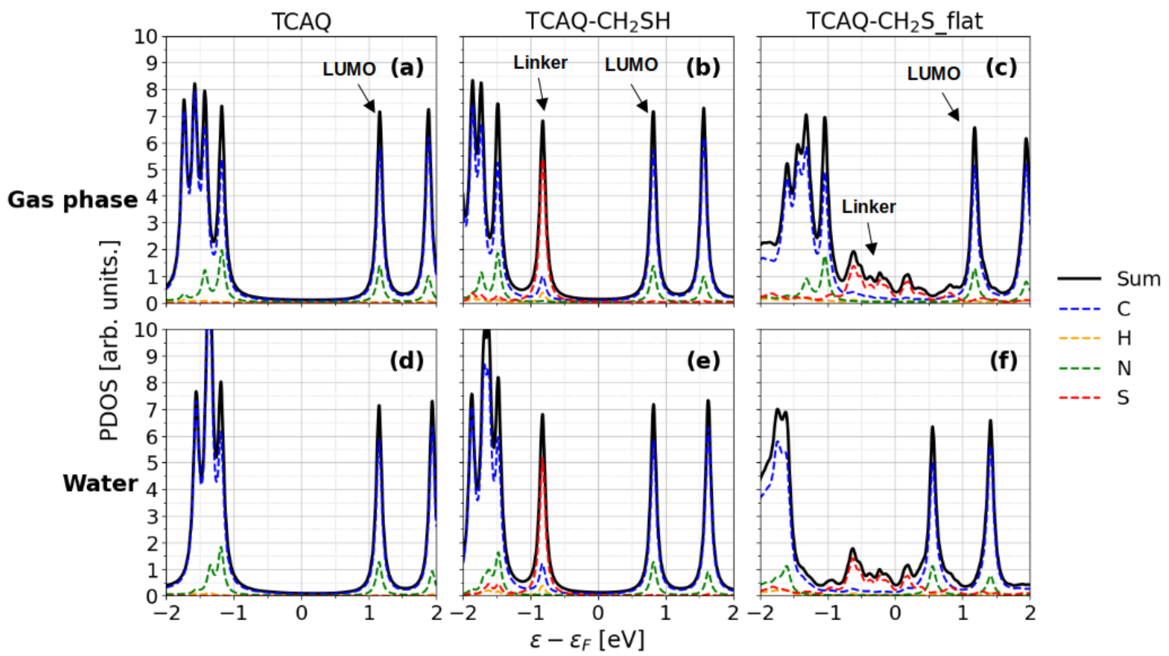

Figure S40. Projected density of states (PDOS) for TCAQ in the butterfly configuration: (a) TCAQ in the gas phase; (b) TCAQ-CH_2SH (**3**) in the gas phase; (c) TCAQ-CH_2S, flat on the Au surface, in the gas phase; (d) TCAQ in H_2O; (e) TCAQ-CH_2SH (**3**) in H_2O; (f) TCAQ-CH_2S, flat on the Au surface, in H_2O.

Overall, the linker introduces an additional state, but does not affect the LUMO of the acceptor or the HOMO of the donors. Covalent connection to Au through the linker strongly broadens the corresponding state, but keeps the redox-active states mainly intact. Nevertheless, the electron donor HOMO-levels become located just slightly below the Fermi level of Au.

9. XRD and SEM of Au Substrate

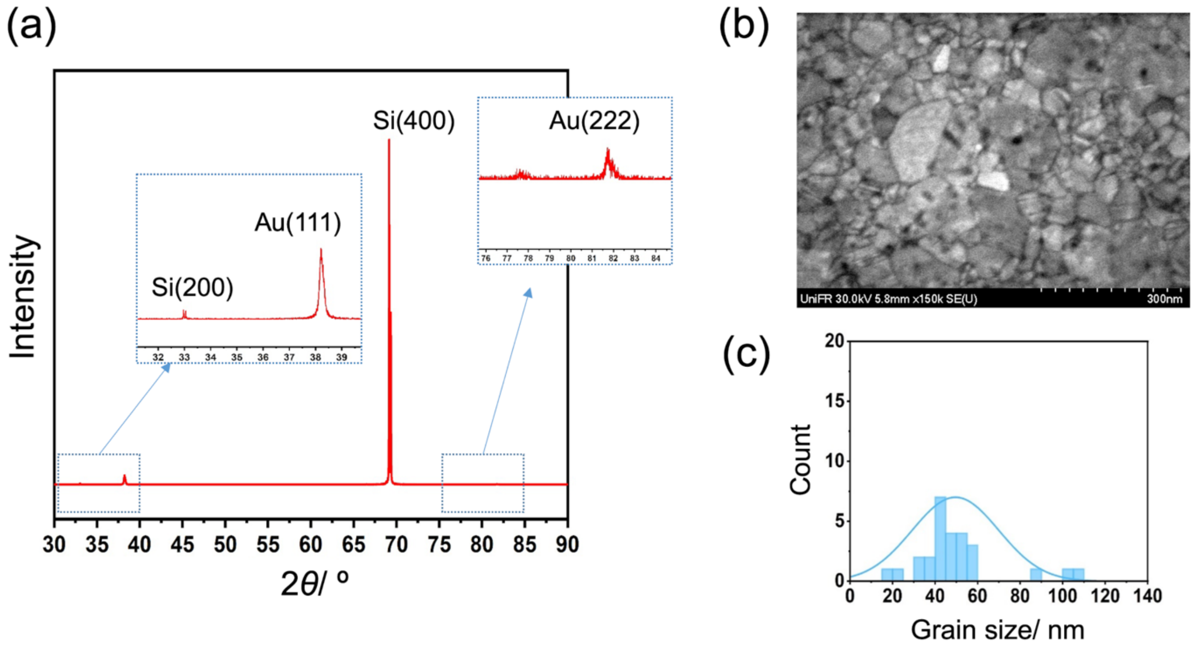

Figure S41. Characterization using a fully Au-coated substrate having a cross-sectional layer structure, as given in Fig. S1 b. (a) XRD pattern for the Au substrate: the peaks at $2\theta = 32.95^{o}$ and $2\theta = 69.14^{o}$, appear due to the Si(200) [146] and Si(400) planes, i.e., corresponding to Si-100. The peaks at $2\theta = 38.20^{o}$ and $2\theta = 81.78^{o}$ are due to Au(111) and Au(222), i.e., preferentially Au-111 is present on the Si-100 substrate. The average crystallite size calculated from the Au(111) peak is (55 ± 2) nm. (b) SEM image and (c) grain size distribution of the Au substrate according to the SEM image showing a grain size of (49 ± 20) nm, indicating the similar grain and crystallite sizes.

10. XPS of RAM-CH_2SH Immobilized on Au Substrates

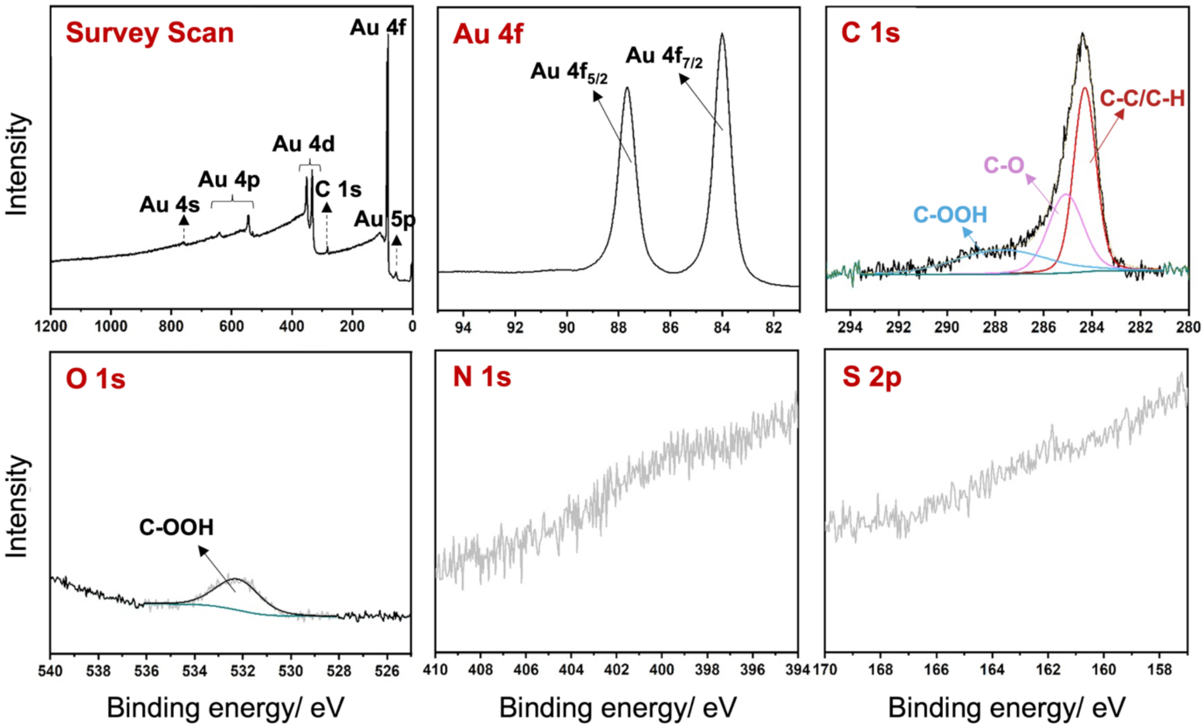

Figure S42. XPS survey scans and high-resolution (Au 4f, C 1s, O 1s, N 1s, S 2p) spectra of an Au substrate after cleaning. Au 4f peaks at 83.99 eV and 87.67 eV represent the doublet Au $4f_{7/2}$ and Au $4f_{5/2}$ with 4:3 peak area ratio and 3.68 eV of splitting energy. No shoulder toward higher binding energy states was observed. Thus, the Au(111) surface is clean and free of oxide species.[147] The C 1s signal was deconvoluted into three peaks, red (C-C/C-H: 284.30 eV), pink (C-O: 285.08 eV) and cyan (C-OOH: 287.74 eV) and O 1s peak at 532.21 eV relates to the C-OOH, hinting toward inevitable adventitious carbon. No signals were observed for N 1s and S 2p.

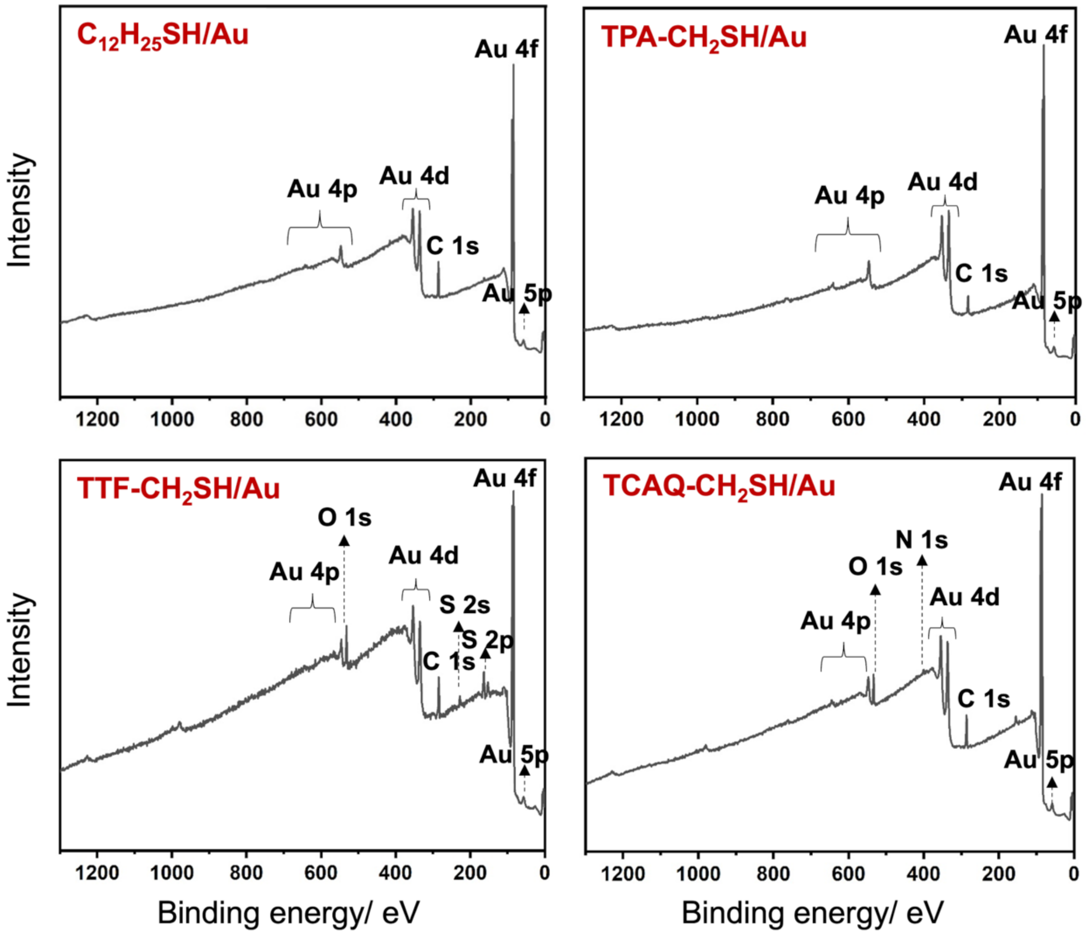

Figure S43. XPS survey scan of $C_{12}H_{25}SH$ and RAM-CH_2SH immobilized on Au substrates, respectively. The survey scans show primarily Au doublets and a C 1s peak. For the TTF-CH_2SH (**2**) and TCAQ-CH_2SH (**3**) bound to the substrate, the S and N signals are observed due to their presence in the respective compound structure. The oxygen signal here could indicate that these surfaces have high adsorption of ambient oxygen.

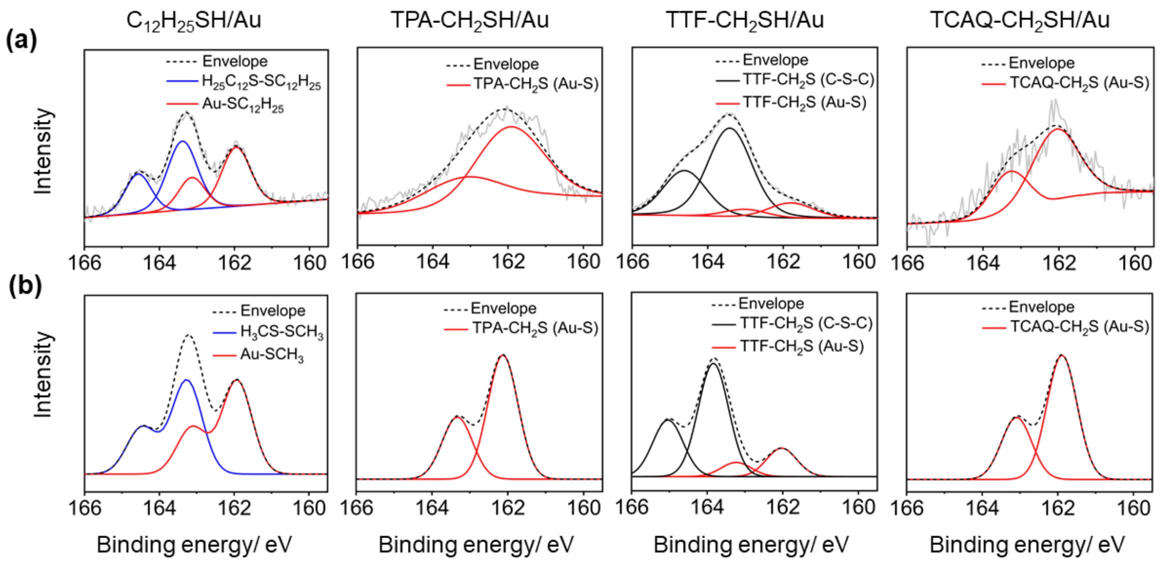

Figure S44. Comparison of (a) experimental XPS intensity vs. binding energy spectra and (b) predictions by DFT calculations. High-resolution S 2p XPS spectra for $C_{12}H_{25}SH$, TPA-CH_2SH (**1**), TTF-CH_2SH (**2**) and TCAQ-CH_2SH (**3**), immobilized on Au substrates, respectively. The red and blue color doublet peaks represent bound (Au-SCH_3) and unbound (H_3CS-SCH_3) molecules on the Au surface. The black doublet peak in the TTF-CH_2SH/Au spectrum represents C-S-C bonds. Each doublet deconvolution represents the spin-orbit coupling of the S 2p orbital, S $2p_{3/2}$ and S $2p_{1/2}$ with a peak area ratio 2:1 and a splitting energy of 1.2 eV. Theoretical spectra were folded by Gaussians with a full width half maximum value of 0.94 eV.

The S 2p spectrum for $C_{12}H_{25}SH$ reveals binding in two configurations that is bound and unbound molecules on the Au surface. The S $2p_{3/2}$ peak at 161.95 eV is due to $C_{12}H_{25}S$-Au, while the peak at 163.39 eV is due to the unbound $H_{25}C_{12}$-S-S-$C_{12}H_{25}$ molecules.[148] This is confirmed by the DFT simulations for methanethiol, where the spectra of the monomer bound to the surface and of the dimer can be identified via S $2p_{3/2}$ peaks at 161.93 eV and 163.26 eV, respectively. The simulated spectrum of the unreacted methanethiol physisorbed on the Au surface, with a S $2p_{3/2}$ peak at 162.47 eV (Fig. S36) does not fit, indicating its absence. When comparing S $2p_{3/2}$ peaks of different RAM-CH_2SH with the $C_{12}H_{25}SH$ molecule, TPA-CH_2SH (**1**), TTF-CH_2SH (**2**) and TCAQ-CH_2SH (**3**) comprise S $2p_{3/2}$ peaks centered at ca. 162 eV.

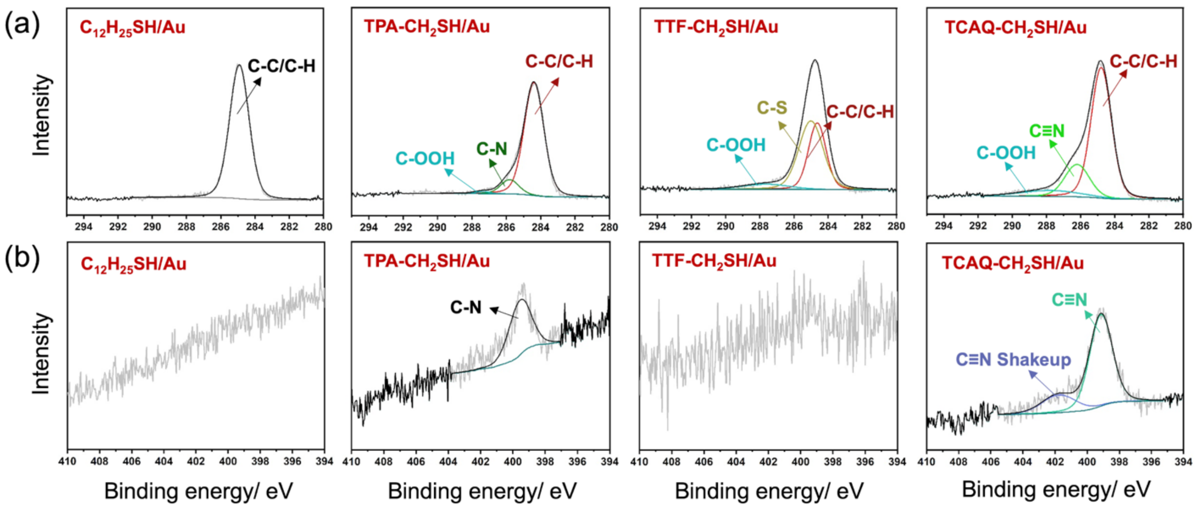

Figure S45. High-resolution XPS spectra of (a) C 1s and (b) N 1s for $C_{12}H_{25}SH$ and RAM-CH_2SH immobilized on Au substrates, respectively. The solid-colored deconvolution lines in the C 1s spectra are the fits with the application of separation energy constraints known for distinctive functional groups to the C-atoms.

In the high-resolution C 1s and N 1s core-level XPS spectra, the peak shape observed for C 1s is different in all cases representing distinctive functional groups to the C-atoms.[149] For $C_{12}H_{25}$-Au, the C 1s singlet represents 284.94 eV (C-C/C-H, aliphatic). For TPA-CH_2S-Au, the C 1s and N 1s singlets represent: 284.39 eV (C-C/C-H, aromatic), 285.50 eV (C-N), 287.39 eV (C-OOH, adventitious carbon) and 399.57 eV (C-N). For TTF-CH_2S-Au, the C 1s singlets represent: 284.64 eV (C-C/C-H, aromatic), 285.01 eV (C-S) and 287.64 eV (C-OOH, adventitious carbon). For TCAQ-CH_2S-Au, the peak centered at ca. 285 eV consists of a broad shoulder to higher binging energy, comprising multiple singlet peaks representing: 284.79 eV (C-C/C-H, aromatic), 286.20 eV (C≡N) and 287.79 eV (C-OOH, adventitious carbon). The N 1s spectra show C≡N bonds by revealing two singlet peaks at 399.15 eV and 401.77 eV. The peak at 399.15 eV relates to the four C≡N bonds with identical neighboring atoms in the structure. The peak at 401.77 eV is a satellite peak assigned to shake-ups relating to local intramolecular electronic excitation.

11. AFM Imaging and Static Contact Angles of RAM-CH_2SH Immobilized on Au

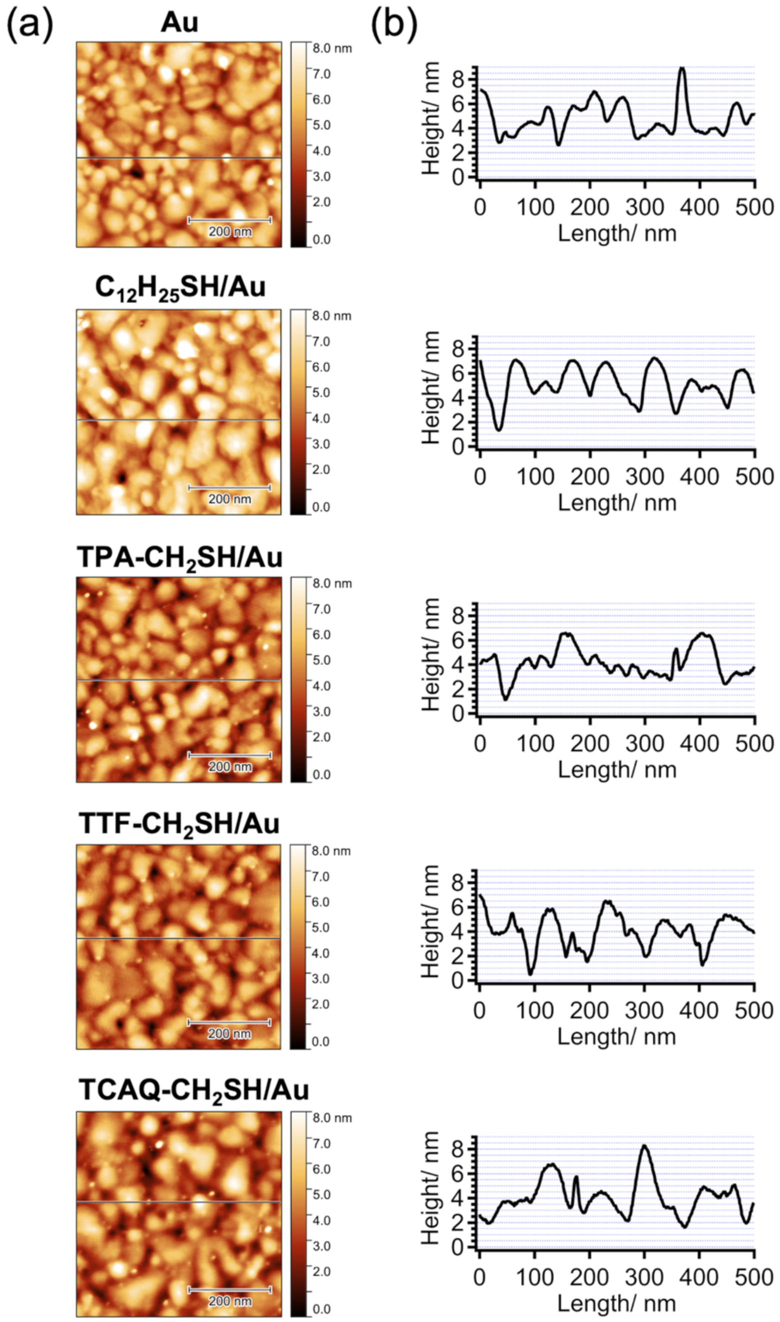

Figure S46. AFM imaging of fully Au-coated substrates. (a, b) AFM images (scale bar: 200 nm) and height profiles (along the line in a) taken in air for bare Au immediately after RCA treatment, $C_{12}H_{25}SH$/Au and RAM-CH_2SH/Au at ca. 20 °C and ca. 37% RH. The surface roughness, i.e., root mean square roughness, S_q is: Au: 1.2 nm, $C_{12}H_{25}SH$/Au: 1.4 nm, TPA-CH_2SH/Au: 1.3 nm, TTF-CH_2SH/Au: 1.2 nm, TCAQ-CH_2SH/Au: 1.3 nm. The surface roughness of the Au substrate is mostly preserved, even when functionalized, which hints toward uniform molecular layers of $C_{12}H_{25}SH$/Au and RAM-CH_2SH/Au immobilized on Au.

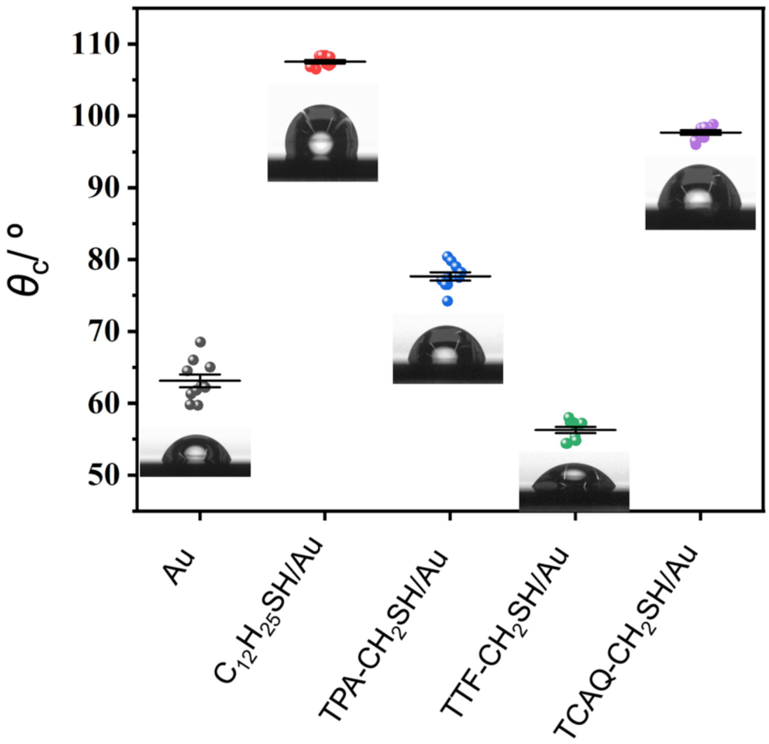

Figure S47. Static contact angles θ_c using H_2O for Au, $C_{12}H_{25}SH$ and RAM-CH_2SH immobilized on Au substrates at ca. 20 ºC and ca. 37% RH, respectively. The contact angle value for bare Au of $\theta_{c(Au)} = 61°$ is in accordance with published values of Au.[150] That also underlines the success of the presented cleaning protocol. $C_{12}H_{25}S$-Au ($\theta_{c(C12H25SH\text{-}Au)} = 107°$) is hydrophobic due to its long aliphatic chain with a terminal methyl (CH_3) group.[151] The hydro neutral character of cyano groups (CN) and the anthraquinone core renders TCAQ-CH_2S-Au ($\theta_{c(TCAQ\text{-}CH2S\text{-}Au)} = 98°$) hydrophobic.[152] TPA-$CH_2S$-Au is mildly hydrophilic ($\theta_{c(TPA\text{-}CH2S\text{-}Au)} = 79°$), most probably due to the tertiary amine connected to the three phenyl groups, and TTF-CH_2S-Au is hydrophilic ($\theta_{c(TTF\text{-}CH2S\text{-}Au)} = 57°$) because the lone pair on S in the dithiol can presumably contribute to hydrophilicity. Thus, the wettability is significantly affected by the immobilized compounds and the small error values of the contact angles θ_c depict the macroscopic uniformity and homogeneity of the bare and functionalized Au substrates.

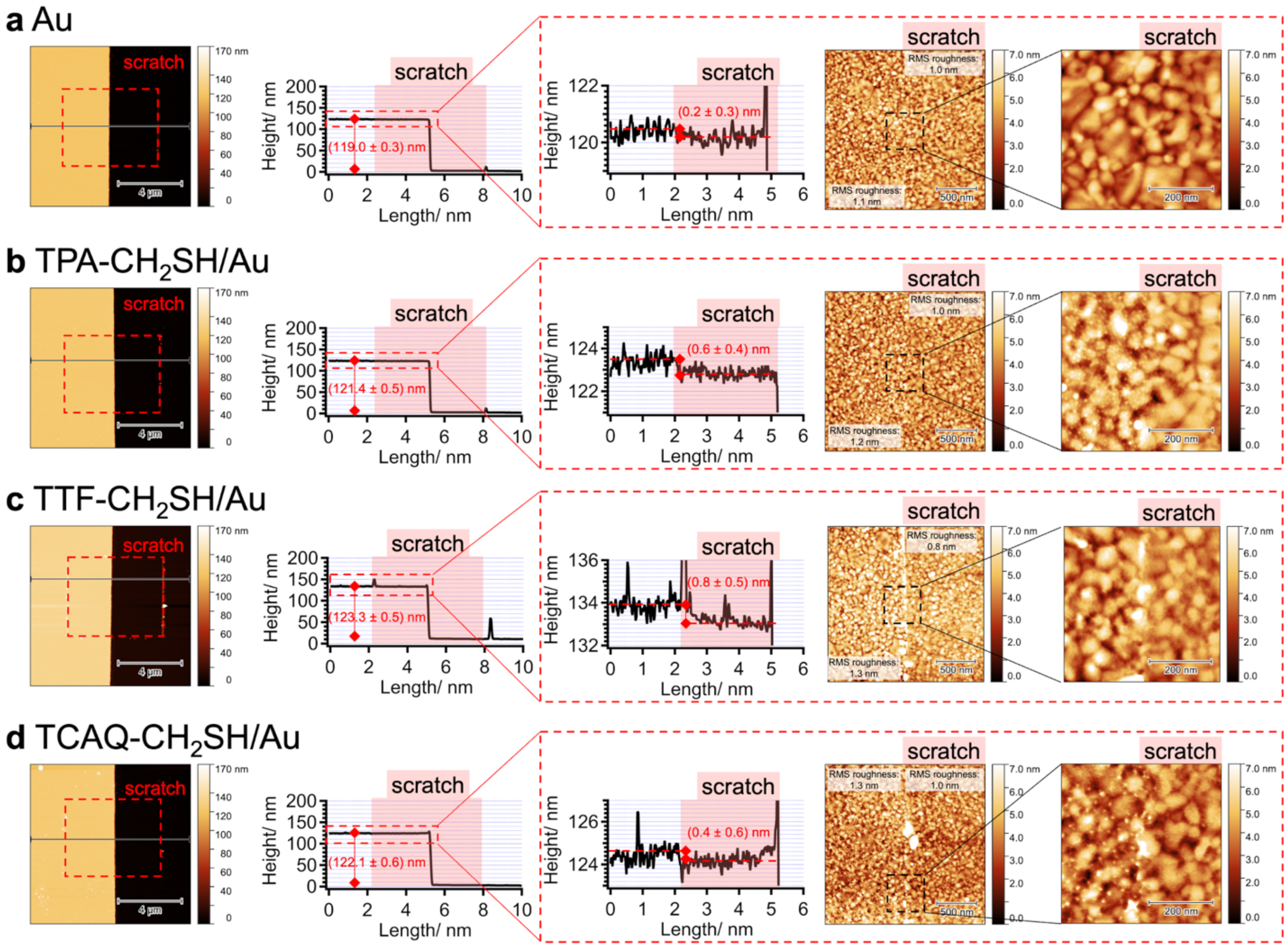

Figure S48. AFM-based scratching for RAM layer thickness and coverage determination of the RAM-functionalized CEAM substrates of (a) bare Au immediately after RCA treatment, (b) TPA-CH_2S-Au, (c) TTF-CH_2S-Au and (d) TCAQ-CH_2S-Au. AFM images (scale bar: 4 μm) of scratched areas of (5 × 5) μm^2 and height profiles (along the line indicated in the AFM images, respectively) and zoom-ins thereof. All experiments were done in air at ca. 24 ºC and ca. 37% RH.

Height profiles given in Fig. S48 were taken with a width of 20 pixels (i.e., averaging over 20 neighboring lines). This is a good compromise between averaging over several lines while not biasing the height values by possible slight tilts over the large lateral distance along the imaged surface. The height difference between scratched (highlighted in light red) and non-scratched regions is obtained by the mean of the height values in the respective region and the corresponding standard deviations taken as a measure for the error of the height difference. The RAM layer thickness values are: Au: (0.2 ± 0.3) nm, TPA-CH_2S-Au: (0.6 ± 0.4) nm, TTF-CH_2S-Au: (0.8 ± 0.5) nm and TCAQ-CH_2S-Au: (0.4 ± 0.6) nm. For bare Au we associate the obtained height difference due to scratching with removed Au atoms.[153] The difference between bare Au and RAM-functionalized Au hints toward a RAM layer, wherein the RAMs might take a flat orientation, as predicted by DFT calculations in section B5.

A qualitative impression of the coverage is obtained by the comparison of scratched and intact areas at the scale of (500 × 500) nm^2, where dot-like structured are observed in particular on the more hydrophobic samples (with TPA and TCAQ functionalization), which we interpret as adsorbates on the immobilized RAMs.[154] In addition, the roughness is higher than for the scratched regions.

12. Electrochemical Characterization of RAM-CH_2SH Immobilized Au Substrates via CV

The characterization of the bare Au electrode was performed using CV in 0.1 M *n*-Bu_4NPF_6 in anhydrous acetonitrile at a scan rate of 100 mV s^{-1}. The resulting CV curve, presented in Fig. S49, exhibits the typical features of a Au(111) surface, as previously described in the literature.[155,156] Specifically, the oxidation peaks corresponding to the formation of AuO (I_a at 0.38 V) and Au_2O_3 (II_a at 1.39 V) were observed, along with a reduction peak (I_c at 0.59 V). These characteristic features were altered upon the bonding of RAM-CH_2SH to the Au substrate. The changes in the CV profiles confirm the presence of a modified layer on the Au electrode.

For all Au substrate bound RAM-CH_2SH systems, the second anodic peak (II_a) was detected at different onset oxidation potentials, indicating interactions between RAM-CH_2SH and the Au atoms. In the case of $C_{12}H_{25}SH$, Au oxidation was largely suppressed, possibly due to the formation of a dense and tightly packed monolayer on the substrate. Among the three systems, TCAQ-CH_2SH (**3**) produced the largest shift in the onset oxidation potential (1.35 V) compared to 0.62 V of the bare Au surface, suggesting a higher interaction with Au resulting in a higher energy requirement for Au oxidation. Both TTF-CH_2SH (**2**) (0.78 V) and TPA-CH_2SH (**1**) (0.67 V) shifted the potential to more anodic values, but in lower amount compared to TCAQ-CH_2SH (**3**), showing onset oxidation potentials close to the bare Au surface.

In order to obtain an estimation of the coverage, the integral of the oxide-reduction peak Q on the reverse scan was taken within a window of 0 V to 0.85 V (Fig. S49), after subtracting a local linear baseline, and compared to the oxide-reduction peak of pure Au Q_{Au}. The blocked fraction of the surface area (covered area) suppresses the oxide formation of Au. Thus, the molecular coverage C could be estimated by: $C \approx 1 - Q\, Q_{Au}^{-1}$, obtaining: $C_{C12H25SH} \approx 0.27$, $C_{TPA\text{-}CH2SH} \approx 0.04$, $C_{TTF\text{-}CH2SH} \approx 0.28$ and $C_{TCAQ\text{-}CH2SH} \approx 0.54$. Please note that this is a very simple

estimation, in particular due to variations of the overall surface area of the Au samples used for the CV experiments of $C_{12}H_{25}SH$ and the different RAM-CH_2SH.

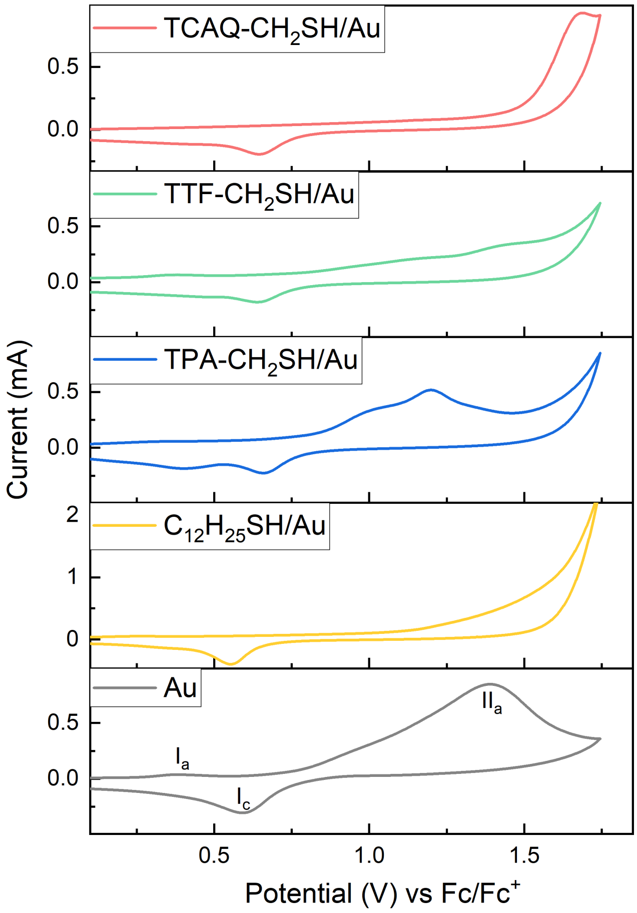

Figure S49. Cyclic voltammograms (first cycle) recorded at the bare and RAM-CH_2SH immobilized Au electrodes between 0.1 V and 1.8 V vs. Fc/Fc^+, with a scan rate of 100 mVs^{-1} in 0.1 M n-Bu_4NPF_6 in acetonitrile.

To characterize Au substrate bound RAM-CH_2SH, we avoided the oxidation of the Au surface by limiting the anodic potentials to less than 0.4 V (vs. Fc/Fc^+). A traditional method to investigate SAMs on Au electrodes involves the use of Fc.[157] This species is frequently employed to probe electrodes modified by organic thin layers due to its reversible, one-electron Fe^{2+}/Fe^{3+} redox reaction. CV measurements on freshly prepared electrodes were performed within the potential range of –0.4 to 0.4 V. As shown in Fig. S50, the presence of the $C_{12}H_{25}SH$ monolayer increased the oxidation peak potential (E_{pa}) from 50 mV (bare Au surface) to 99 mV. Thioalkanes are known to chemisorb on Au and form a well-organized, densely packed monolayers that acts as an effective barrier to electron and ion transfer,[158] thereby hindering the Fc/Fc^+ redox reaction. In the case of TPA-CH_2SH (**1**) and TCAQ-CH_2SH (**3**), the Fc/Fc^+ probe peaks were slightly shifted in potential, 49 mV and 45 mV, respectively, demonstrating that the electron transfer was slightly improved by the presence of the monolayers. The TTF-CH_2SH (**2**) monolayer showed the lowest E_{ox} (37 mV), indicating that the modification of the Au surface with this molecule can improve the electron transfer of the Fc/Fc^+ couple.

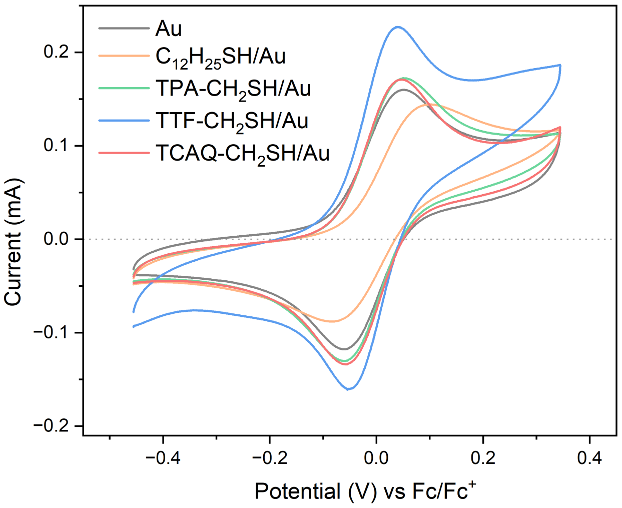

Figure S50. Cyclic voltammograms recorded on bare and RAM-CH_2SH immobilized Au electrodes with a scan rate of 100 mVs^{-1} in 0.1 M n-Bu_4NPF_6 in acetonitrile and 5 mM of Fc.

13. CEAM Assay

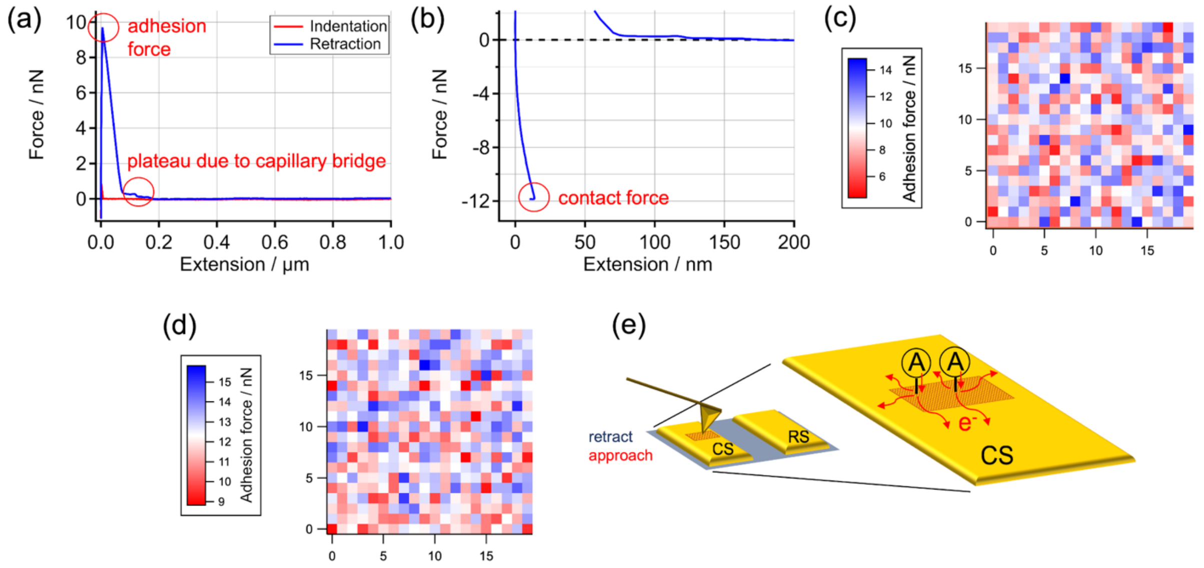

Figure S51. Adhesion peak and contact force evaluation, taking the example of Au_T-Au_S. (a) Indentation (in red) and retraction (in blue) portions of the force-extension curve. The maximum force of the adhesion peak (adhesion force, $F_{adhesion}$) was obtained from the retraction portion of the force-extension curve. Plateaus of constant force are most probably due to capillary bridges between the Au tip and the Au substrate in N_2 (22°C, 14% RH), as discussed in the literature.[159] (b) Retraction portion of the force-extension curve showing the contact phase (below the zero line) and the contact force (trigger force, $F_{contact}$), where the retraction motion starts. $F_{contact}$ values for different (functionalized) tip - substrate combinations are given Table S5. (c, d) $F_{adhesion}$ values of a force map across an area of (5 × 5) μm^2 comprising 20 × 20 force-extension curves (400 contacts) from which the mean and the standard deviation were taken, exemplarily for the case of (c) T_{Au}-S_{Au} and (d) T_{TTF}-S_{TCAQ}, both measured in N_2. $F_{adhesion}$ values for different (functionalized) tip - substrate combinations are given in Table S5. (e) Illustration of the lateral diffusion of the charge transferred due to the contact-separation process (based on the situation for the acceptor bound to the Au stripe, as for S_{TCAQ}, with electrons as charge carriers) in accordance with the Hirshfeld analysis (Table S4).

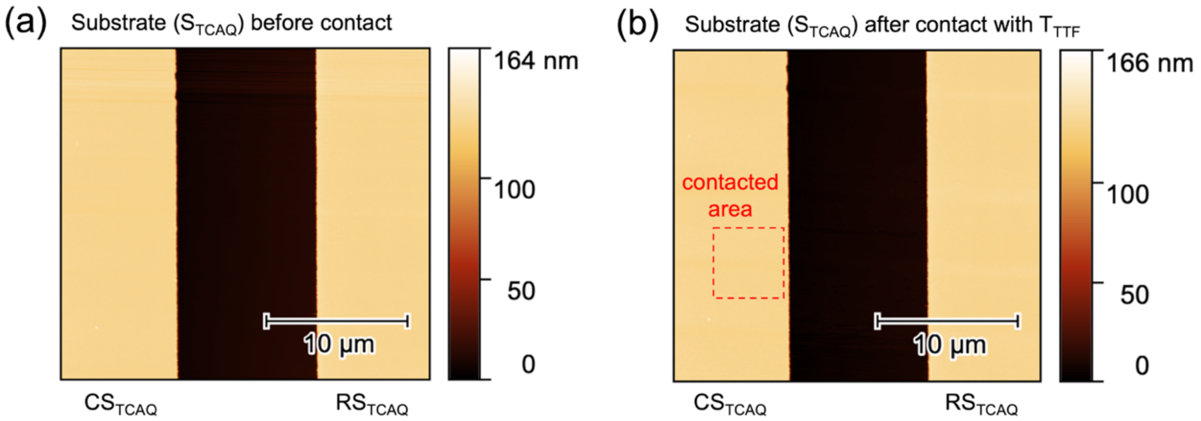

Figure S52. Exemplary AFM topography image of a CEAM substrate, S_{TCAQ} in N_2 (22 °C, 14% RH), before and after contact with a functionalized AFM cantilever tip, T_{TTF}. No material transfer could be observed with respect to the topography at the scale of our experiment.

Table S5. Contact-separation via force spectroscopy for different (functionalized) tip (T)- substrate combinations (up to three sets each, wherein each one was taken on a new pair of Au stripes (S) of the CEAM substrate): mean and error values (standard deviation, quadratic error propagation) of contact force $F_{contact}$, adhesion force $F_{adhesion}$, contact area a_{DMT}, contact pressure p_{DMT} (Eqns. S7 to S9), mean change of surface potential $\overline{\Delta V}_{CPD}$ and mean surface-charge-density change $\overline{\Delta\sigma}$ (Eqn. S12). The real contact time (< 5.1 s) comprises the dwell time (5.0 s), the indentation time (ca. 50 ms) and the retraction time (max. 20 ms). Values marked by a star represent force maps that also comprise data points from the SiO_2 part of the CEAM substrate (i.e., force map covers both Au stripe and SiO_2 background). Conditions: N_2: 22°C, 14% RH, ambient: 22°C, 37% RH. Step (iii) was usually performed with a time delay of ca. 40 min after the final force-extension curve of step (ii).

**Tip-substrate combination**	$F_{contact}$/ nN	$F_{adhesion}$/ nN	a_{DMT}/ nm^2	p_{DMT}/ GPa	$\overline{\Delta V}_{CPD}$/ V	$\overline{\Delta\sigma}$/ e (100 nm)$^{-2}$	$\overline{\langle\Delta\sigma\rangle}$/ e (100 nm)$^{-2}$
T_{Au}-S_{Au}/ N_2	12.01 ± 0.08	9.6 ± 1.9	15.4 ± 0.4	1.4 ± 0.1	0.06 ± 0.03	0.3 ± 0.2	0.2 ± 0.4
	11.89 ± 0.15	9.1 ± 1.0	15.1 ± 0.2	1.4 ± 0.1	0 ± 0.07	0 ± 0.4	
T_{TPA}-S_{TCAQ}/ N_2	11.00 ± 0.10	10.8 ± 0.9	15.4 ± 0.2	1.4 ± 0.1	0.16 ± 0.05	0.8 ± 0.3	
	11.97 ± 0.05	10.9 ± 0.9	16.0 ± 0.2	1.4 ± 0.1	0.27 ± 0.05	1.4 ± 0.3	1.0 ± 0.4
	10.98 ± 0.07	13.8 ± 1.9*	16.8 ± 0.4	1.5 ± 0.1	0.19 ± 0.03	0.9 ± 0.2	
T_{TTF}-S_{TCAQ}/ N_2	12.43 ± 0.21	7.3 ± 0.8	14.5 ± 0.2	1.4 ± 0.1	0.89 ± 0.09	4.4 ± 0.5	
	11.35 ± 0.07	12.3 ± 1.3	16.3 ± 0.3	1.5 ± 0.1	0.70 ± 0.15	3.5 ± 0.8	4.0 ± 1.4
	10.69 ± 0.07	17.3 ± 1.8*	18.3 ± 0.3	1.5 ± 0.1	0.80 ± 0.20	4.0 ± 1.1	
T_{Au}-S_{TCAQ}/ N_2	11.39 ± 0.10	9.3 ± 1.3	14.9 ± 0.3	1.4 ± 0.1	0.53 ± 0.07	2.6 ± 0.4	
	12.26 ± 0.10	10.2 ± 0.5	15.8 ± 0.1	1.4 ± 0.1	0.17 ± 0.03	0.9 ± 0.2	1.5 ± 0.4
	11.68 ± 0.08	10.2 ± 0.5	15.5 ± 0.1	1.4 ± 0.1	0.22 ± 0.03	1.1 ± 0.2	
T_{TCAQ}-S_{TPA}/ N_2	10.64 ± 0.10	6.2 ± 2.4*	13.0 ± 0.6	1.3 ± 0.2	-0.01 ± 0.02	-0.1 ± 0.1	
	10.84 ± 0.05	9.5 ± 2.7	14.7 ± 0.6	1.4 ± 0.2	-0.07 ± 0.03	-0.4 ± 0.2	-0.2 ± 0.4
	10.45 ± 0.11	9.8 ± 3.1	14.7 ± 0.7	1.4 ± 0.2	0.07 ± 0.06	0.4 ± 0.3	
T_{TCAQ}-S_{TTF}/ N_2	10.51 ± 0.12	9.0 ± 1.2	14.4 ± 0.3	1.4 ± 0.1	-0.17 ± 0.06	-0.9± 0.3	
	10.86 ± 0.16	10.0 ± 0.7	15.0 ± 0.2	1.4 ± 0.1	-0.33 ± 0.06	-1.6 ± 0.3	-1.3 ± 0.6
	10.62 ± 0.22	11.6 ± 2.1*	15.7 ± 0.4	1.4 ± 0.1	-0.25 ± 0.07	-1.3 ± 0.4	
T_{TCAQ}-S_{Au}/ N_2	11.67 ± 0.12	8.5 ± 1.9*	14.7 ± 0.4	1.4 ± 0.1	-0.01 ± 0.11	-0.1 ± 0.6	
	11.90 ± 0.09	9.6 ± 2.3*	15.3 ± 0.5	1.4 ± 0.2	-0.03 ± 0.06	-0.2 ± 0.3	-0.4 ± 0.7
	13.99 ± 0.27	6.1 ± 1.7	14.6 ± 0.4	1.4 ± 0.1	-0.18 ± 0.07	-0.9 ± 0.4	
T_{TPA}-S_{TCAQ}/ ambient	10.16 ± 0.17	13.28 ± 2.5	16.2 ± 0.5	1.4 ± 0.2	-0.06 ± 0.15	-0.3 ± 0.8	-
T_{TTF}-S_{TCAQ}/ ambient	10.00 ± 0.05	12.55 ± 1.8	15.8 ± 0.4	1.4 ± 0.1	-0.25 ± 0.33	-1.2 ± 1.6	-

14. CEAM Assay: Time-Dependence of Charge Dissipation after Charge-Separation Process

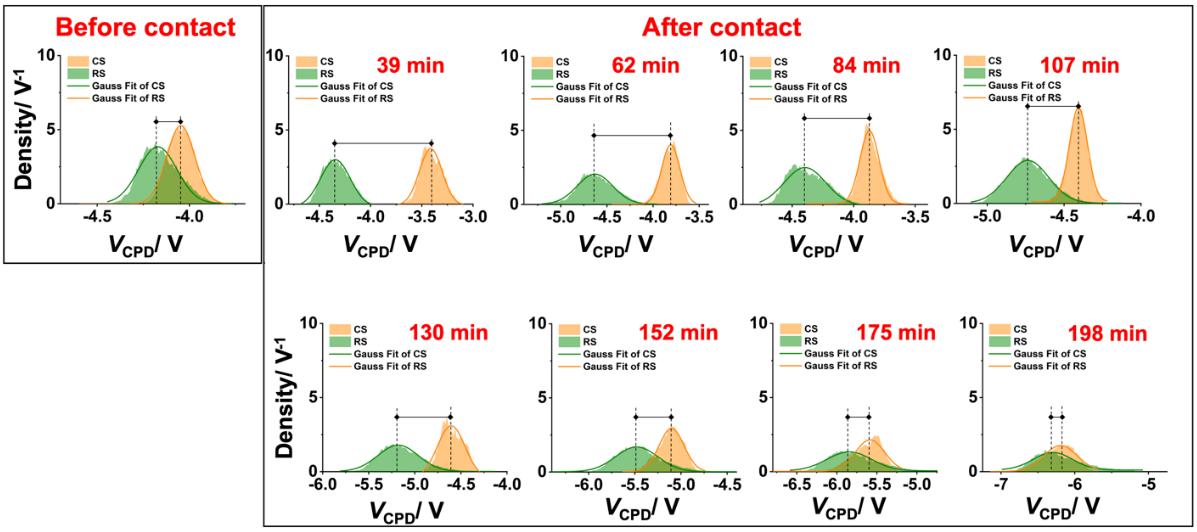

Figure S53. Time-dependence of the charge dissipation for T_{TTF}-CS_{TCAQ} in N_2 (22°C, 14% RH). The V_{CPD} distributions $\varrho(V_{CPD})$ of CS and RS are given at time points before and after contact-separation process. The given times represent the end of the KPFM image acquisition process for each image. A clear time-dependent decrease of $\bar{V}_{CPD,\text{after contact}}$ (given by the difference of the mean values indicated by the dashed lines) is observed, i.e., a time-dependent decay of $\overline{\Delta V}_{CPD}$ is revealed.

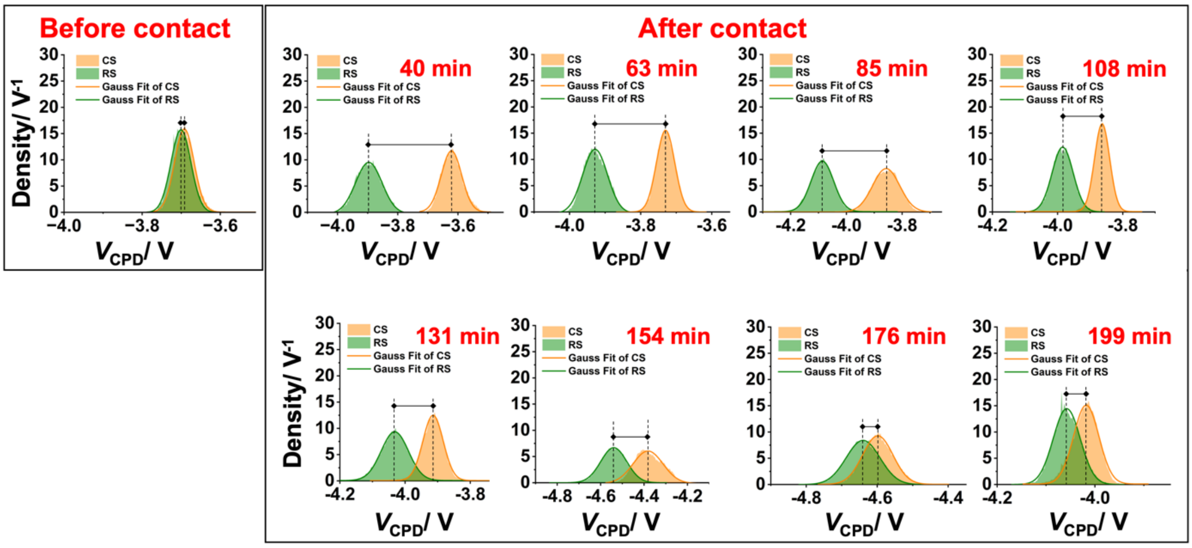

Figure S54. Time-dependence of the charge dissipation for T_{TPA}-CS_{TCAQ} in N_2 (22°C, 14% RH), analogous to Fig. S53. Again, a clear decrease of $\bar{V}_{CPD,\text{after contact}}$ is observed, i.e., a time-dependent decay of $\overline{\Delta V}_{CPD}$ is revealed.

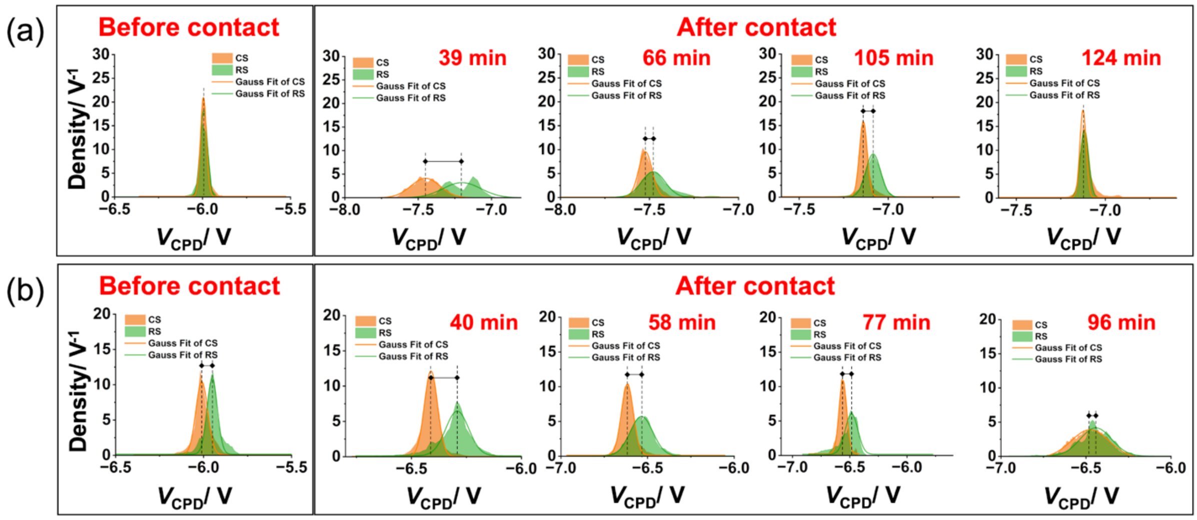

Figure S55. Time-dependence of the charge dissipation for (a) T_{TTF}-CS_{TCAQ} and (b) T_{TPA}-CS_{TCAQ} under ambient conditions (22 °C, 37% RH). The measurements were done analogous to Figs. S53 and S54. A slight time-dependent increase of $\bar{V}_{CPD,\text{after contact}}$ (given by the difference of the mean values indicated by the dashed lines) is observed, i.e., a time-dependent decay of $\overline{\Delta V}_{CPD}$ is revealed.

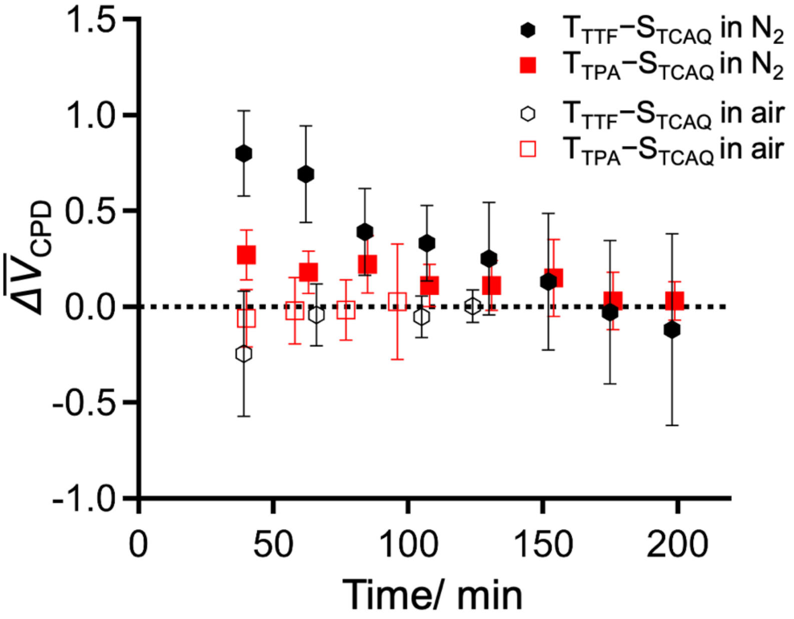

Figure S56. Comparison of the time-dependence of the charge dissipation for T_{TTF}-S_{TCAQ} and T_{TPA}-CS_{TCAQ} under N_2 conditions (22 °C, 14% RH, see Fig. 4 c) and ambient conditions (22 °C, 37% RH, see Fig. S55), respectively. Under N_2 conditions, an exponential decrease of $\overline{\Delta V}_{CPD}$ is observed, while under ambient conditions possibly a surface potential reversal is observed,[81] having a much lower $\overline{\Delta V}_{CPD}$ magnitude than in N_2, which quickly decays to 0. Please note that the error bars are large under ambient conditions, both for T_{TTF}-S_{TCAQ} and T_{TPA}-CS_{TCAQ}.

C. References

[1] D. J. Lacks, T. Shinbrot, *Nat. Rev. Chem.* **2019**, *3*, 465–476.

[2] H. Zou, Y. Zhang, L. Guo, P. Wang, X. He, G. Dai, H. Zheng, C. Chen, A. C. Wang, C. Xu, Z. L. Wang, *Nat. Commun.* **2019**, *10*, 1427.

[3] S. Pan, Z. Zhang, *Friction* **2019**, *7*, 2–17.

[4] D. J. Lacks, R. Mohan Sankaran, *J. Phys. Appl. Phys.* **2011**, *44*, 453001.

[5] J. Lowell, A. C. Rose-Innes, *Adv. Phys.* **1980**, *29*, 947–1023.

[6] D. J. Lacks, *Angew. Chem. Int. Ed.* **2012**, *51*, 6822–6823.

[7] J. Henniker, *Nature* **1962**, *196*, 474–474.

[8] G. Fatti, H. Kim, C. Sohn, M. Park, Y. Lim, Z. Li, K.-I. Park, I. Szlufarska, H. Ko, C. K. Jeong, S. B. Cho, *Phys. Rev. Lett.* **2023**, *131*, 166201.

[9] H. Qiao, P. Zhao, O. Kwon, A. Sohn, F. Zhuo, D. Lee, C. Sun, D. Seol, D. Lee, S. Kim, Y. Kim, *Adv. Sci.* **2021**, *8*, 2101793.

[10] J. Zhang, M. L. Coote, S. Ciampi, *J. Am. Chem. Soc.* **2021**, *143*, 3019–3032.

[11] H. T. Baytekin, A. Z. Patashinski, M. Branicki, B. Baytekin, S. Soh, B. A. Grzybowski, *Science* **2011**, *333*, 308–312.

[12] M. M. Apodaca, P. J. Wesson, K. J. M. Bishop, M. A. Ratner, B. A. Grzybowski, *Angew. Chem. Int. Ed.* **2010**, *49*, 946–949.

[13] J. C. Sobarzo, F. Pertl, D. M. Balazs, T. Costanzo, M. Sauer, A. Foelske, M. Ostermann, C. M. Pichler, Y. Wang, Y. Nagata, M. Bonn, S. Waitukaitis, *Nature* **2025**, *638*, 664–669.

[14] M. Kaponig, A. Mölleken, H. Nienhaus, R. Möller, *Sci. Adv.* **2021**, *7*, eabg7595.

[15] T. Shinbrot, B. Ferdowsi, S. Sundaresan, N. A. M. Araujo, *Phys. Rev. Mater.* **2018**, *2*, 125003.

[16] Y. Nurmakanov, G. Kalimuldina, G. Nauryzbayev, D. Adair, Z. Bakenov, *Nanoscale Res. Lett.* **2021**, *16*, 122.

[17] C.-Y. Chang, J.-R. Yang, Y.-S. Liu, A. Panda, *Mater. Horiz.* **2024**, *11*, 646–660.

[18] S. Wang, Y. Zi, Y. S. Zhou, S. Li, F. Fan, L. Lin, Z. L. Wang, *J. Mater. Chem. A* **2016**, *4*, 3728–3734.

[19] Y. Cheng, C. Lee, C. Chang, *Adv. Mater. Technol.* **2021**, *6*, 2000985.

[20] S. Mishra, P. Supraja, D. Haranath, R. R. Kumar, S. Pola, *Nano Energy* **2022**, *104*, 107964.

[21] I. Aazem, R. Walden, A. Babu, S. C. Pillai, *Results Eng.* **2022**, *16*, 100756.

[22] M. Ibrahim, J. Jiang, Z. Wen, X. Sun, *Nanoenergy Adv.* **2021**, *1*, 58–80.

[23] M. Nonnenmacher, M. O'Boyle, H. K. Wickramasinghe, *Ultramicroscopy* **1992**, *42–44*, 268–273.

[24] U. Zerweck, C. Loppacher, T. Otto, S. Grafström, L. M. Eng, *Phys. Rev. B* **2005**, *71*, 125424.

[25] M. Geisler, T. Hugel, *Adv. Mater.* **2010**, *22*, 398–402.

[26] H. Lee, W. Lee, J. H. Lee, D. S. Yoon, *J. Nanomater.* **2016**, *2016*, 1–21.

[27] A. Axt, I. M. Hermes, V. W. Bergmann, N. Tausendpfund, S. A. L. Weber, *Beilstein J. Nanotechnol.* **2018**, *9*, 1809–1819.

[28] Y. S. Zhou, Y. Liu, G. Zhu, Z.-H. Lin, C. Pan, Q. Jing, Z. L. Wang, *Nano Lett.* **2013**, *13*, 2771–2776.

[29] S. Im, E. Frey, D. J. Lacks, J. Genzer, M. D. Dickey, *Adv. Sci.* **2023**, *10*, 2304459.

[30] I. S. M. Jimidar, W. Kwiecinski, G. Roozendaal, E. S. Kooij, H. J. G. E. Gardeniers, G. Desmet, K. Sotthewes, *ACS Appl. Mater. Interfaces* **2023**, *15*, 42004–42014.

[31] T. Mukai, T. Suzuki, Y. Yamashita, *Bull. Chem. Soc. Jpn.* **1985**, *58*, 2433–2434.

[32] R. Otero, A. L. Vázquez De Parga, J. M. Gallego, *Surf. Sci. Rep.* **2017**, *72*, 105–145.

[33] K. Xu, H. Sun, T.-P. Ruoko, G. Wang, R. Kroon, N. B. Kolhe, Y. Puttisong, X. Liu, D. Fazzi, K. Shibata, C.-Y. Yang, N. Sun, G. Persson, A. B. Yankovich, E. Olsson, H. Yoshida, W. M. Chen, M. Fahlman, M. Kemerink, S. A. Jenekhe, C. Müller, M. Berggren, S. Fabiano, *Nat. Mater.* **2020**, *19*, 738–744.

[34] D. Kiefer, R. Kroon, A. I. Hofmann, H. Sun, X. Liu, A. Giovannitti, D. Stegerer, A. Cano, J. Hynynen, L. Yu, Y. Zhang, D. Nai, T. F. Harrelson, M. Sommer, A. J. Moulé, M. Kemerink, S. R. Marder, I. McCulloch, M. Fahlman, S. Fabiano, C. Müller, *Nat. Mater.* **2019**, *18*, 149–155.

[35] C. Mathur, R. Gupta, R. K. Bansal, *Chem. – Eur. J.* **2024**, *30*, e202304139.

[36] P. Cias, C. Slugovc, G. Gescheidt, *J. Phys. Chem. A* **2011**, *115*, 14519–14525.

[37] H. Alves, A. S. Molinari, H. Xie, A. F. Morpurgo, *Nat. Mater.* **2008**, *7*, 574–580.

[38] Q. Zhou, K. Song, G. Zhang, X. Song, J. Lin, Y. Zang, D. Zhang, D. Zhu, *Nat. Commun.* **2022**, *13*, 1803.

[39] W. Shi, M. Tang, W. Deng, P. Li, X. Yang, H. Huang, P. Du, J. Liu, C. Ming Li, *J. Colloid Interface Sci.* **2022**, *607*, 1173–1179.

[40] Y. Satake, H. Fujihara, *Electrochemistry* **2005**, *73*, 38–40.

[41] B. Esser, *Org. Mater.* **2019**, *01*, 063–070.

[42] John. Ferraris, D. O. Cowan, V. Walatka, J. H. Perlstein, *J. Am. Chem. Soc.* **1973**, *95*, 948–949.

[43] T. Dadosh, Y. Gordin, R. Krahne, I. Khivrich, D. Mahalu, V. Frydman, J. Sperling, A. Yacoby, I. Bar-Joseph, *Nature* **2005**, *436*, 677–680.

[44] S. Creager, C. J. Yu, C. Bamdad, S. O'Connor, T. MacLean, E. Lam, Y. Chong, G. T. Olsen, J. Luo, M. Gozin, J. F. Kayyem, *J. Am. Chem. Soc.* **1999**, *121*, 1059–1064.

[45] A. W. Franz, S. Stoycheva, M. Himmelhaus, T. J. J. Müller, *Beilstein J. Org. Chem.* **2010**, *6,* 72.

[46] A. Nitzan, M. A. Ratner, *Science* **2003**, *300*, 1384–1389.

[47] J. Liu, Z. Yang, S. Li, Y. Du, Z. Zhang, J. Shao, M. Willatzen, Z. L. Wang, D. Wei, *J. Am. Chem. Soc.* **2024**, *146*, 31574–31584.

[48] S. Li, Z. Zhang, P. Peng, X. Li, Z. L. Wang, D. Wei, *Nano Energy* **2024**, *122*, 109286.

[49] Z. Wang, A. Berbille, Y. Feng, S. Li, L. Zhu, W. Tang, Z. L. Wang, *Nat. Commun.* **2022**, *13*, 130.

[50] F. Brunel, C. Lautard, F. Garzino, S. Giorgio, J. M. Raimundo, J. M. Bolla, M. Camplo, *Bioorg. Med. Chem. Lett.* **2016**, *26*, 3770–3773.

[51] J. Garín, J. Orduna, S. Uriel, A. J. Moore, M. R. Bryce, S. Wegener, D. S. Yufit, J. A. K. Howard, *Synthesis* **1994**, *1994*, 489–493.

[52] R. Yuge, A. Miyazaki, T. Enoki, K. Tamada, F. Nakamura, M. Hara, *J. Phys. Chem. B* **2002**, *106*, 6894–6901.

[53] Y. Hou, P. Wan, *Photochem. Photobiol. Sci.* **2008**, *7*, 588–596.

[54] A. P. Brogan, W. R. Widger, H. Kohn, *J. Org. Chem.* **2003**, *68*, 5575–5587.

[55] J. J. Mortensen, A. H. Larsen, M. Kuisma, A. V. Ivanov, A. Taghizadeh, A. Peterson, A. Haldar, A. O. Dohn, C. Schäfer, E. Ö. Jónsson, E. D. Hermes, F. A. Nilsson, G. Kastlunger, G. Levi, H. Jónsson, H. Häkkinen, J. Fojt, J. Kangsabanik, J. Sødequist, J. Lehtomäki, J. Heske, J. Enkovaara, K. T. Winther, M. Dulak, M. M. Melander, M. Ovesen, M. Louhivuori, M. Walter, M. Gjerding, O. Lopez-Acevedo, P. Erhart, R. Warmbier, R. Würdemann, S. Kaappa, S. Latini, T. M. Boland, T. Bligaard, T. Skovhus, T. Susi, T. Maxson, T. Rossi, X. Chen, Y. L. A. Schmerwitz, J. Schiøtz, T. Olsen, K. W. Jacobsen, K. S. Thygesen, *J. Chem. Phys.* **2024**, *160*, 092503.

[56] J. P. Perdew, K. Burke, M. Ernzerhof, *Phys. Rev. Lett.* **1996**, *77*, 3865–3868.

[57] A. Held, M. Walter, *J. Chem. Phys.* **2014**, *141*, 174108.

[58] J. B. Neaton, M. S. Hybertsen, S. G. Louie, *Phys. Rev. Lett.* **2006**, *97*, 216405.
[59] J. M. Garcia-Lastra, C. Rostgaard, A. Rubio, K. S. Thygesen, *Phys. Rev. B* **2009**, *80*, 245427.
[60] B. Dandrade, S. Datta, S. Forrest, P. Djurovich, E. Polikarpov, M. Thompson, *Org. Electron.* **2005**, *6*, 11–20.
[61] J. I. Martínez, E. Abad, C. González, J. Ortega, F. Flores, *Org. Electron.* **2012**, *13*, 399–408.
[62] M. Walter, M. Moseler, L. Pastewka, *Phys. Rev. B* **2016**, *94*, 041112.
[63] H. O. Jacobs, P. Leuchtmann, O. J. Homan, A. Stemmer, *J. Appl. Phys.* **1998**, *84*, 1168–1173.
[64] G. Elias, T. Glatzel, E. Meyer, A. Schwarzman, A. Boag, Y. Rosenwaks, *Beilstein J. Nanotechnol.* **2011**, *2*, 252–260.
[65] H. B. Michaelson, *J. Appl. Phys.* **1977**, *48*, 4729–4733.
[66] N. Turetta, F. Sedona, A. Liscio, M. Sambi, P. Samorì, *Adv. Mater. Interfaces* **2021**, *8*, 2100068.
[67] E. Villarreal, G. G. Li, Q. Zhang, X. Fu, H. Wang, *Nano Lett.* **2017**, *17*, 4443–4452.
[68] N. Knorr, S. Vinzelberg, *Microsc Microanal* **2012**, *26*, 7–12.
[69] F. Pertl, J. C. Sobarzo, L. Shafeek, T. Cramer, S. Waitukaitis, *Phys. Rev. Mater.* **2022**, *6*, 125605.
[70] Y. Liu, J. Mo, Q. Fu, Y. Lu, N. Zhang, S. Wang, S. Nie, *Adv. Funct. Mater.* **2020**, *30*, 2004714.
[71] D. Choi, Y. Lee, Z.-H. Lin, S. Cho, M. Kim, C. K. Ao, S. Soh, C. Sohn, C. K. Jeong, J. Lee, M. Lee, S. Lee, J. Ryu, P. Parashar, Y. Cho, J. Ahn, I.-D. Kim, F. Jiang, P. S. Lee, G. Khandelwal, S.-J. Kim, H. S. Kim, H.-C. Song, M. Kim, J. Nah, W. Kim, H. G. Menge, Y. T. Park, W. Xu, J. Hao, H. Park, J.-H. Lee, D.-M. Lee, S.-W. Kim, J. Y. Park, H. Zhang, Y. Zi, R. Guo, J. Cheng, Z. Yang, Y. Xie, S. Lee, J. Chung, I.-K. Oh, J.-S. Kim, T. Cheng, Q. Gao, G. Cheng, G. Gu, M. Shim, J. Jung, C. Yun, C. Zhang, G. Liu, Y. Chen, S. Kim, X. Chen, J. Hu, X. Pu, Z. H. Guo, X. Wang, J. Chen, X. Xiao, X. Xie, M. Jarin, H. Zhang, Y.-C. Lai, T. He, H. Kim, I. Park, J. Ahn, N. D. Huynh, Y. Yang, Z. L. Wang, J. M. Baik, D. Choi, *ACS Nano* **2023**, *17*, 11087–11219.
[72] C. Callaty, C. Rodrigues, J. Ventura, *Nano Energy* **2025**, *135*, 110661.
[73] S.-H. Shin, Y. E. Bae, H. K. Moon, J. Kim, S.-H. Choi, Y. Kim, H. J. Yoon, M. H. Lee, J. Nah, *ACS Nano* **2017**, *11*, 6131–6138.
[74] Y.-T. Jao, P.-K. Yang, C.-M. Chiu, Y.-J. Lin, S.-W. Chen, D. Choi, Z.-H. Lin, *Nano Energy* **2018**, *50*, 513–520.
[75] P. S. Gil, D. J. Lacks, *Phys. Chem. Chem. Phys.* **2019**, *21*, 13821–13825.
[76] X. Bai, A. Riet, S. Xu, D. J. Lacks, H. Wang, *J. Phys. Chem. C* **2021**, *125*, 11677–11686.
[77] J. Yin, B. Nysten, *J. Electrost.* **2018**, *96*, 16–22.
[78] C. Yun, S.-H. Lee, J. Ryu, K. Park, J.-W. Jang, J. Kwak, S. Hwang, *J. Am. Chem. Soc.* **2018**, *140*, 14687–14695.
[79] S. G. J. Mathijssen, M. Kemerink, A. Sharma, M. Cölle, P. A. Bobbert, R. A. J. Janssen, D. M. de Leeuw, *Adv. Mater.* **2008**, *20*, 975–979.
[80] H. Zhang, S. Sundaresan, M. A. Webb, *Nat. Commun.* **2024**, *15*, 2616.
[81] C. Yun, S.-H. Lee, J. Ryu, K. Park, J.-W. Jang, J. Kwak, S. Hwang, *J. Am. Chem. Soc.* **2018**, *140*, 14687–14695.
[82] M. Luna, J. Colchero, A. Gil, J. Gómez-Herrero, A. M. Baró, *Appl. Surf. Sci.* **2000**, *157*, 393–397.
[83] A. Gil, J. Colchero, J. Gómez-Herrero, A. M. Baró, *Ultramicroscopy* **2001**, *86*, 1–9.
[84] A. Verdaguer, G. M. Sacha, H. Bluhm, M. Salmeron, *Chem. Rev.* **2006**, *106*, 1478–

1510.
[85] D. Stacchiola, J. B. Park, P. Liu, S. Ma, F. Yang, D. E. Starr, E. Muller, P. Sutter, J. Hrbek, *J. Phys. Chem. C* **2009**, *113*, 15102–15105.
[86] J.-J. Velasco-Velez, C. H. Wu, T. A. Pascal, L. F. Wan, J. Guo, D. Prendergast, M. Salmeron, *Science* **2014**, *346*, 831–834.
[87] O. Knoblauch, *Z. Für Phys. Chem.* **1902**, *39U*, 225–244.
[88] G. Dubey, G. P. Lopinski, F. Rosei, *Appl. Phys. Lett.* **2007**, *91*, 232111.
[89] J. Yin, B. Nysten, *J. Electrost.* **2018**, *96*, 16–22.
[90] P. S. Gil, D. J. Lacks, *Phys. Chem. Chem. Phys.* **2019**, *21*, 13821–13825.
[91] X. Bai, A. Riet, S. Xu, D. J. Lacks, H. Wang, *J. Phys. Chem. C* **2021**, *125*, 11677–11686.
[92] Y. S. Zhou, Y. Liu, G. Zhu, Z.-H. Lin, C. Pan, Q. Jing, Z. L. Wang, *Nano Lett.* **2013**, *13*, 2771–2776.
[93] H. R. Carlon, *J. Appl. Phys.* **1981**, *52*, 2638–2641.
[94] T. R. D. Ducati, L. H. Simões, F. Galembeck, *Langmuir* **2010**, *26*, 13763–13766.
[95] H. Sugimura, Y. Ishida, K. Hayashi, O. Takai, N. Nakagiri, *Appl. Phys. Lett.* **2002**, *80*, 1459–1461.
[96] A. Liscio, V. Palermo, K. Müllen, P. Samorì, *J. Phys. Chem. C* **2008**, *112*, 17368–17377.
[97] Z. L. Wang, *ACS Nano* **2013**, *7*, 9533–9557.
[98] Z. L. Wang, *Nano Energy* **2020**, *68*, 104272.
[99] W.-G. Kim, D.-W. Kim, I.-W. Tcho, J.-K. Kim, M.-S. Kim, Y.-K. Choi, *ACS Nano* **2021**, *15*, 258–287.
[100] J. Zhang, S. Lin, M. Zheng, Z. L. Wang, *ACS Nano* **2021**, *15*, 14830–14837.
[101] D. Choi, Y. Lee, Z.-H. Lin, S. Cho, M. Kim, C. K. Ao, S. Soh, C. Sohn, C. K. Jeong, J. Lee, M. Lee, S. Lee, J. Ryu, P. Parashar, Y. Cho, J. Ahn, I.-D. Kim, F. Jiang, P. S. Lee, G. Khandelwal, S.-J. Kim, H. S. Kim, H.-C. Song, M. Kim, J. Nah, W. Kim, H. G. Menge, Y. T. Park, W. Xu, J. Hao, H. Park, J.-H. Lee, D.-M. Lee, S.-W. Kim, J. Y. Park, H. Zhang, Y. Zi, R. Guo, J. Cheng, Z. Yang, Y. Xie, S. Lee, J. Chung, I.-K. Oh, J.-S. Kim, T. Cheng, Q. Gao, G. Cheng, G. Gu, M. Shim, J. Jung, C. Yun, C. Zhang, G. Liu, Y. Chen, S. Kim, X. Chen, J. Hu, X. Pu, Z. H. Guo, X. Wang, J. Chen, X. Xiao, X. Xie, M. Jarin, H. Zhang, Y.-C. Lai, T. He, H. Kim, I. Park, J. Ahn, N. D. Huynh, Y. Yang, Z. L. Wang, J. M. Baik, D. Choi, *ACS Nano* **2023**, *17*, 11087–11219.
[102] S. Wang, L. Lin, Y. Xie, Q. Jing, S. Niu, Z. L. Wang, *Nano Lett.* **2013**, *13*, 2226–2233.
[103] W. Jiang, H. Li, Z. Liu, Z. Li, J. Tian, B. Shi, Y. Zou, H. Ouyang, C. Zhao, L. Zhao, R. Sun, H. Zheng, Y. Fan, Z. L. Wang, Z. Li, *Adv. Mater.* **2018**, *30*, 1801895.
[104] L. Zhang, B. Zhang, J. Chen, L. Jin, W. Deng, J. Tang, H. Zhang, H. Pan, M. Zhu, W. Yang, Z. L. Wang, *Adv. Mater.* **2016**, *28*, 1650–1656.
[105] Z. L. Wang, T. Jiang, L. Xu, *Nano Energy* **2017**, *39*, 9–23.
[106] F.-R. Fan, L. Lin, G. Zhu, W. Wu, R. Zhang, Z. L. Wang, *Nano Lett.* **2012**, *12*, 3109–3114.
[107] F. R. Fan, W. Tang, Z. L. Wang, *Adv. Mater.* **2016**, *28*, 4283–4305.
[108] A. A. Jaber, A. Abu Obaid, S. G. Advani, J. W. Gillespie, *J. Appl. Polym. Sci.* **2024**, *141*, e55058.
[109] S. Lin, L. Xu, C. Xu, X. Chen, A. C. Wang, B. Zhang, P. Lin, Y. Yang, H. Zhao, Z. L. Wang, *Adv. Mater.* **2019**, *31*, 1808197.
[110] B.-Y. Lee, S.-U. Kim, S. Kang, S.-D. Lee, *Nano Energy* **2018**, *53*, 152–159.
[111] Z. Wang, L. Cheng, Y. Zheng, Y. Qin, Z. L. Wang, *Nano Energy* **2014**, *10*, 37–43.
[112] S.-N. Lai, C.-K. Chang, C.-S. Yang, C.-W. Su, C.-M. Leu, Y.-H. Chu, P.-W. Sha, J. M. Wu, *Nano Energy* **2019**, *60*, 715–723.

[113] Y. Hou, X. Dong, W. Tang, D. Li, *Materials* **2023**, *16*, 4970.
[114] A. R. Mule, B. Dudem, S. A. Graham, J. S. Yu, *Adv. Funct. Mater.* **2019**, *29*, 1807779.
[115] Z. Lin, G. Cheng, L. Lin, S. Lee, Z. L. Wang, *Angew. Chem. Int. Ed.* **2013**, *52*, 12545–12549.
[116] D. Kang, J.-H. Hwang, Y.-J. Kim, P. Zhao, H. Yeong Lee, J. Kim, M. S. Shin, S. Jeon, S. Kim, S.-W. Kim, *Mater. Today* **2024**, *72*, 109–116.
[117] D. Alvarez, *J. Large-Scale Res. Facil. JLSRF* **2021**, *7*, A183.
[118] H. E. Gottlieb, V. Kotlyar, A. Nudelman, *J. Org. Chem.* **1997**, *62*, 7512–7515.
[119] C. A. Schneider, W. S. Rasband, K. W. Eliceiri, *Nat. Methods* **2012**, *9*, 671–675.
[120] N. Fairley, V. Fernandez, M. Richard-Plouet, C. Guillot-Deudon, J. Walton, E. Smith, D. Flahaut, M. Greiner, M. Biesinger, S. Tougaard, D. Morgan, J. Baltrusaitis, *Appl. Surf. Sci. Adv.* **2021**, *5*, 100112.
[121] D. Nečas, P. Klapetek, *Open Phys.* **2012**, *10*, 181–188.
[122] A. Hjorth Larsen, J. Jørgen Mortensen, J. Blomqvist, I. E. Castelli, R. Christensen, M. Dułak, J. Friis, M. N. Groves, B. Hammer, C. Hargus, E. D. Hermes, P. C. Jennings, P. Bjerre Jensen, J. Kermode, J. R. Kitchin, E. Leonhard Kolsbjerg, J. Kubal, K. Kaasbjerg, S. Lysgaard, J. Bergmann Maronsson, T. Maxson, T. Olsen, L. Pastewka, A. Peterson, C. Rostgaard, J. Schiøtz, O. Schütt, M. Strange, K. S. Thygesen, T. Vegge, L. Vilhelmsen, M. Walter, Z. Zeng, K. W. Jacobsen, *J. Phys. Condens. Matter* **2017**, *29*, 273002.
[123] J. J. Mortensen, L. B. Hansen, K. W. Jacobsen, *Phys. Rev. B* **2005**, *71*, 035109.
[124] J. J. Mortensen, A. H. Larsen, M. Kuisma, A. V. Ivanov, A. Taghizadeh, A. Peterson, A. Haldar, A. O. Dohn, C. Schäfer, E. Ö. Jónsson, E. D. Hermes, F. A. Nilsson, G. Kastlunger, G. Levi, H. Jónsson, H. Häkkinen, J. Fojt, J. Kangsabanik, J. Sødequist, J. Lehtomäki, J. Heske, J. Enkovaara, K. T. Winther, M. Dulak, M. M. Melander, M. Ovesen, M. Louhivuori, M. Walter, M. Gjerding, O. Lopez-Acevedo, P. Erhart, R. Warmbier, R. Würdemann, S. Kaappa, S. Latini, T. M. Boland, T. Bligaard, T. Skovhus, T. Susi, T. Maxson, T. Rossi, X. Chen, Y. L. A. Schmerwitz, J. Schiøtz, T. Olsen, K. W. Jacobsen, K. S. Thygesen, *J. Chem. Phys.* **2024**, *160*, 092503.
[125] J. Enkovaara, C. Rostgaard, J. J. Mortensen, J. Chen, M. Dułak, L. Ferrighi, J. Gavnholt, C. Glinsvad, V. Haikola, H. A. Hansen, H. H. Kristoffersen, M. Kuisma, A. H. Larsen, L. Lehtovaara, M. Ljungberg, O. Lopez-Acevedo, P. G. Moses, J. Ojanen, T. Olsen, V. Petzold, N. A. Romero, J. Stausholm-Møller, M. Strange, G. A. Tritsaris, M. Vanin, M. Walter, B. Hammer, H. Häkkinen, G. K. H. Madsen, R. M. Nieminen, J. K. Nørskov, M. Puska, T. T. Rantala, J. Schiøtz, K. S. Thygesen, K. W. Jacobsen, *J. Phys. Condens. Matter* **2010**, *22*, 253202.
[126] J. P. Perdew, K. Burke, M. Ernzerhof, *Phys. Rev. Lett.* **1996**, *77*, 3865–3868.
[127] S. Grimme, *J. Comput. Chem.* **2006**, *27*, 1787–1799.
[128] H. J. Monkhorst, J. D. Pack, *Phys. Rev. B* **1976**, *13*, 5188–5192.
[129] G. Makov, M. C. Payne, *Phys. Rev. B* **1995**, *51*, 4014–4022.
[130] I. Dabo, B. Kozinsky, N. E. Singh-Miller, N. Marzari, *Phys. Rev. B* **2008**, *77*, 115139.
[131] J. M. Kahk, G. S. Michelitsch, R. J. Maurer, K. Reuter, J. Lischner, *J. Phys. Chem. Lett.* **2021**, *12*, 9353–9359.
[132] D. G. Castner, K. Hinds, D. W. Grainger, *Langmuir* **1996**, *12*, 5083–5086.
[133] J. E. Sader, J. A. Sanelli, B. D. Adamson, J. P. Monty, X. Wei, S. A. Crawford, J. R. Friend, I. Marusic, P. Mulvaney, E. J. Bieske, *Rev. Sci. Instrum.* **2012**, *83*, 103705.
[134] A. Kolberg, C. Wenzel, T. Hugel, M. Gallei, B. N. Balzer, *J. Vis. Exp.* **2020**, *157*, 60934.
[135] B. V. Derjaguin, V. M. Muller, Yu. P. Toporov, *J. Colloid Interface Sci.* **1975**, *53*, 314–326.
[136] B. N. J. Persson, M. Scaraggi, A. I. Volokitin, M. K. Chaudhury, *EPL Europhys. Lett.*

2013, *103*, 36003.
[137] J. G. Noel, *IET Circuits Devices Syst.* **2016**, *10*, 156–161.
[138] S. Sadewasser, Th. Glatzel, R. Shikler, Y. Rosenwaks, M. Ch. Lux-Steiner, *Appl. Surf. Sci.* **2003**, *210*, 32–36.
[139] E. Palleau, L. Ressier, Ł. Borowik, T. Mélin, *Nanotechnology* **2010**, *21*, 225706.
[140] M. Kihel, S. Sahli, A. Zenasni, P. Raynaud, Y. Segui, *Vacuum* **2014**, *107*, 264–268.
[141] O. Mitsunobu, M. Yamada, *Bull. Chem. Soc. Jpn.* **1967**, *40*, 2380–2382.
[142] E. Ortí, R. Viruela, P. M. Viruela, *J. Phys. Chem.* **1996**, *100*, 6138–6146.
[143] H. Häkkinen, *Nat. Chem.* **2012**, *4*, 443–455.
[144] G. Yang, G. Liu, *J. Phys. Chem. B* **2003**, *107*, 8746–8759.
[145] R. Otero, A. L. Vázquez De Parga, J. M. Gallego, *Surf. Sci. Rep.* **2017**, *72*, 105–145.
[146] P. Zaumseil, *J. Appl. Crystallogr.* **2015**, *48*, 528–532.
[147] M. Yamamoto, T. Matsumae, Y. Kurashima, H. Takagi, T. Suga, T. Itoh, E. Higurashi, *Micromachines* **2019**, *10*, 119.
[148] D. G. Castner, K. Hinds, D. W. Grainger, *Langmuir* **1996**, *12*, 5083–5086.
[149] T. R. Gengenbach, G. H. Major, M. R. Linford, C. D. Easton, *J. Vac. Sci. Technol. A* **2021**, *39*, 013204.
[150] B. Snopok, A. Laroussi, C. Cafolla, K. Voïtchovsky, T. Snopok, V. M. Mirsky, *Surf. Interfaces* **2021**, *22*, 100818.
[151] P. Bhadra, S. W. I. Siu, *Langmuir* **2021**, *37*, 1913–1924.
[152] N. Sagawa, T. Shikata, *Phys Chem Chem Phys* **2014**, *16*, 13262–13270.
[153] S. Alvarez, *Dalton Trans.* **2013**, *42*, 8617.
[154] N. Turetta, F. Sedona, A. Liscio, M. Sambi, P. Samorì, *Adv. Mater. Interfaces* **2021**, *8*, 2100068.
[155] X. Xu, A. Makaraviciute, J. Pettersson, S.-L. Zhang, L. Nyholm, Z. Zhang, *Sens. Actuators B* **2019**, *283*, 146–153.
[156] D. Bengio, E. Mendes, S. Pellet-Rostaing, P. Moisy, *J. Electroanal. Chem.* **2018**, *823*, 445–454.
[157] G. Roy, R. Gupta, S. Ranjan Sahoo, S. Saha, D. Asthana, P. Chandra Mondal, *Coord. Chem. Rev.* **2022**, *473*, 214816.
[158] C. J. Slevin, S. Ryley, D. J. Walton, P. R. Unwin, *Langmuir* **1998**, *14*, 5331–5334.
[159] M. Binggeli, C. M. Mate, *J. Vac. Sci. Technol. B* **1995**, *13*, 1312–1315.
